# Four new species of *Gasteruption* Latreille from NW China, with an illustrated key to the species from Palaearctic China (Hymenoptera, Gasteruptiidae)

**DOI:** 10.3897/zookeys.612.9751

**Published:** 2016-08-23

**Authors:** Jiang-Li Tan, Cornelis van Achterberg, Qing-Qing Tan, Xue-Xin Chen

**Affiliations:** 1 Shaanxi Key Laboratory for Animal Conservation / Key Laboratory of Resource Biology and Biotechnology in Western China, College of Life Sciences, Northwest University, 229 North Taibai Road, Xi’an, Shaanxi 710069, China; 2 Institute of Insect Sciences, Zhejiang University, Zijingang Campus, Yuhangtang Road 866, Hangzhou 310058, China

**Keywords:** Gasteruption, Heilongjiang, Inner Mongolia, key, Mongolia new species, new record, Ningxia, Shaanxi

## Abstract

Four new species of the genus *Gasteruption* Latreille, 1796 (Hymenoptera: Evanioidea: Gasteruptiidae: Gasteruptiinae) are reported from NW China: three from Shaanxi province and one from Ningxia province. The new species (*Gasteruption bicoloratum* Tan & van Achterberg, **sp. n.**, *Gasteruption huangshii* Tan & van Achterberg, **sp. n.**, *Gasteruption pannuceum* Tan & van Achterberg, **sp. n.**, and *Gasteruption shengi* Tan & van Achterberg, **sp. n.**) and three newly recorded species (*Gasteruption sinepunctatum* Zhao, van Achterberg & Xu, 2012, *Gasteruption boreale* (Thomson, 1883) and *Gasteruption oshimense* Watanabe, 1924) are keyed and fully illustrated. In total, seven species are known from Shaanxi province, which is approximately half of the expected number. The East Palaearctic specimens provisionally identified as *Gasteruption tournieri* Schletterer, 1885, by Zhao et al. (2012) are included under *Gasteruption oshimense* Watanabe, 1924.

## Introduction

The family Gasteruptiidae is a small group of wasps comprising about 500 described species in two subfamilies, Gasteruptiinae (four genera) (Macedo 2009, 2011; Zhao et al. 2012) and Hyptiogastrinae (two genera) (Jennings and Austin 2002). Gasteruptiidae are easily distinguished from the other apocritan hymenopterans by the elongated “neck” (propleuron), the swollen hind tibiae, and the highly attached and slender metasoma. Adults are free-living insects normally feeding on nectar from flowers with easily accessible nectar (especially families Apiaceae, Asteraceae and Euphorbiaceae), but likely at least some *Gasteruption* species feed on both nectar and pollen (Jennings and Austin 2004). Gasteruptiidae are also known by their hovering inspection flight in front of bee nests (van Achterberg 2013). The larvae feed on the larval food of solitary bees, after consuming the egg or larva of the bee (Malyshev 1966). They select bees of the subfamilies Apinae, Colletinae and Megachilinae nesting in stems or in wood, and less often in clay banks or other vertical soil substrates (Malyshev 1965; Zhao et al. 2012; van Achterberg 2013); as far as known, bees nesting in horizontal soil substrates are far less attacked. However, in Australia members of the Hyptiogastrinae do attend bee nests in flat ground (Houston 1987). There is only indirect evidence that Gasteruptiinae may attack wasp nests, especially of Crabronidae, Sphecidae and solitary Vespidae (Eumeninae) (Crosskey 1951; Gauld and Hanson 1995, Hanson and Gauld 2006; Jennings and Austin 1997a, 1997b, 2004). Metamorphosis takes place inside the host’s nest where the gasteruptiid pupa hibernates until the next spring or summer (Malyshev 1968; He 2004; Jennings and Austin 2004). All known gasteruptiids from the Palaearctic Region belong to the subfamily Gasteruptiinae and to the genus *Gasteruption* Latreille, 1796. For identification the revision of the Chinese species (Zhao et al. 2012) and the yet unpublished revision of the East Palaearctic species (van Achterberg, in prep.) were used. According to Zhao et al. (2012) two species (*Gasteruption angulatum* Zhao, van Achterberg & Xu, 2012 and *Gasteruption japonicum* Cameron, 1888) are known from the NW Chinese province Shaanxi, which is 7% of the total of 28 species reported from China. Italy (about as varied in natural habitats as Shaanxi) is 50% larger than Shaanxi but has 20 species reported, which is about 60% of the total species known from Europe. In this paper we report five additional species, and from the comparison with Italy it may be deduced these seven are about half of the number to be expected.

## Material and methods

The specimens were mainly collected by hand net or sweep netting, rarely in Malaise traps during 2015. Specimens from Shaanxi were directly stored in 70% ethanol, prepared using the AXA method (van Achterberg 2009; van Achterberg et al. 2010) and glued on card points; other specimens were directly pinned. Observations and descriptions were made with an Olympus SZX11 stereomicroscope and fluorescent lamps. Photographic images were made either with a Keyence VHX-5000 digital microscope or with an Olympus motorized stereomicroscope SZX12 and processed with Adobe Photoshop CS5, mostly to adjust the size and background.

**Figure A–B.**
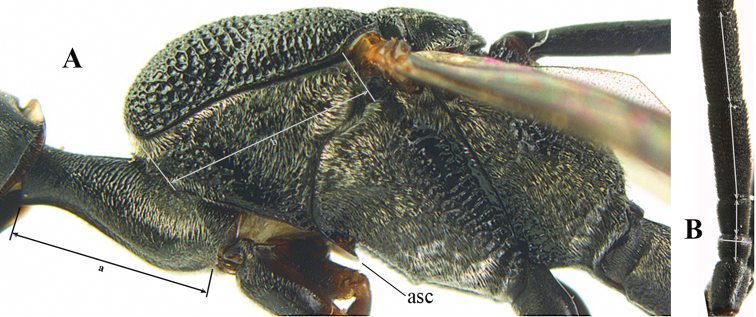
Measurements (**A**) of the relative length of the propleuron (a) and length of the mesoscutum in front of the tegulum (b) and (**B**) the length and maximum width of the basal antennal segments; asc = antesternal carina.

For comparison of head shapes it is essential that the middle of the vertex is in plane of objective of binocular microscope. For the other terminology, see Zhao et al. (2012) and van Achterberg (1988). Measurements are performed as indicated in Fig. A–B and in van Achterberg (1988). Additional non-exclusive characters in the key are between square brackets. The association of males with females is based on similarity. In the few cases in which no males were available, distinctive, and probably non-sexual, characters of the female were tentatively used for inclusion in the key. A new provincial record is indicated by an asterisk. The following abbreviations are used for the depositories:

ECHU
 Entomology Collection, Hokkaido University, Sapporo 


NWUX
 College of Life Sciences, Northwest University, Xi’an 


RMNH
Naturalis Biodiversity Center, Leiden 


ZJUH
 Parasitic Hymenoptera Collection of the Zhejiang University, Hangzhou 


## Results

### Gasteruption

Taxon classificationAnimaliaHymenopteraGasteruptiidae

Latreille, 1796

Gasteruption Latreille, 1796: 113; Zhao et al. 2012: 6–7 (diagnosis, references, key); van Achterberg 2013: 59 (illustrated key for the Netherlands); Jennings and Parslow 2014: 95; van Achterberg and Talebi 2014: 10 (illustrated key for Iran and Turkey); Žikić et al. 2014: 573. Type-species (designated by Latreille 1810): Ichneumon assectator Linnaeus, 1758.

## Key to species of the genus *Gasteruption* Latreille from Palaearctic China

**Table:** 

1	Ovipositor present (a); antenna with 14 segments (b) (females)	**2**
	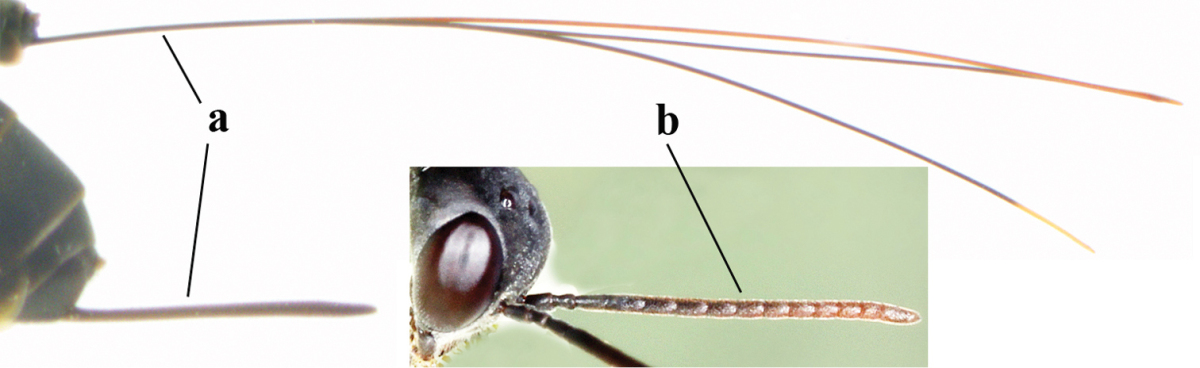	
–	Ovipositor absent (aa); antenna with 13 segments (bb) (males); [if males are unknown the species is provisionally included, see Materials and methods]	**32**
	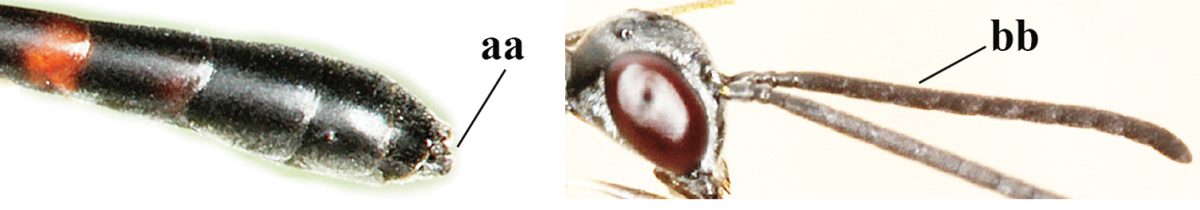	
2	Apex of ovipositor sheath entirely blackish or dark brown, OR, if narrowly pale apically, then white, ivory or brownish yellow part at most 0.3 times as long as hind basitarsus (a); (intermediate species are included in both alternatives)	**3**
	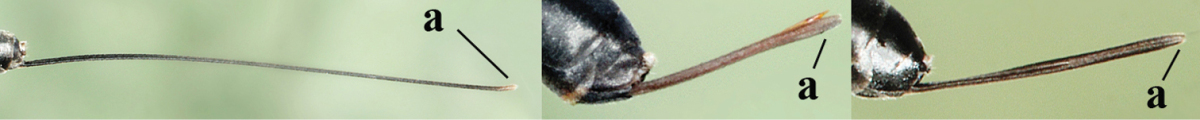	
–	Apex of ovipositor sheath distinctly white or ivory (but rarely pale brown) and pale part 0.3–8.0 times as long as hind basitarsus (aa)	**21**
	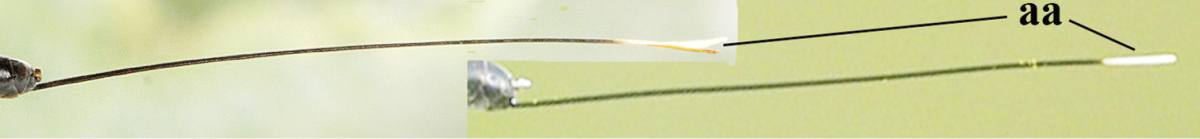	
3	Ovipositor sheath 0.6–1.9 times as long as hind tibia and 0.3–1.2 times as long as hind tibia and tarsus combined (a); incision of hypopygium shallow, V-shaped and extending to apical 0.2 (b); occipital carina obsolescent to narrowly lamelliform medio-dorsally (c)	**4**
	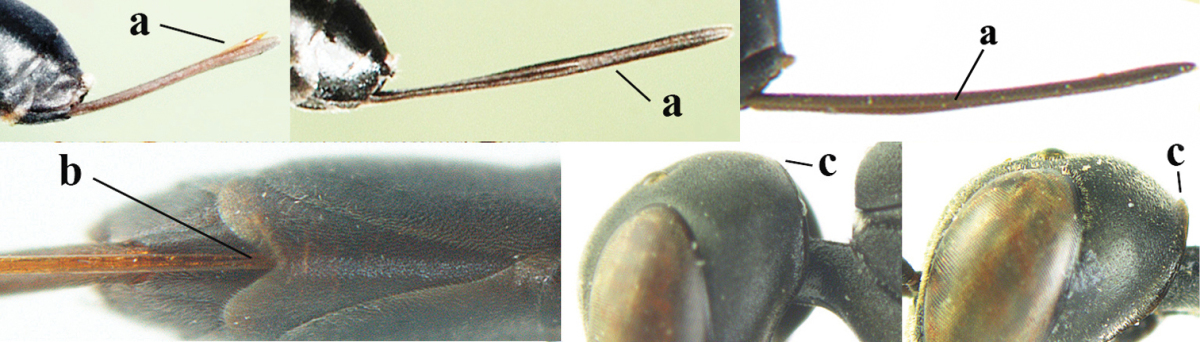	
–	Ovipositor sheath 3.0–7.0 times as long as hind tibia and 1.9–4.0 times as long as hind tibia and tarsus combined (aa); incision of hypopygium often deep and slit-like and extending to apical 0.3–0.5 (bb); occipital carina obsolescent (c) or distinctly lamelliform (cc) medio-dorsally	**14**
	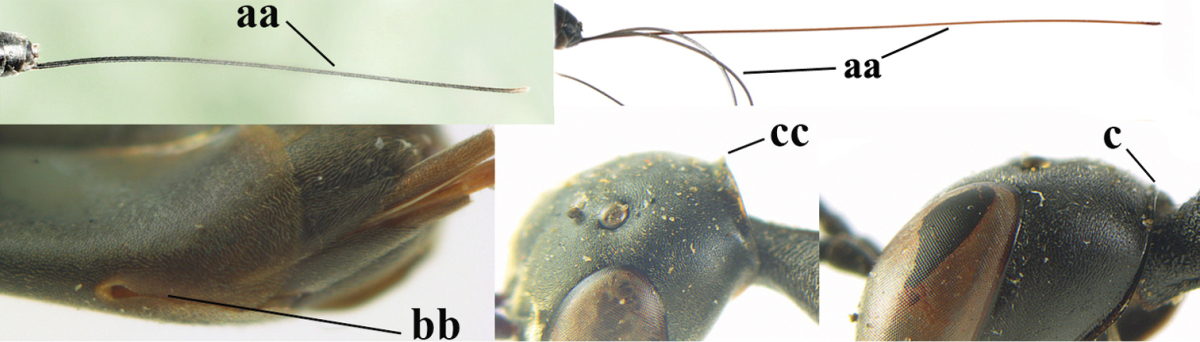	
4	Malar space in anterior view 0.5–0.6 times length of pedicellus and 0.4–0.6 times basal width of mandible and mandibular condylus distinctly below lower level of eyes (a); in lateral view condylar incision of malar space remains far removed from eye (b); ovipositor sheath 0.4–0.9 times as long as hind tibia (c); first discal cell of fore wing usually suddenly narrowed (d)	***Gasteruption oriplanum* Kieffer, 1914**
	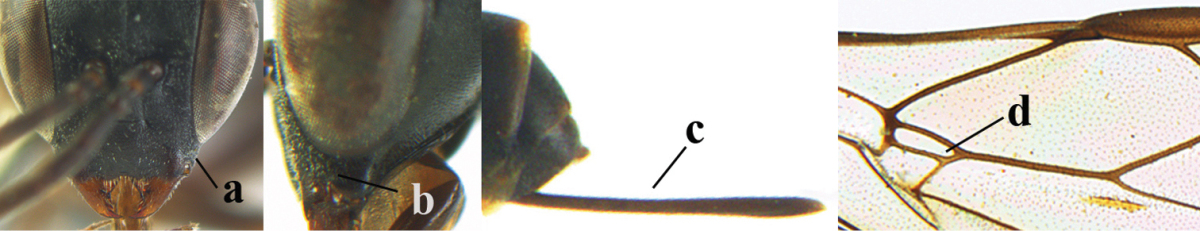	
–	Head in anterior view slightly protruding below lower level of eyes by less than half basal width of mandible and mandibular condylus near lower level of eyes (aa); in lateral view condylar incision of malar space close to eye (bb); ovipositor sheath 0.8–2.7 times as long as hind tibia (cc); first discal cell of fore wing usually gradually narrowed (dd)	**5**
	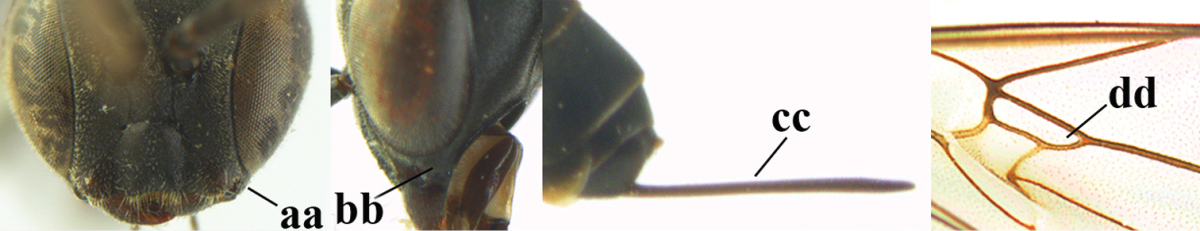	
5	Clypeus with rather large shallow depression (a); mesoscutum densely reticulate-rugulose or -rugose (b); hind basitarsus stout (c); apical antennal segment 1.4–1.6 times third antennal segment (d)	***Gasteruption formilis* Alekseev, 1995**
	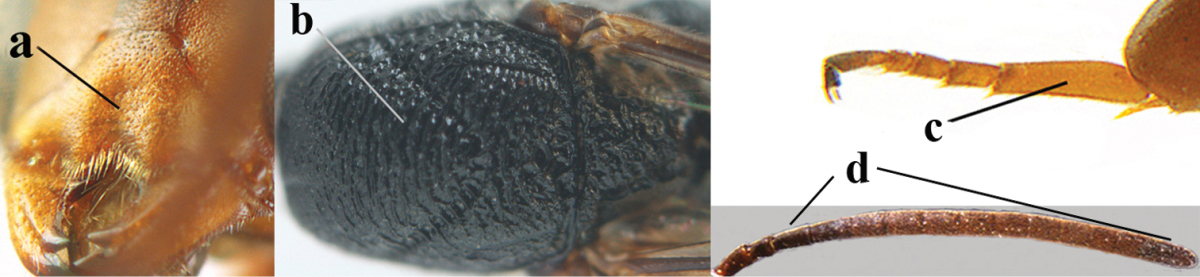	
–	Clypeus with small depression or depression obsolescent (aa); mesoscutum mainly densely coriaceous or rugulose (bb); hind basitarsus slender (cc), rarely similarly stout; apical antennal segment at most 1.2 times as long as third antennal segment (dd)	**6**
	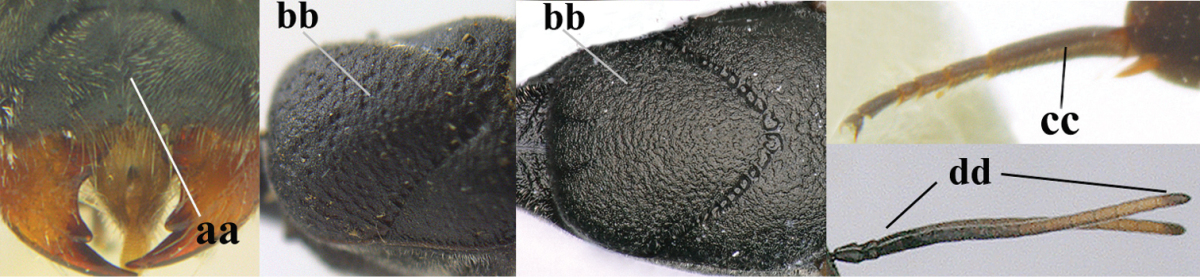	
6	Mesoscutum coarsely (often “crater”-like) punctate (a); head distinctly emarginate medio-posteriorly (b); head less protruding in lateral view (c) and narrower in anterior view (d)	**7**
	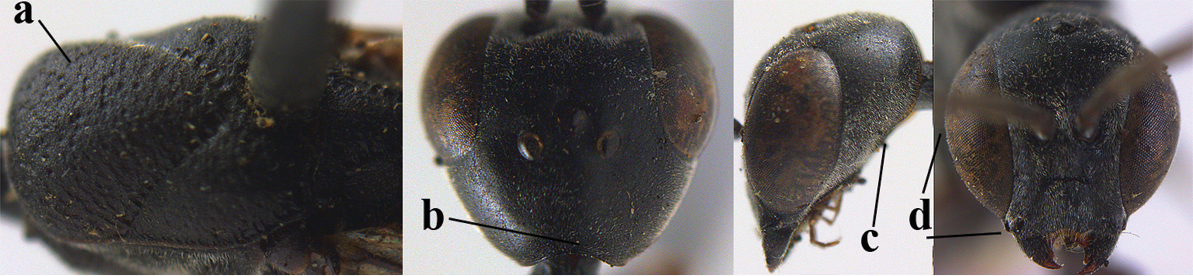	
–	Mesoscutum predominantly densely coriaceous, at most with some shallow punctures (aa); head truncate medio-posteriorly or nearly so (bb); head more protruding in lateral view (cc) and wider in anterior view (dd)	**8**
	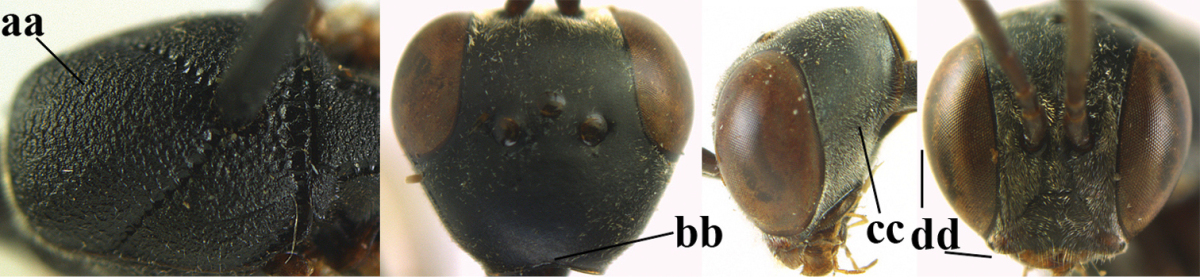	
7	Hind tibia about as long as hind femur and trochanter combined or slightly longer (a); head somewhat longer in dorsal (b) and lateral (c) view; head directly narrowed behind eyes in dorsal view (d)	***Gasteruption formosanum* Enderlein, 1913**
	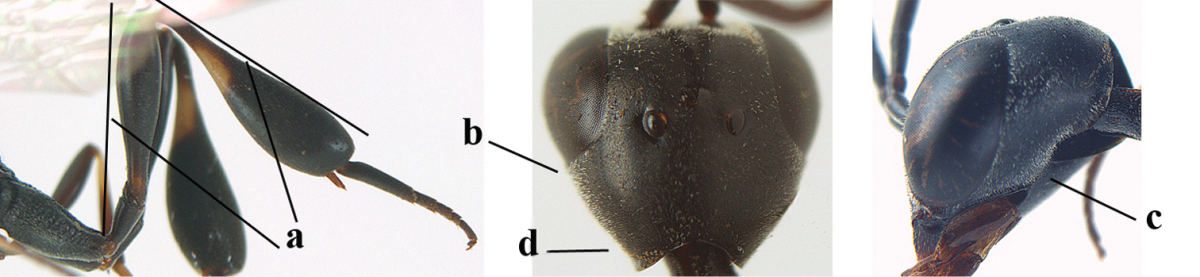	
–	Hind tibia 1.1–1.2 times as long as hind femur and trochanter combined (aa); head somewhat shorter in dorsal (bb) and lateral (cc) view; head roundly narrowed behind eyes in dorsal view (dd)	***Gasteruption sinicola* (Kieffer, 1924)**
	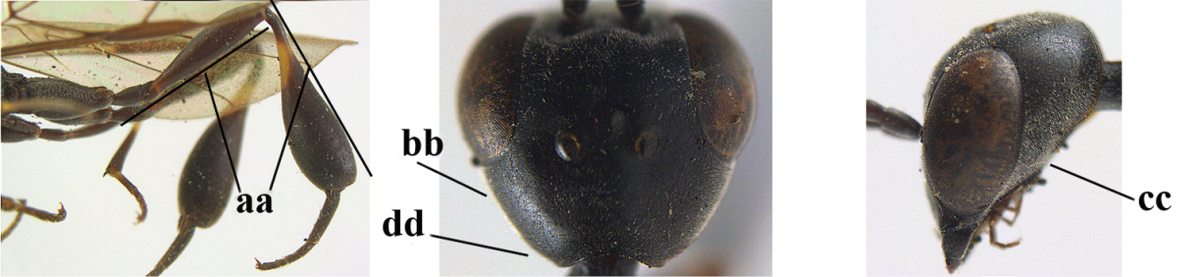	
8	Hind tibia comparatively slender (a); side of pronotum slender and with narrow and weakly crenulated grooves (b); propleuron granulate antero-dorsally (c); [hind basitarsus slender; vertex distinctly punctulate or very finely coriaceous]	***Gasteruption parvicollarium* Enderlein, 1913**
	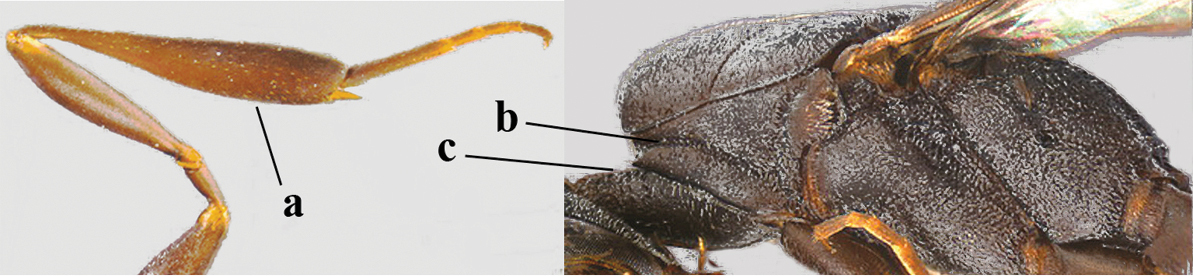	
–	Hind tibia distinctly inflated (aa); side of pronotum robust and with wider and distinctly crenulated grooves (bb); propleuron rugulose or coriaceous antero-dorsally (cc)	**9**
	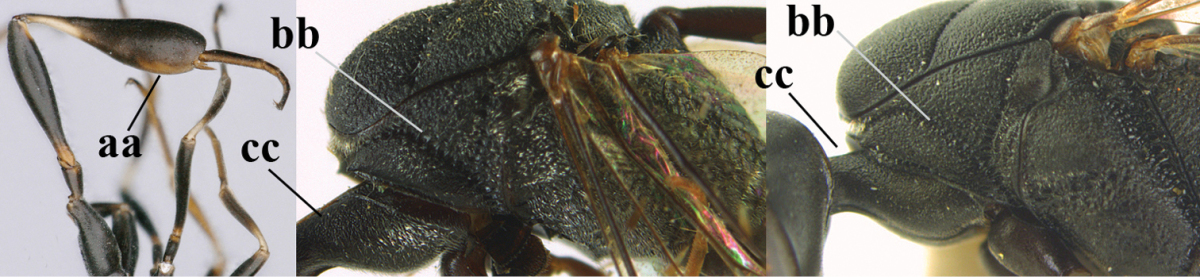	
9	Mandible dark brown or reddish brown basally (a), rarely brownish yellow; basal depression of mandible rather large and deep (b); fifth (= pre-apical) sternite dark brown or blackish or narrowly pale medio-apically (c)	**10**
	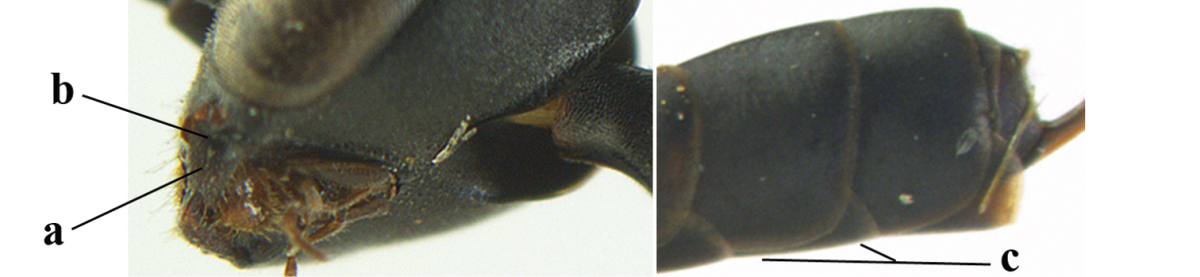	
–	Mandible pale yellow basally (aa); basal depression of mandible often smaller and shallower (bb); fifth sternite yellowish brown medio-apically (cc)	**12**
	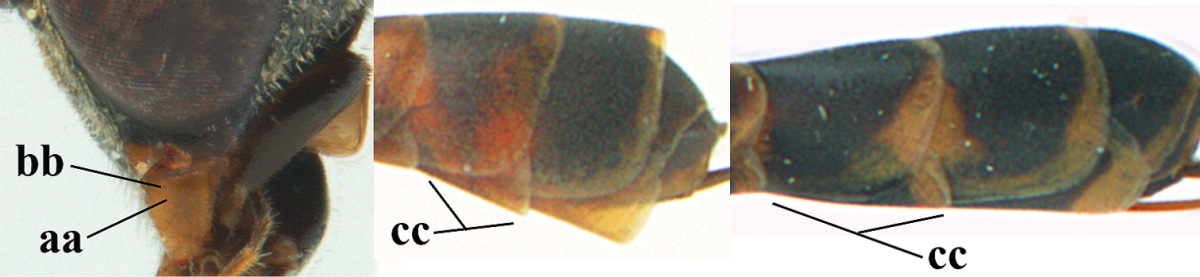	
10	Vertex matt or with satin sheen and densely coriaceous (a); mesoscutum densely coriaceous and without indication of punctures (b); head gradually narrowed posteriorly in dorsal view (c); antero-ventral tooth of pronotum absent or indistinct (d); [third antennal segment 1.5–1.7 times as long as pedicellus; ovipositor sheath only with short fine setae]	**11**
	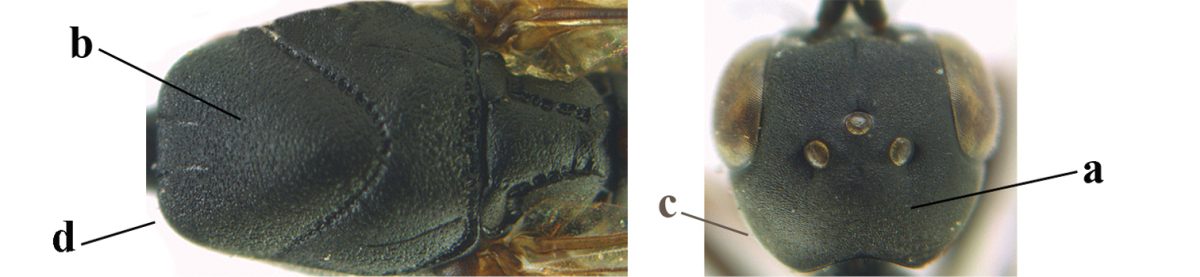	
–	Vertex shiny and punctulate (aa); mesoscutum with some fine punctures (bb); head directly narrowed posteriorly in dorsal view (cc); antero-ventral tooth of pronotum distinct (dd); [occipital carina distinct medio-dorsally]	***Gasteruption latitibia* Zhao, van Achterberg & Xu, 2012**
	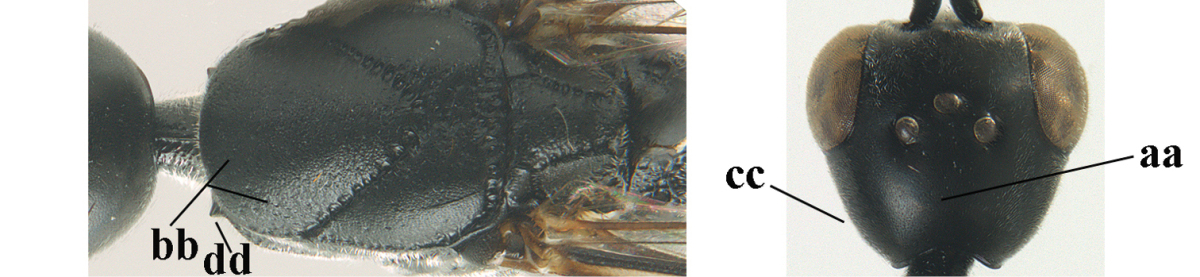	
11	Apical half of ovipositor sheath with dense and adpressed setosity (a); sculpture of mesoscutum distinctly coarser than that of vertex (b); ovipositor sheath 1.1–1.3 times as long as hind tibia (c); head more narrowed behind eyes in dorsal view (d)	**(Linnaeus, 1758)**
	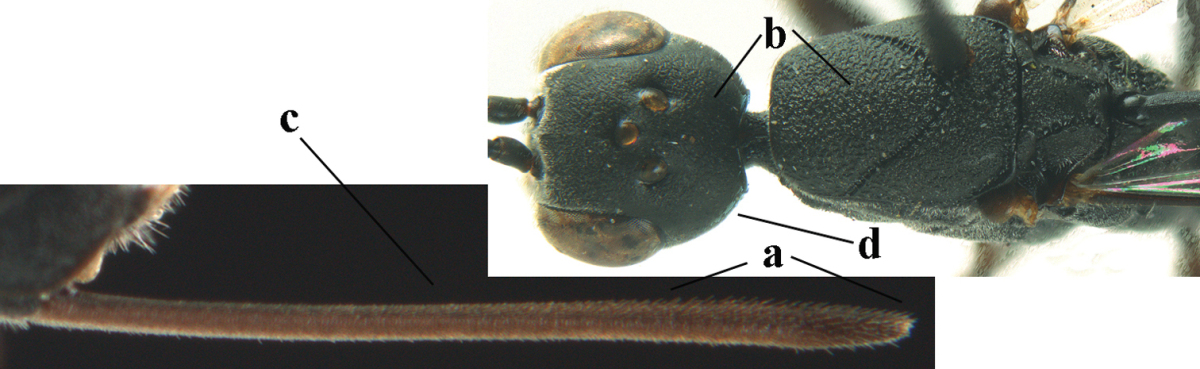	
–	Apical half of ovipositor sheath with erect bristles, angled at about 45º (aa); sculpture of mesoscutum slightly coarser than that of vertex (bb); ovipositor sheath 0.7–1.0 times as long as hind tibia (cc); head less narrowed in dorsal view (dd)	***Gasteruption boreale* (Thomson, 1883)**
	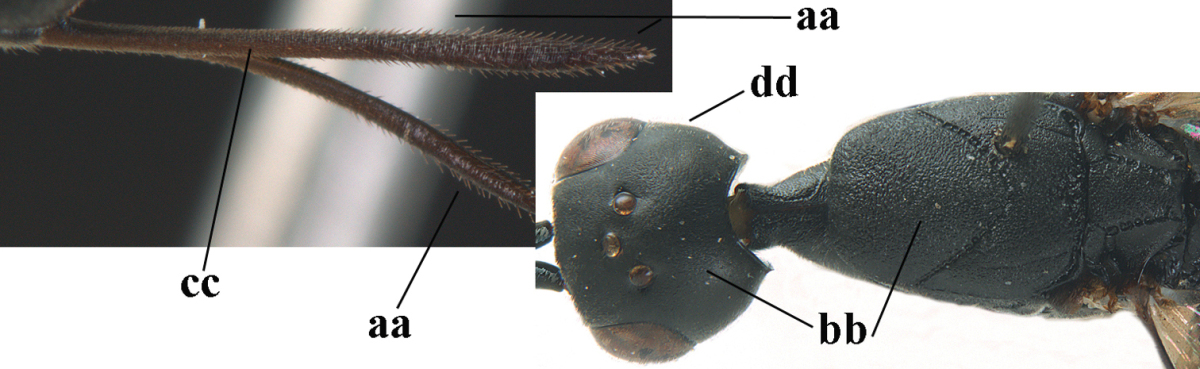	
12	Head longer and directly narrowed in dorsal view (a); posteriorly vertex moderately convex in lateral view (b); metasoma mainly black or dark brown (c); tegula dark brown (d)	***Gasteruption terebrelligerum* Enderlein, 1913**
	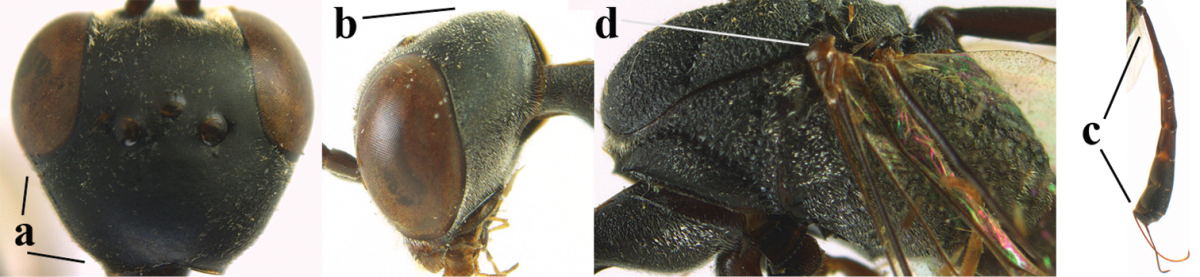	
–	Head shorter and less directly narrowed in dorsal view (aa); vertex flattened in lateral view (bb); metasoma with distinctly yellowish pattern (cc); tegula yellow (dd)	**13**
	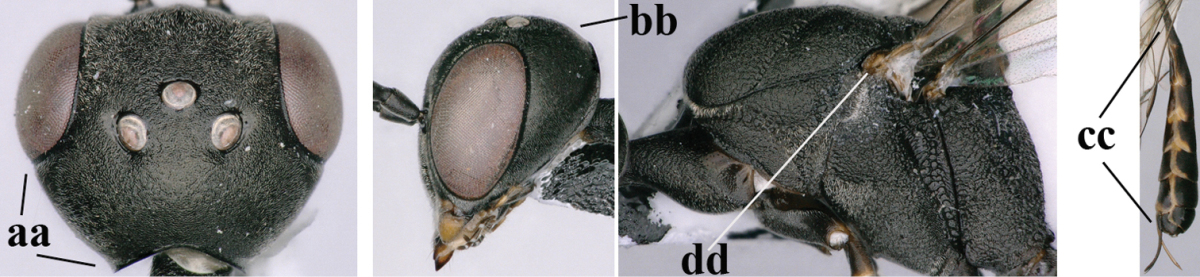	
13	Head not protruding below eyes (a) and malar space 0.3 times length of second antennal segment and 0.2 times basal width of mandible and mandibular condylus close to lower level of eyes (b); hind basitarsus rather stout and at least partly ivory dorsally (c); hind tibia dark ventrally, similar to colour dorsally (d); mesoscutum somewhat coarser sculptured (e)	***Gasteruption flavimarginatum* van Achterberg, 2014**
	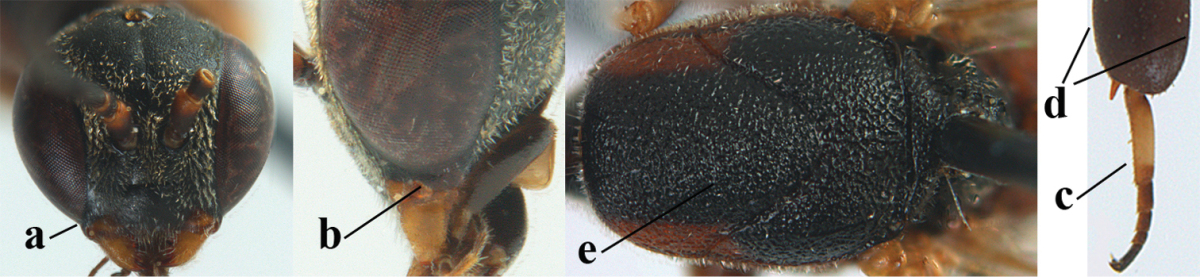	
–	Head somewhat protruding below eyes (aa) and malar space 0.5 times length of second antennal segment and 0.4 times basal width of mandible and mandibular condylus below lower level of eyes (bb); hind basitarsus slender and entirely dark brown dorsally (cc); hind tibia ventrally paler than dorsally (dd); mesoscutum finely sculptured (ee)	***Gasteruption bicoloratum* sp. n.**
	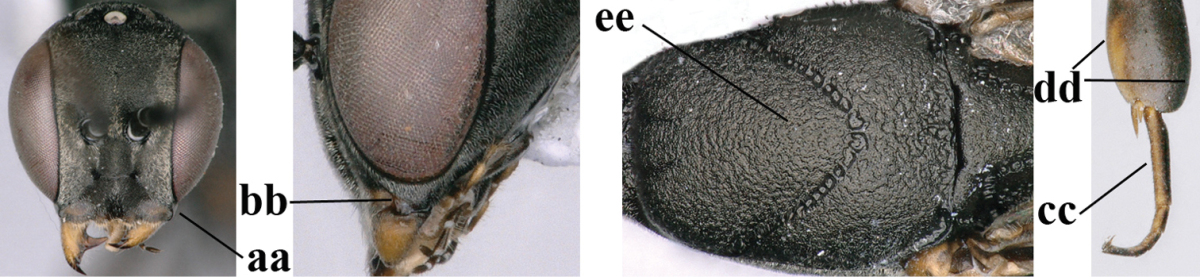	
14	Vertex with reversed V-shaped emargination medio-posteriorly (a), flat (b) **and** smooth, shiny and long dorsally (c); mesoscutum mainly moderately transversely rugose (d)	***Gasteruption bimaculatum* Pasteels, 1958**
	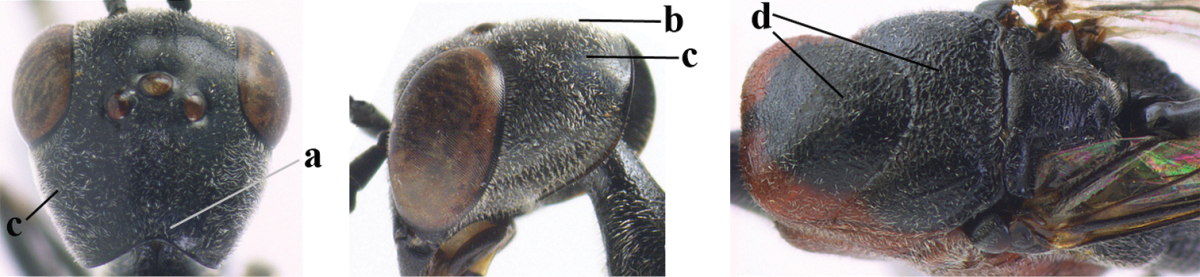	
–	Vertex truncate medio-posteriorly (aa) or reversed U-shaped emarginate (aaa), shorter and moderately convex (bb); **if** vertex more or less emarginate and/or flat, then vertex finely sculptured, with satin sheen and shorter (cc); mesoscutum punctate, punctate-rugose or transversely wrinkled (dd)	**15**
	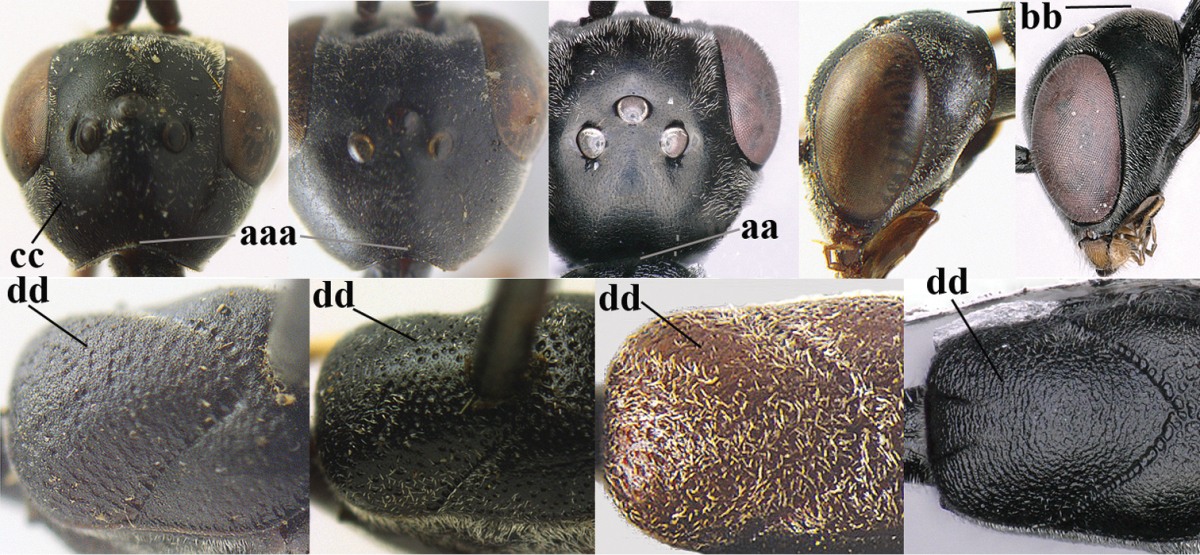	
15	Head rather elongate and below eyes slightly enlarged, minimum length of malar space 0.3–0.4 times second antennal segment (a); head distinctly reversed U-shaped emarginate medio-posteriorly (b); mandible brown (c); hind tarsus brownish apically, paler than basally (d); [apex of ovipositor sheath ivory; first metasomal tergite orange or yellowish brown]	***Gasteruption dimidiatum* Semenov, 1892**
	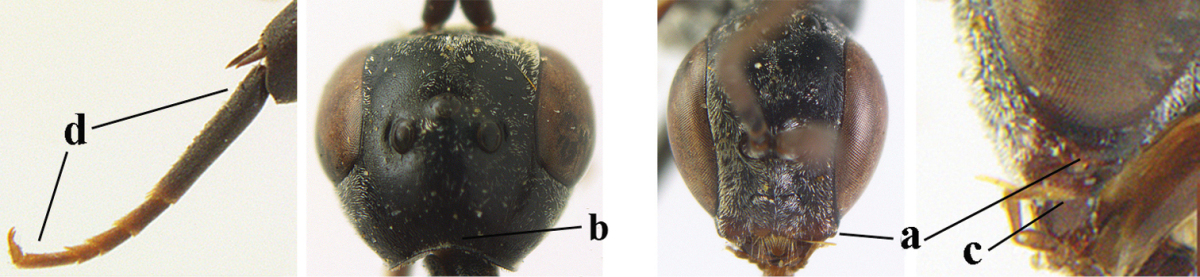	
–	Head less elongate and below eyes not enlarged, minimum length of malar space 0.1–0.2 times second antennal segment (aa); head shallowly emarginate medio-posteriorly (bb); if intermediate (bbb) then mandible brownish yellow (cc); apically hind tarsus dark brown as basally (dd); [apex of ovipositor sheath dark brown or black]	**16**
	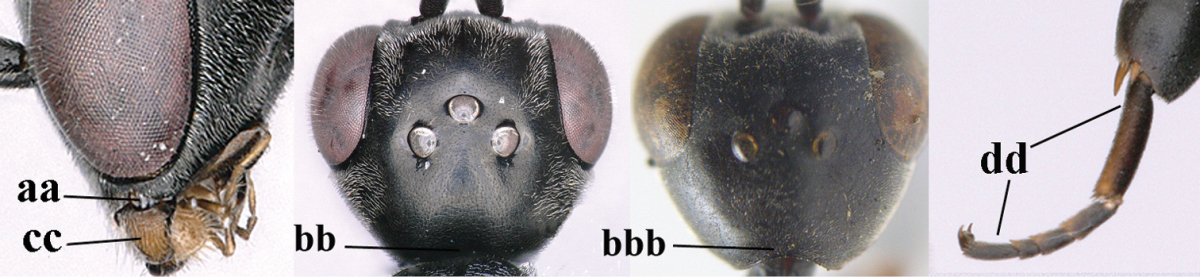	
16	Hind tibia slender (a); hind femur black or blackish brown (b); middle lobe of mesoscutum finely sculptured (c); scapus ventrally and third antennal segment black (d); [length of ovipositor sheath 3–6 times as long as hind tibia; scutellum coriaceous]	**17**
	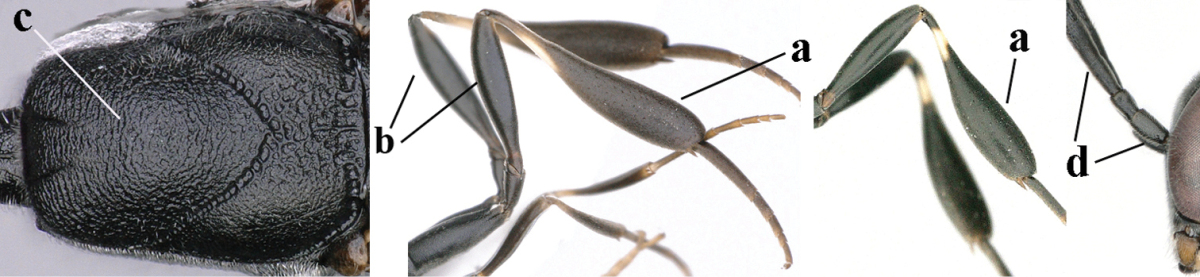	
–	Hind tibia distinctly inflated (aa); hind femur orange brown to dark brown (bb); middle lobe of mesoscutum punctate or punctate-rugose (cc); scapus ventrally paler than third antennal segment or both dark brown (dd)	**18**
	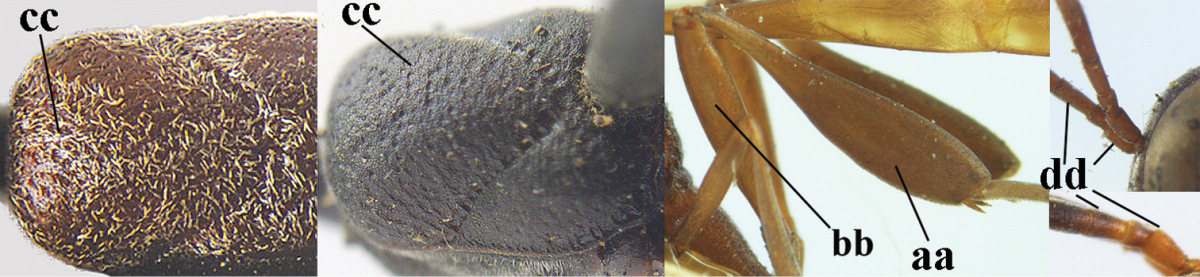	
17	Middle lobe of mesoscutum transversely wrinkled (a); apex of ovipositor sheath ivory (b); apical half of hypopygium largely blackish (c); [ovipositor sheath about 3 times as long as hind tibia]	***Gasteruption pannuceum* sp. n.**
	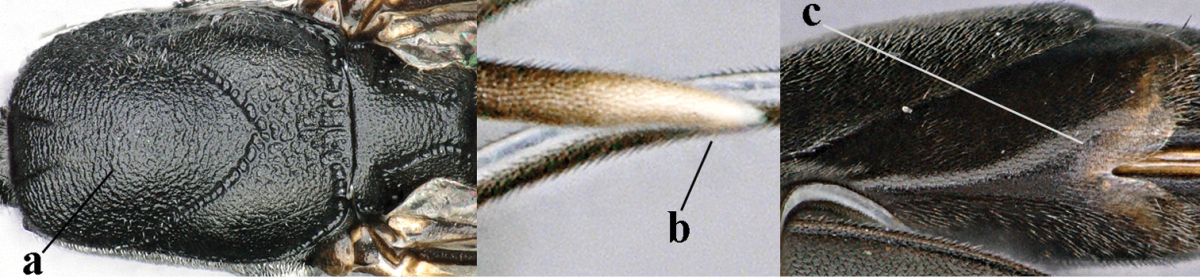	
–	Middle lobe of mesoscutum coriaceous between punctures (aa); apex of ovipositor sheath blackish (bb); apical half of hypopygium brown (cc) or yellowish brown; [ovipositor sheath 4–6 times as long as hind tibia]	***Gasteruption shengi* sp. n.**
	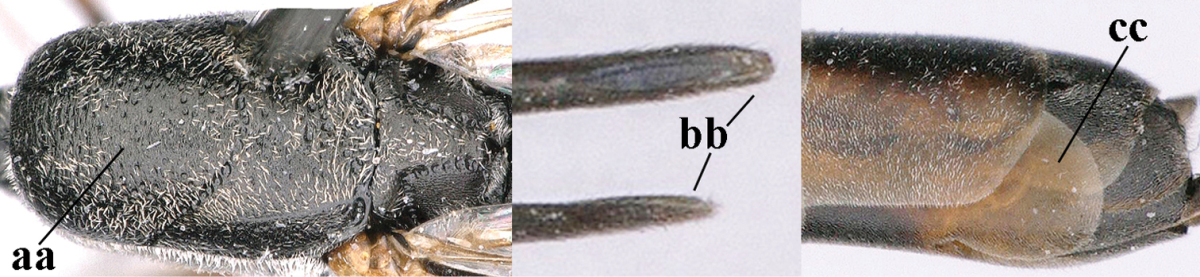	
18	Apex of ovipositor sheath ivory, brownish-yellow or brown and pale part 0.3–1.0 times as long as hind basitarsus (a); mesosoma sparsely setose laterally (b); hind femur dark brown or brown (c); [ovipositor sheath 4.8–6.0 times as long as hind tibia]	***Gasteruption sinarum* Kieffer, 1911**
	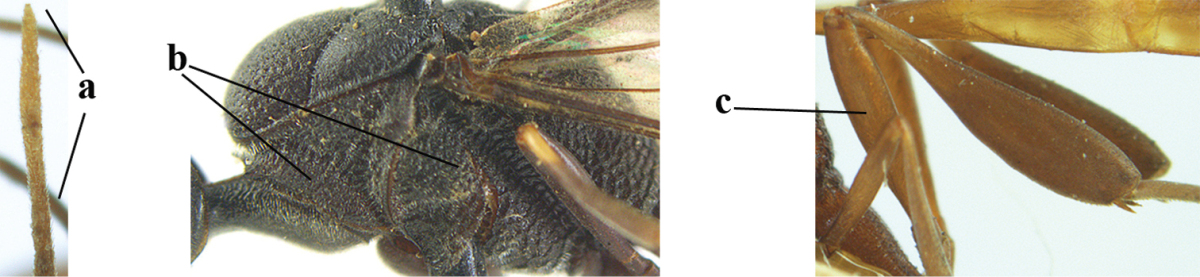	
–	Apex of ovipositor sheath mainly dark brown (aa), pale part at most 0.3 times as long as hind basitarsus; mesosoma densely setose laterally (bb); hind femur orange or reddish brown (cc); [head darker than mesoscutum anteriorly]	**19**
	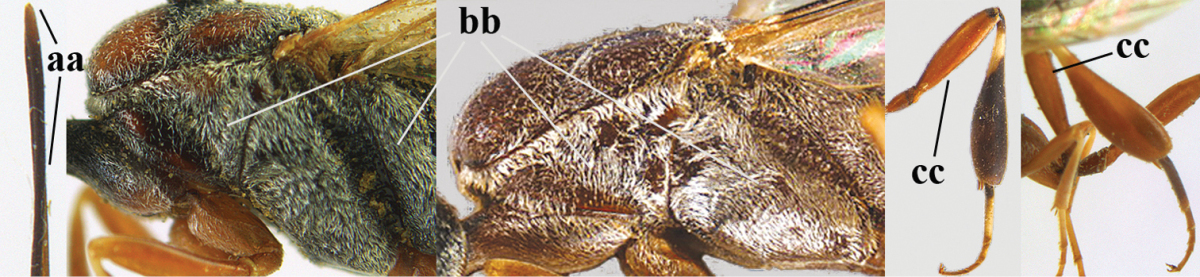	
19	Ovipositor sheath 3.1–4.4 times as long as hind tibia (a); mesoscutum often bicoloured (b); vertex densely setose (c); mesosoma largely or entirely black laterally (d); [hind femur and tibia (except basally) similarly coloured, orange brown or dark brown]	***Gasteruption dilutum* Semenov, 1892**
	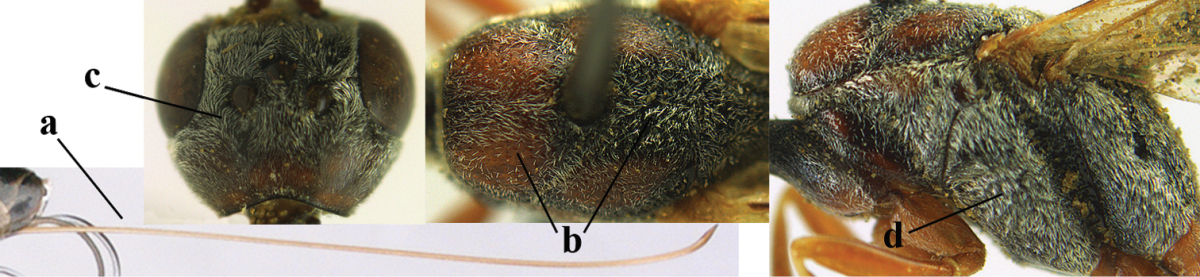	
–	Ovipositor sheath about 7.0 times as long as hind tibia (aa); mesoscutum unicoloured (bb); vertex sparser setose (cc); mesosoma dark reddish or orange brown laterally (dd)	**20**
	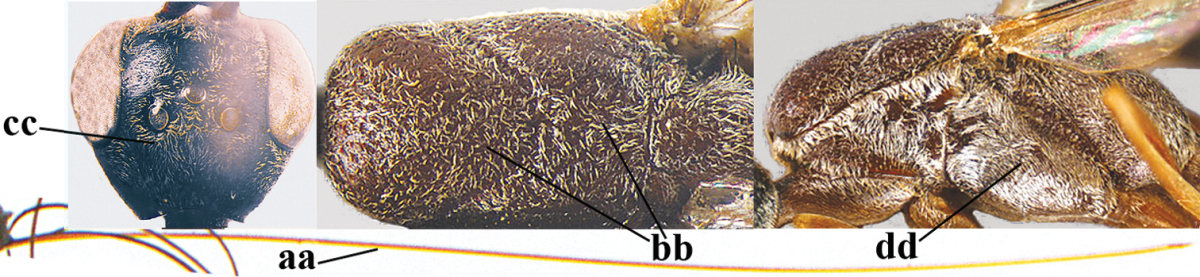	
20	Ventral half of outer side of hind tibia dark brown or blackish except well differentiated subbasal ivory part (a); basal half of hind coxa mainly transversely rugose dorsally (b); apex of ovipositor sheath dark brown (c); propleuron, mesoscutum and pronotum similarly dark brown (d)	***Gasteruption coloratum* Zhao, van Achterberg & Xu, 2012**
	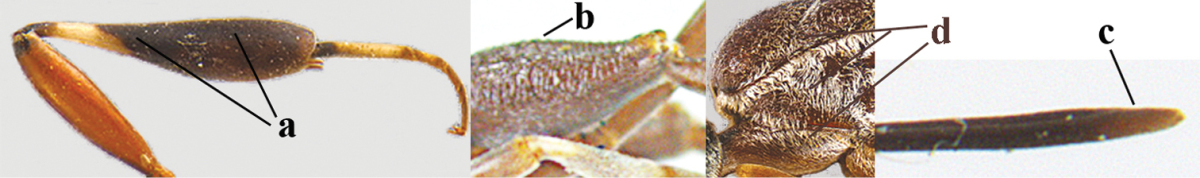	
–	Outer side of hind tibia partly reddish brown and ivory subbasal part less defined (aa); basal half of hind coxa superficially coriaceous dorsally (bb); apex of ovipositor sheath ivory or brownish yellow (cc); mesoscutum and pronotum distinctly paler than propleuron (dd)	***Gasteruption argentifrons* Semenov-T.-S. & Kostylev, 1928**
	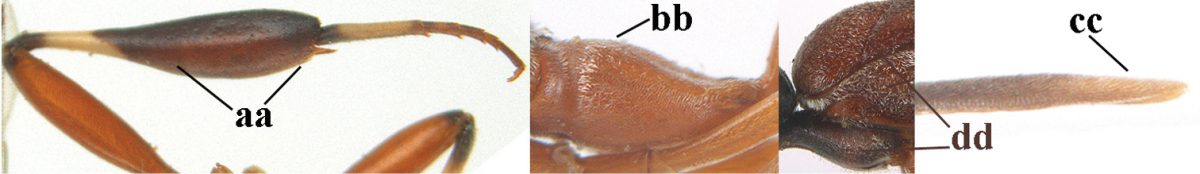	
21	Head with comparatively wide medial depression in front of occipital carina and with pair of lateral depressions (a); occipital carina wide lamelliform (b); medially mesoscutum distinctly transversely punctate-rugulose (c)	***Gasteruption oshimense* Watanabe, 1934**
	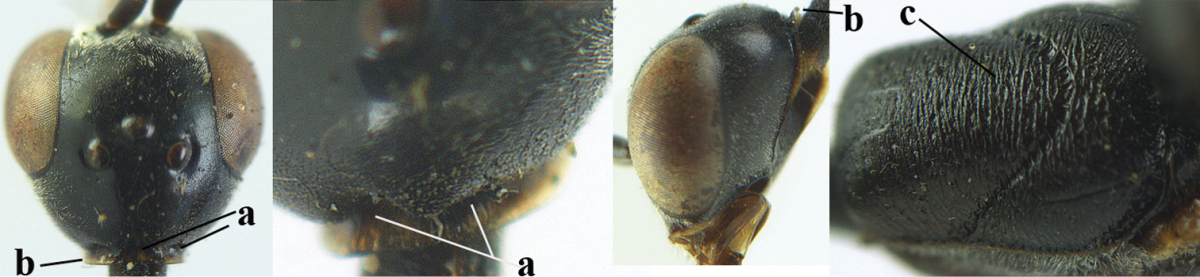	
–	Head flat or evenly convex in front of occipital carina (aa), **if** with a shallow depression in front of occipital carina (aaa), then occipital carina at most moderately lamelliform (bb) and mesoscutum with coarser transverse rugae or punctation medially (cc)	**22**
	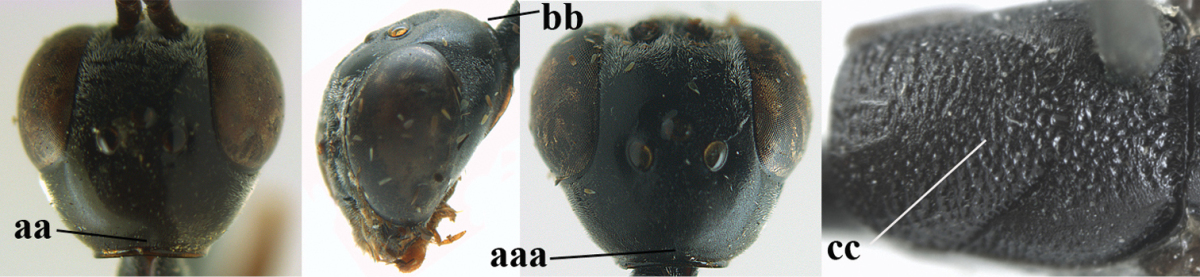	
22	Ovipositor sheath comparatively wide and about 0.9 times as long as hind tibia, 0.3 times as long as metasoma and 0.2 times as long as body (a); middle lobe of mesoscutum rather protuberant in lateral view (b); pronotal tooth slender and acute	***Gasteruption assectoides* Zhao, van Achterberg & Xu, 2012**
	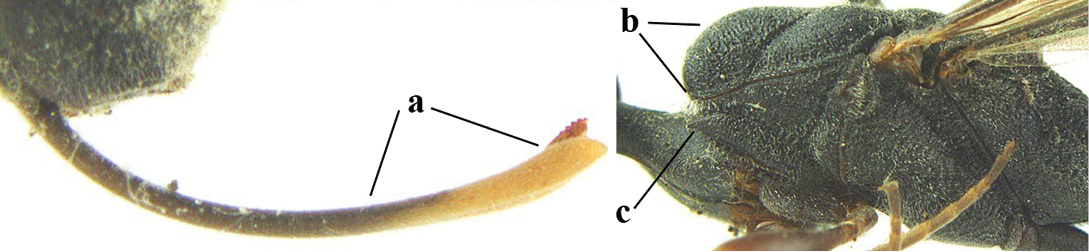	
–	Ovipositor sheath comparatively narrow and 1.1–9.0 times as long as hind tibia, 0.6–2.8 times as long as metasoma and 0.4–1.4 times as long as body (aa); middle lobe of mesoscutum less protuberant in lateral view (bb); if convex (bbb) then pronotal tooth wider and rather blunt (cc)	**23**
	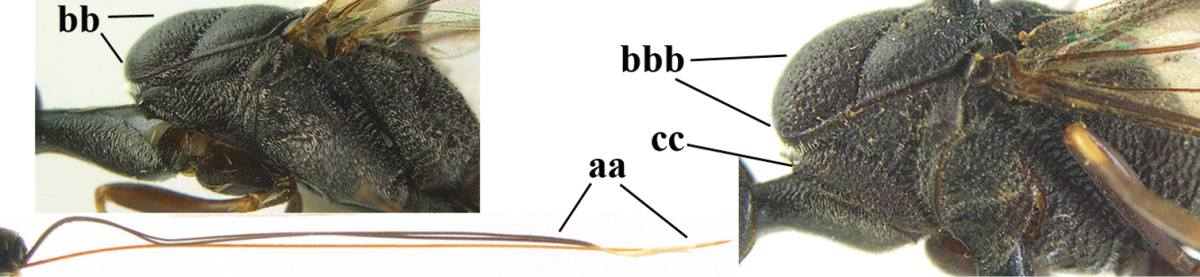	
23	Ovipositor about 0.4 times as long as body and 0.6 times as long as metasoma (a); hind coxa very slender (b); ovipositor widened apico-ventrally and more or less angularly up curved apically in dead specimens (c)	***Gasteruption angulatum* Zhao, van Achterberg & Xu, 2012**
	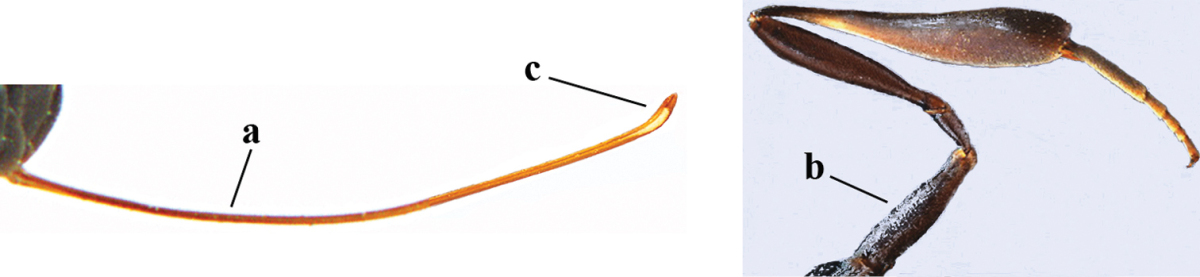	
–	Ovipositor 0.8–1.4 times as long as body and 1.2–1.9 times as long as metasoma (aa); hind coxa slightly less slender (bb); ovipositor narrow apico-ventrally and nearly straight or gradually up curved apically (cc)	**24**
	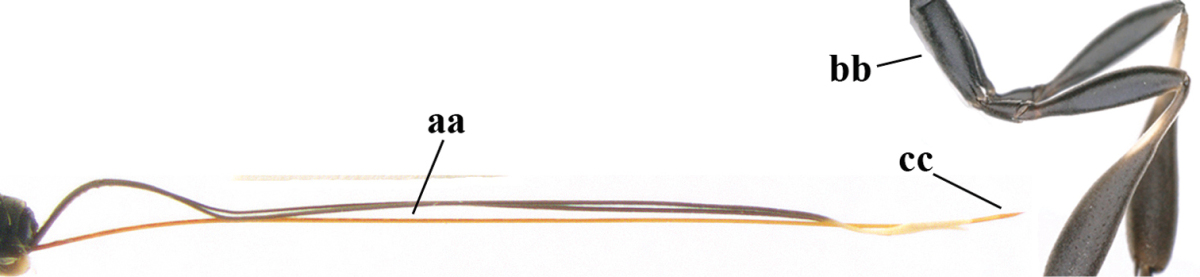	
24	Pale apical part of ovipositor sheath 3.0–3.5 times as long as hind basitarsus (a); vertex shiny and largely smooth or finely punctulate (b); fourth antennal segment 1.7–2.3 times as long as third antennal segment (c); mesoscutum more or less coarsely spaced punctate or punctate-rugose medio-posteriorly (d)	***Gasteruption tonkinense* Pasteels, 1958**
	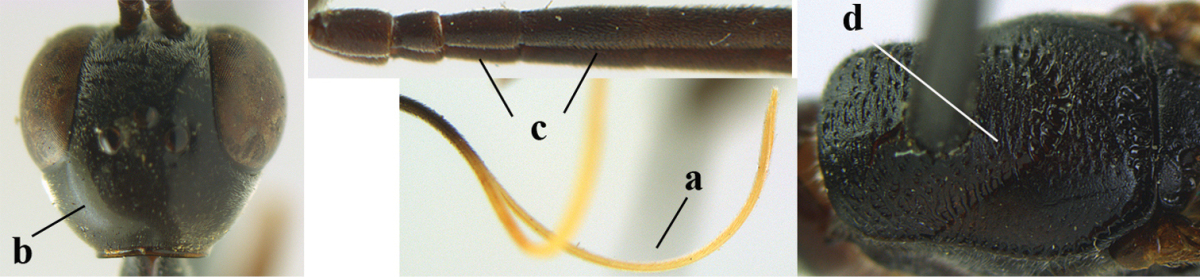	
–	Pale apical part of ovipositor sheath 0.3–3.2 times as long as hind basitarsus (aa); **if** 2.8–3.2 times (*Gasteruption japonicum*) then head dorsally with satin sheen and distinct fine sculpture (bb); fourth antennal segment 1.2–1.9 times as long as third antennal segment (cc); mesoscutum punctate, transverse rugose, punctate-rugose or partly reticulate medio-posteriorly (dd)	**25**
	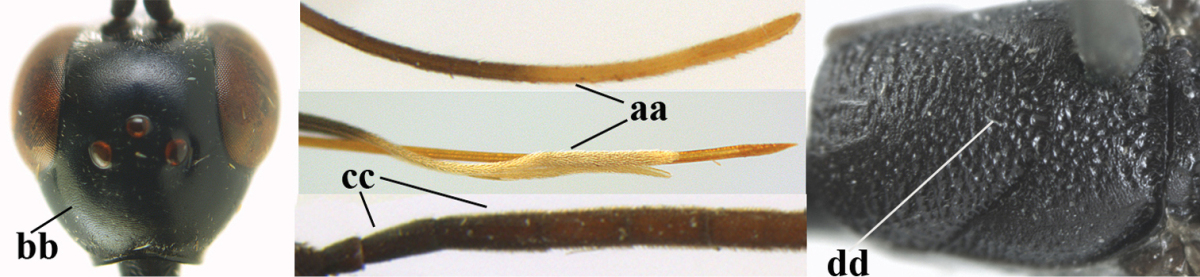	
25	Hind femur and tibia widened (a); hind basitarsus robust (b); head slightly narrowed in dorsal view (c); head slender in anterior view (d) and face narrower than clypeus (e); hind basitarsus entirely dark brown (f); [ovipositor sheath about 1.4 times as long as body and 8.5 times as long as hind tibia]	***Gasteruption huangshii* sp. n.**
	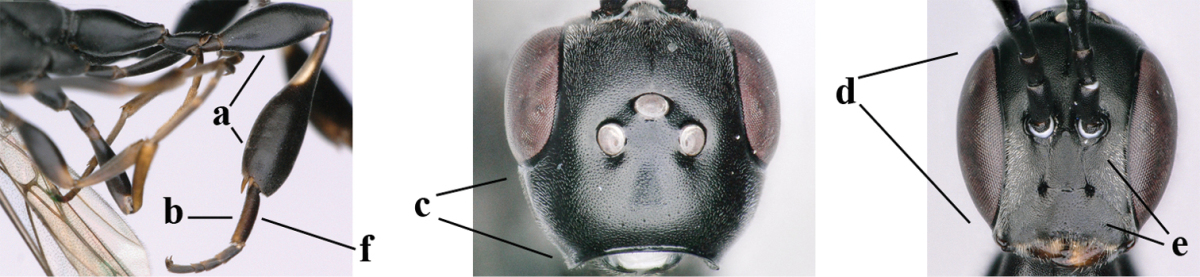	
–	Hind femur and tibia slender (aa); hind basitarsus slender (bb); head in dorsal view distinctly narrowed (cc); head in anterior view subglobular (dd), **if** slender (ddd) then face as wide as clypeus (ee); hind basitarsus often partly ivory (ff)	**26**
	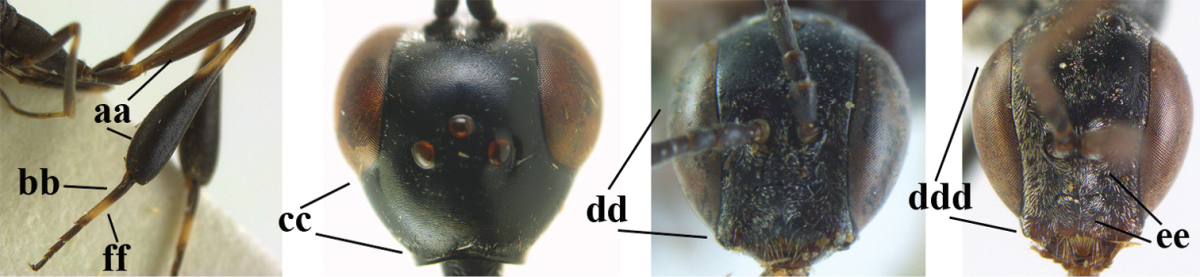	
26	Mesoscutum punctulate between rather coarse punctures (a); head slimmer in anterior view (b); [pale apical part of ovipositor sheath up to about 0.3 times as long as hind basitarsus; metasoma entirely orange or yellowish brown, at most darkened apically; ovipositor sheath 1.0–1.3 times as long as body]	**27**
	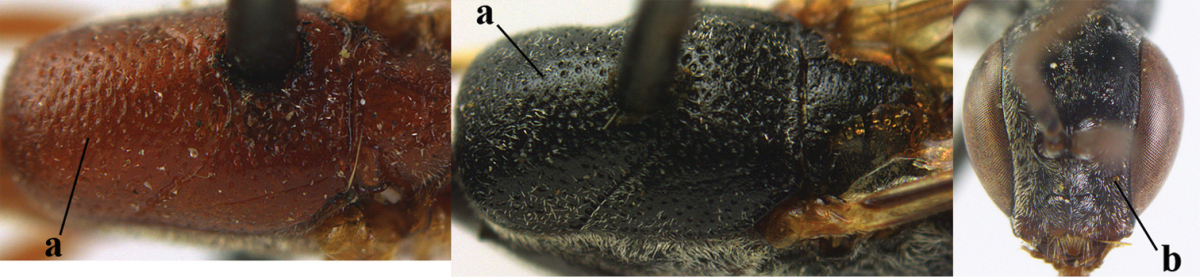	
–	Mesoscutum coriaceous between punctures (aa) or coriaceous-punctulate; head in anterior view less slender (bb)	**28**
	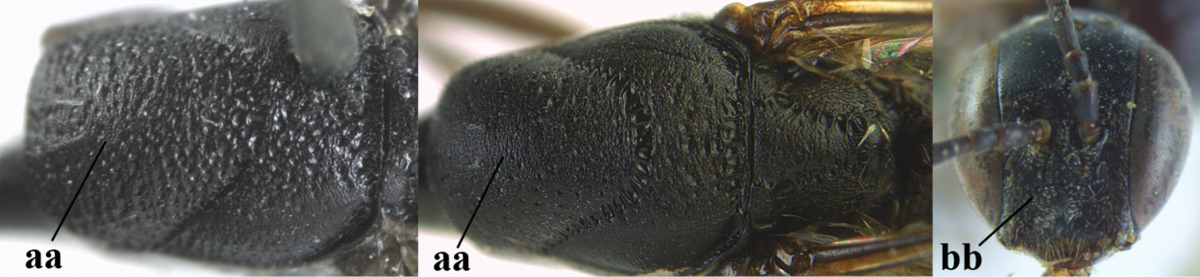	
27	Mesosoma dorsally (a), hind coxa (b) and femur (c) reddish or orange brown; hind basitarsus mainly ivory (d)	***Gasteruption argentifrons* Semenov T.-S. & Kostylev, 1928**
	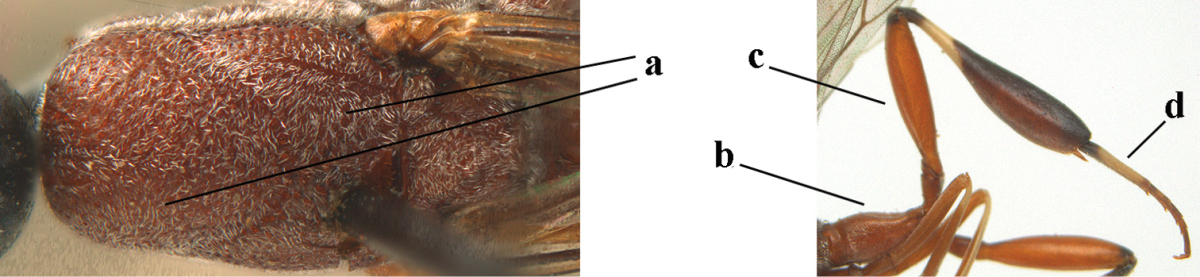	
–	Mesosoma dorsally black (aa), hind coxa (bb), hind femur (cc) and hind basitarsus (dd) dark brown	***Gasteruption dimidiatum* Semenov, 1892**
	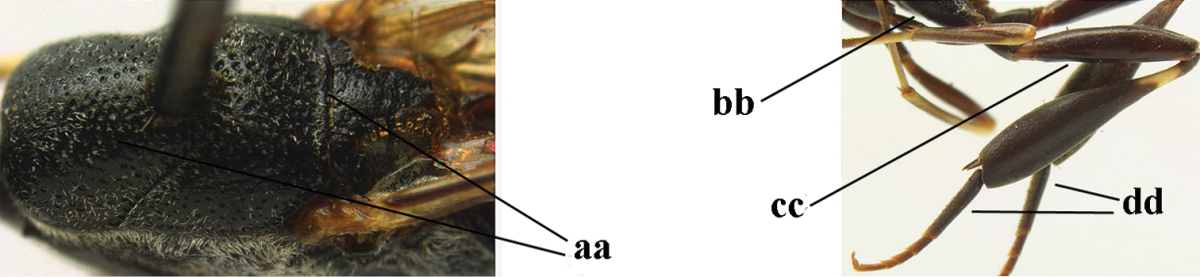	
28	Pale apical part of ovipositor sheath 0.3–1.0 times as long as hind basitarsus (a); occipital carina non-lamelliform medio-dorsally (b) **and** mesoscutum coarsely punctate or rugose (c)	***Gasteruption sinarum* Kieffer, 1911**
	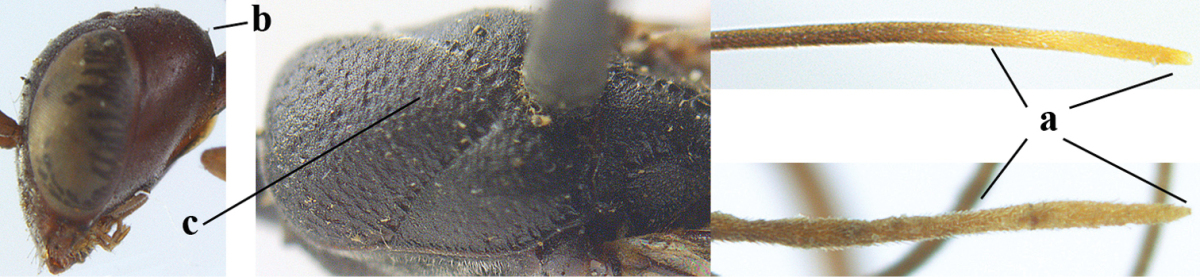	
–	Pale apical part of ovipositor sheath 1.1–3.2 times as long as hind basitarsus (aa); occipital carina narrow lamelliform medio-dorsally (bb), **if** non-lamelliform (bbb) then mesoscutum very finely coriaceous or rugulose (cc)	**29**
	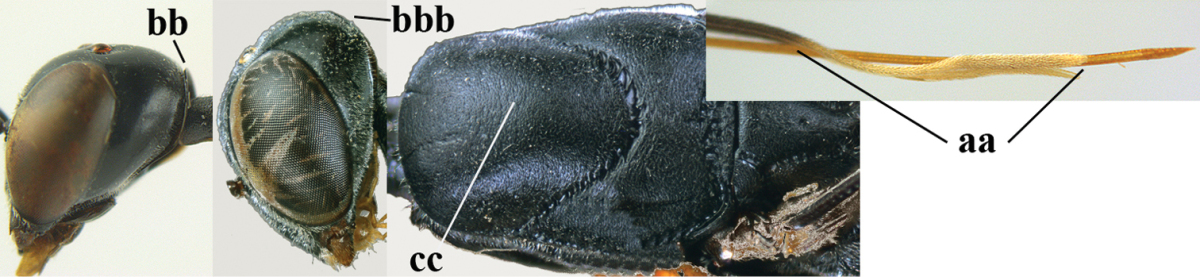	
29	Anterior half of mesoscutum normally coriaceous and with distinct punctures (a); head dorsally with satin sheen and micro-sculptured (b); head slightly narrowed in dorsal view (c)	***Gasteruption poecilothecum* Kieffer, 1911**
	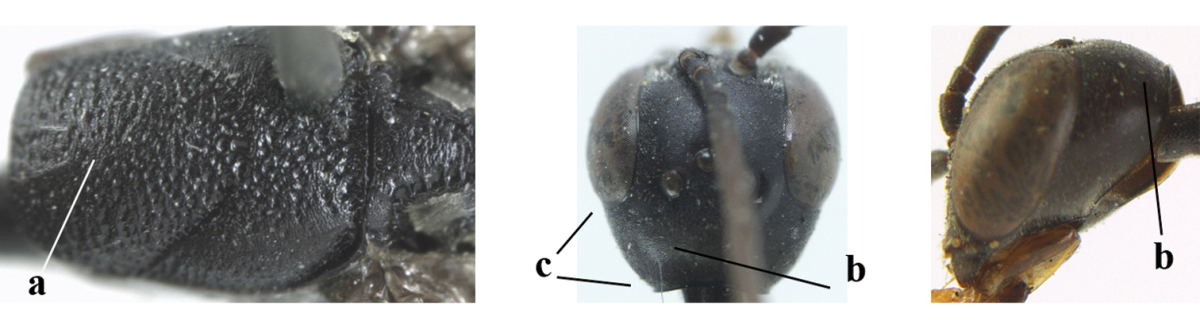	
–	Anterior half of mesoscutum very finely and densely coriaceous, without (aa) or with punctures (aaa), rarely very finely transversely rugulose; head dorsally more or less shiny and smooth (bb), at most very finely punctulate; head directly narrowed posteriorly in dorsal view (cc)	**30**
	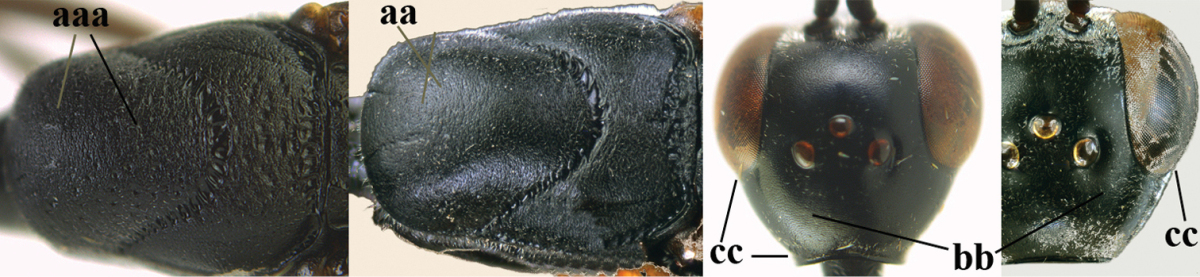	
30	Occipital carina fine and non-lamelliform dorsally (a); vertex in front of occipital carina without depression (b); head rather stout in dorsal view (c); mesoscutum at most punctulate, usually hardly or not punctate (d)	***Gasteruption sinepunctatum* Zhao, van Achterberg & Xu, 2012**
	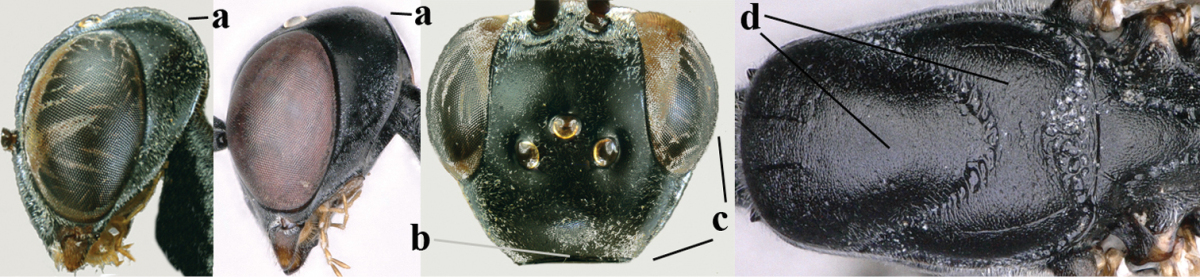	
–	Occipital carina narrow lamelliform dorsally (aa); vertex in front of occipital carina with indistinct depression (bb); head slimmer in dorsal view (cc); mesoscutum more or less punctate (dd)	**31**
	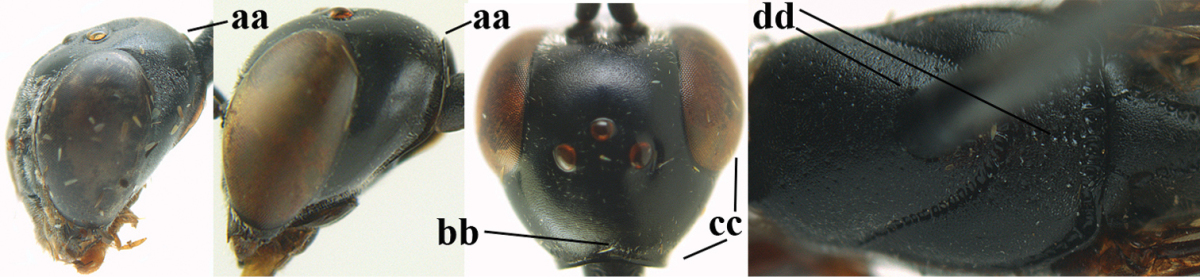	
31	Mesoscutum moderately punctate (a); hind coxa rather robust basally (b); white or ivory apical part of ovipositor sheath 1.4–2.2 times as long as hind basitarsus (cc); [hind basitarsus often partly ivory]	***Gasteruption japonicum* Cameron, 1888**
	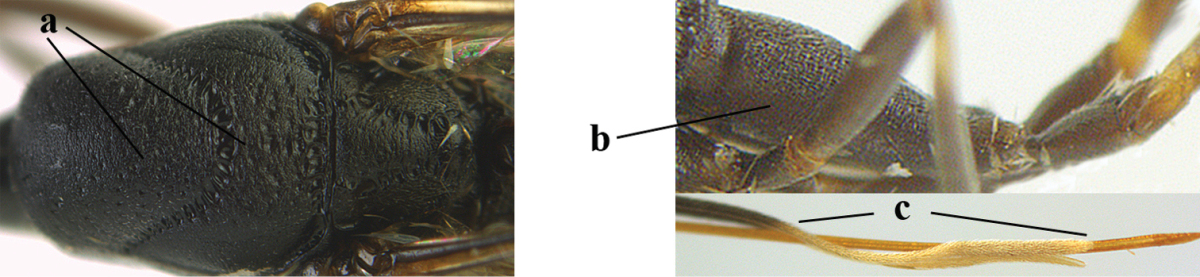	
–	Mesoscutum at most finely punctate (aa); hind coxa slimmer basally (bb); white or ivory apical part of ovipositor sheath 2.1–3.2 times as long as hind basitarsus (cc)	***Gasteruption rufescenticorne* Enderlein, 1913**
	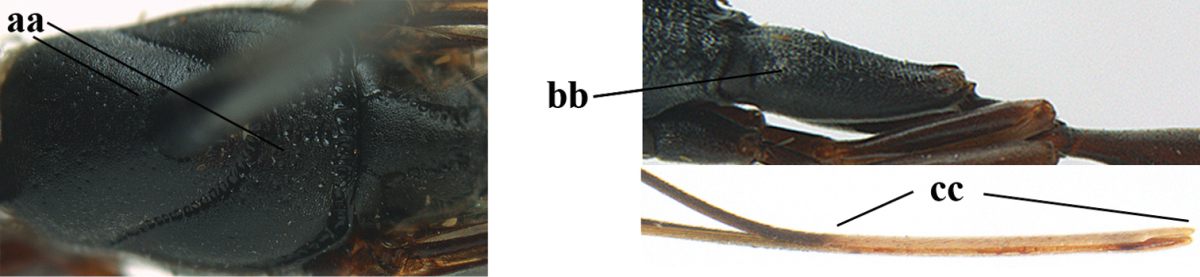	

### Males

**Table:** 

32	Vertex with distinct medio-posterior depression or a groove with two minute tubercles in front of distinctly lamelliform occipital carina (a); mesoscutum medially transversely or obliquely rugulose, in large specimens transversely rugose (b); occipital carina wide lamelliform (c)	***Gasteruption oshimense* Watanabe, 1934**
	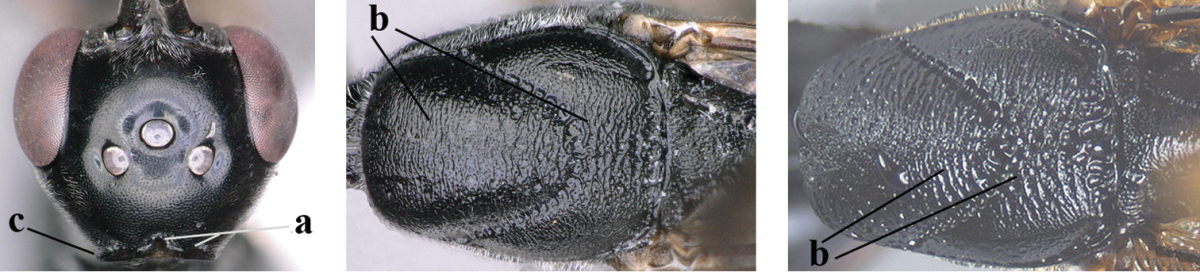	
–	Vertex flat or evenly convex in front of occipital carina (aa), **if** slightly depressed (aaa) then mesoscutum mainly punctate medially (bb) or occipital carina narrow lamelliform (cc)	**33**
	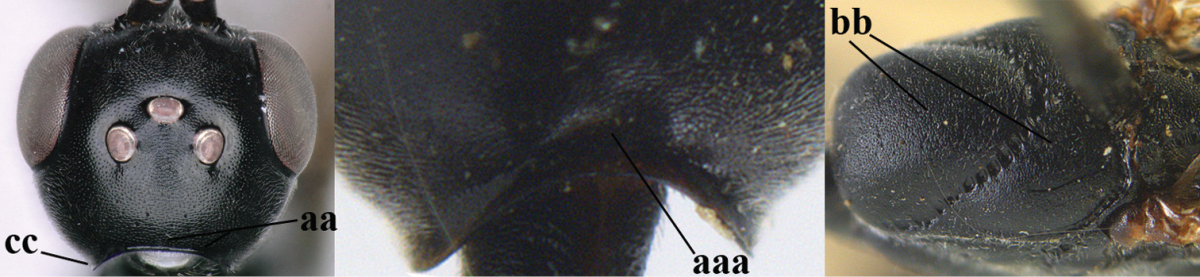	
33	Clypeus with rather large shallow depression (a); hind basitarsus rather stout (b); mesoscutum reticulate or rugose (c); [head and scapus more or less orange or reddish-brown, but sometimes entirely black]	***Gasteruption formilis* Alekseev, 1995**
	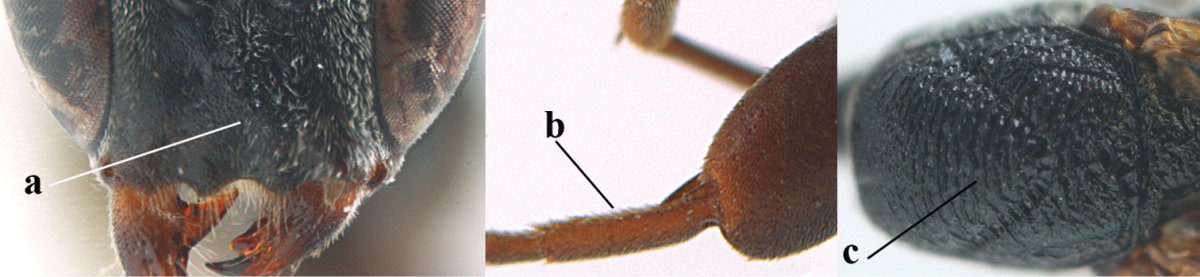	
–	Clypeus with small depression or depression obsolescent (aa); hind basitarsus often slimmer (bb) or mesoscutum coriaceous or rugulose (cc)	**34**
	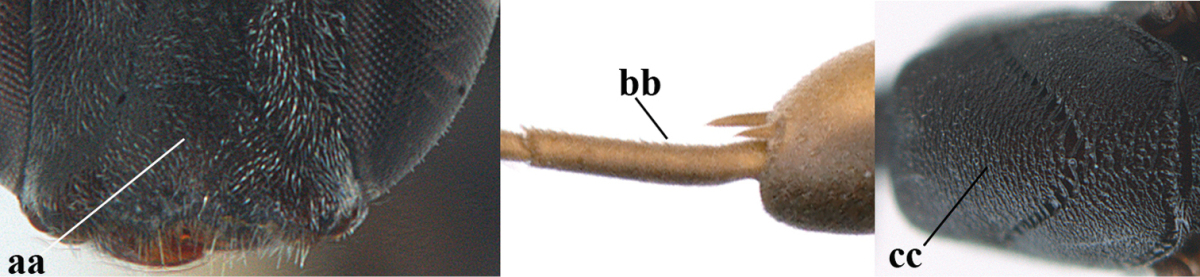	
34	Head in anterior view distinctly protruding below lower level of eyes (a), in lateral view condylar incision of malar space remains distinctly removed from eye, malar area behind indentation square and at least 0.8 times as long as second antennal segment (= pedicellus) and 0.7–0.9 times basal width of mandible (b); mesoscutum densely coriaceous and matt, similar to vertex (c)	***Gasteruption oriplanum* Kieffer, 1914**
	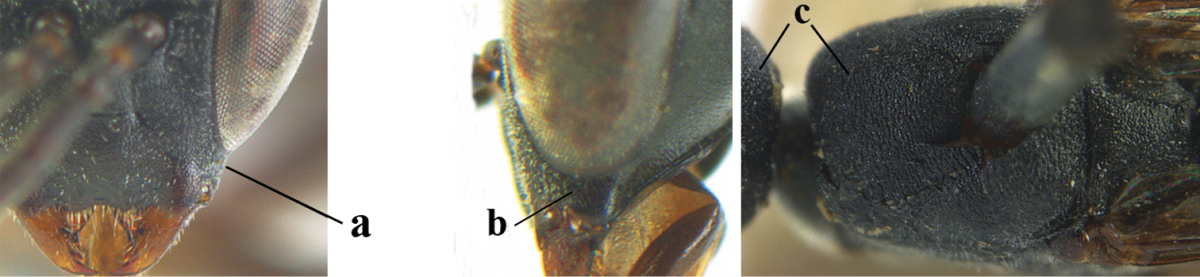	
–	Head in anterior view hardly protruding below lower level of eyes (aa), in lateral view condylar incision of malar space close to eye and malar area behind indentation transverse and 0.3–0.5 times as long as second antennal segment and 0.2–0.3 times basal width of mandible (bb); **if** malar space slightly enlarged then mesoscutum punctate or transversely rugose and rather shiny, different from vertex (cc)	**35**
	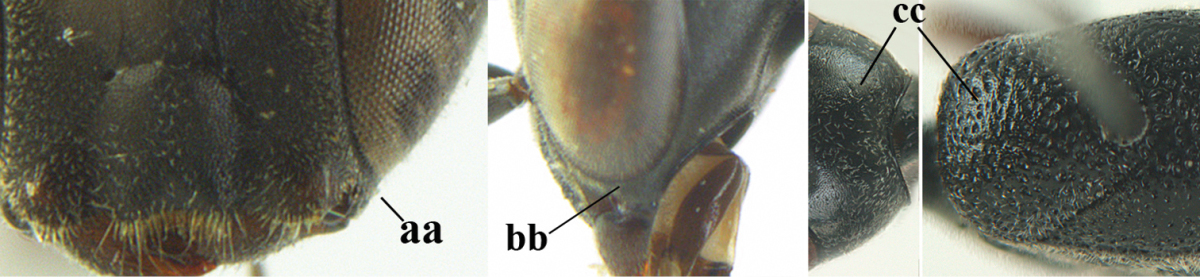	
35	Mesoscutum only coriaceous or finely rugulose medially (a), at most with a few shallow punctures; [*Gasteruption assectoides* provisionally included, ♂ unknown]	**36**
	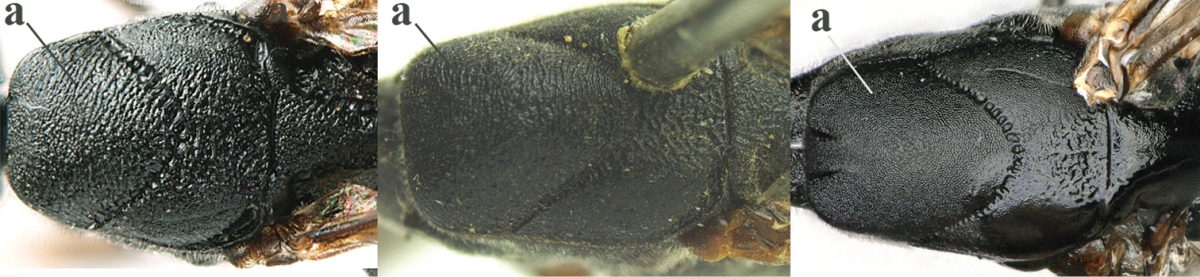	
–	Mesoscutum with several distinctly impressed punctures medially (aa; but often shallow in *Gasteruption japonicum*) or reticulate-rugose (aaa)	**47**
	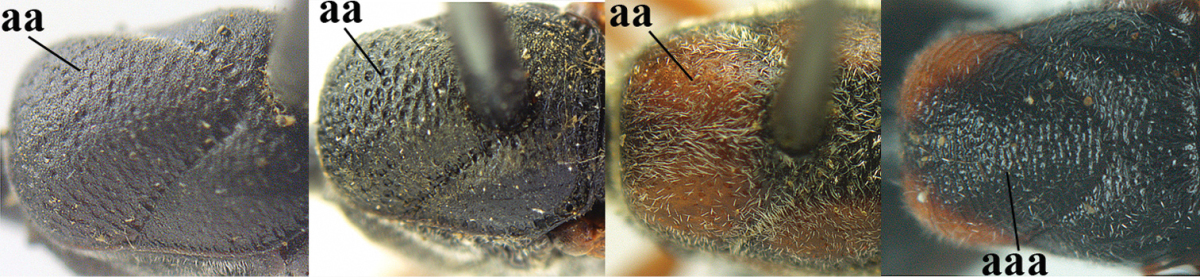	
36	Hind tibia slender, hardly to moderately inflated (a); mesoscutum often very finely and regularly sculptured (b)	**37**
	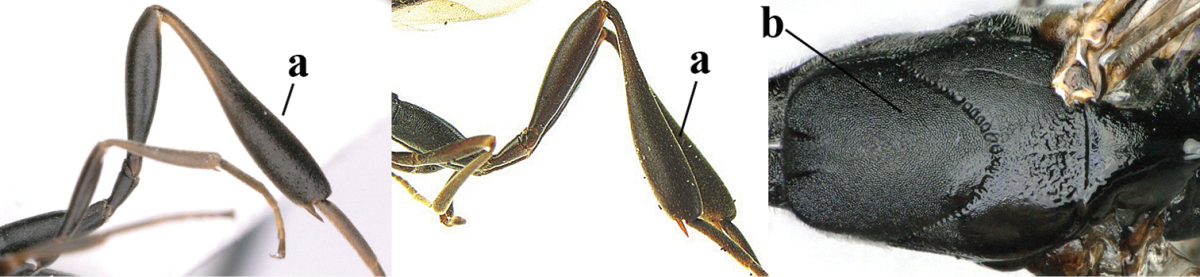	
–	Hind tibia distinctly inflated (aa); mesoscutum mainly densely coriaceous or irregularly rugulose (aa), especially near notauli (bb)	**42**
	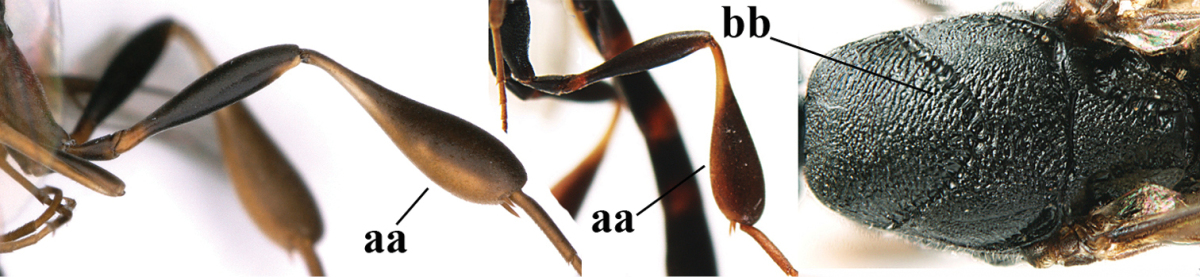	
37	Mesoscutum distinctly regularly transversely rugulose (a); hind tibial spurs yellowish and distinctly contrasting with dark hind basitarsus (b)	***Gasteruption assectoides* Zhao, van Achterberg & Xu, 2012**
	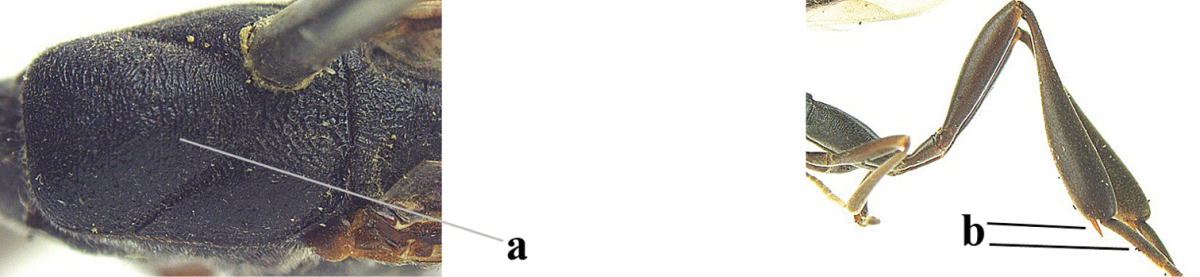	
–	Mesoscutum mainly finely coriaceous or superficially irregularly rugulose (aa); hind tibial spurs more or less brown and less contrasting with hind basitarsus (bb)	**38**
	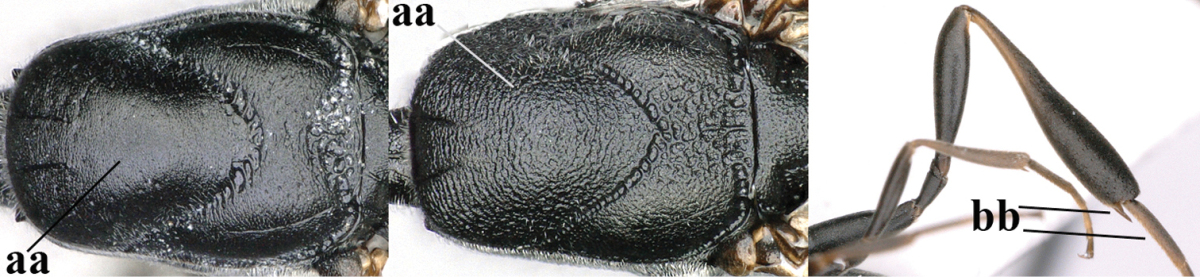	
38	Fourth antennal segment 2.5–3.5 times as long as third segment (a); face rather narrow (b)	**39**
	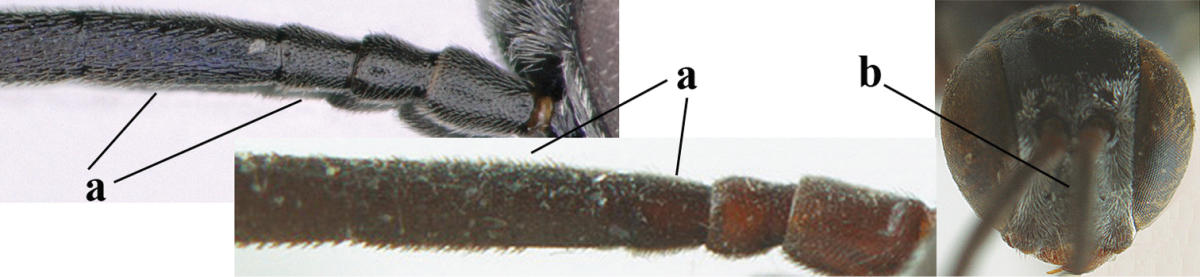	
–	Fourth antennal segment 1.4–1.7 times as long as third segment (aa; unknown of *Gasteruption pannuceum*); face wide (bb)	**40**
	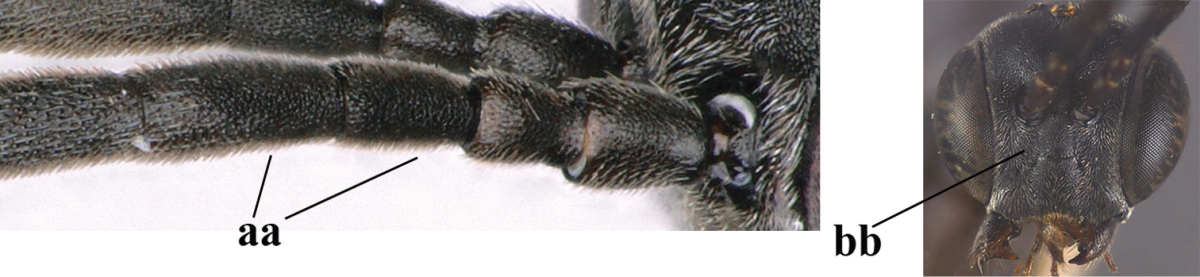	
39	Fourth antennal segment about 3.5 times as long as third segment (a); second and third antennal segments robust (b); hind femur and tibia slender (c)	***Gasteruption sinepunctatum* Zhao, van Achterberg & Xu, 2012**
	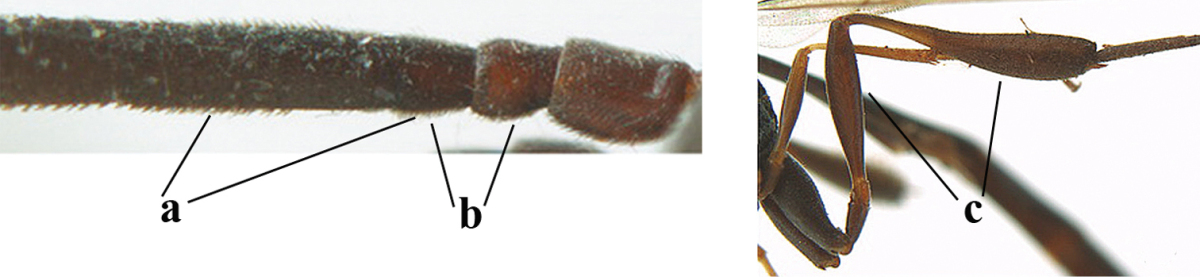	
–	Fourth antennal segment about 2.5 times as long as third segment (aa); second and third antennal segments slender (bb); hind femur and tibia rather robust (cc)	***Gasteruption huangshii* sp. n.**
	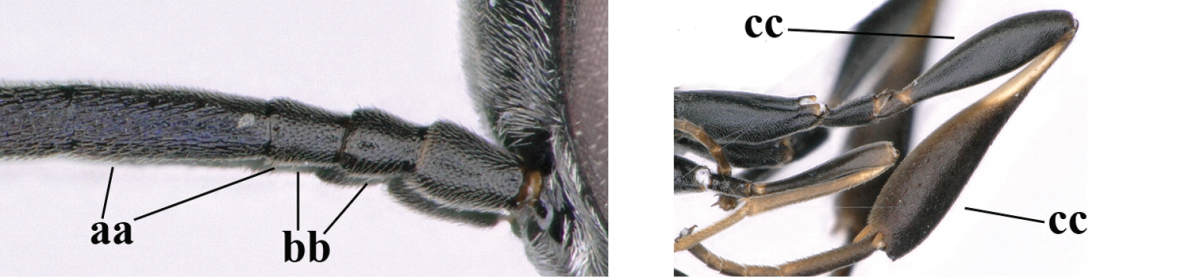	
40	Head strongly convex in lateral and anterior view (a); head long in dorsal view (b); hind coxa and tibia rather robust (c)	***Gasteruption parvicollarium* Enderlein, 1913**
	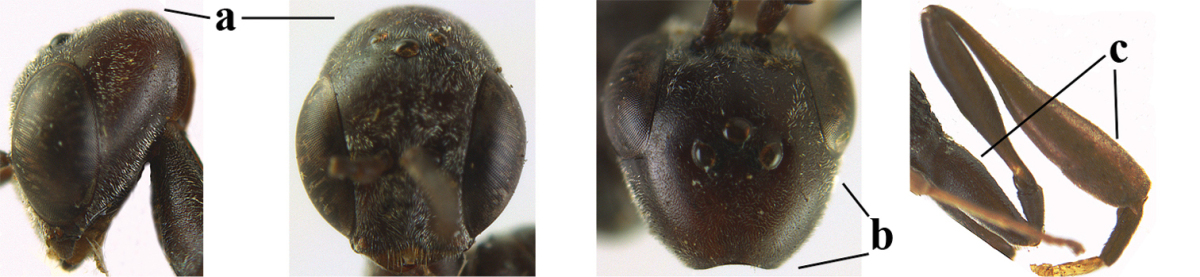	
–	Head moderately convex in lateral and anterior view (aa); head rather short in dorsal view (bb); hind coxa and tibia slender (cc)	**41**
	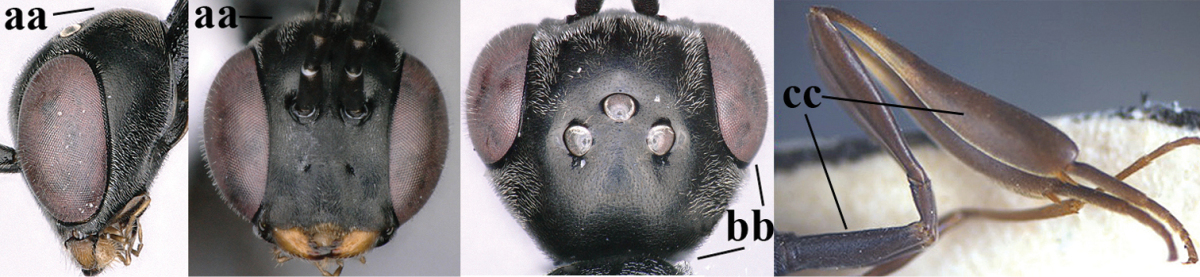	
41	Mandible largely dark brown (a); hind coxa and femur elongate (b)	***Gasteruption angulatum* Zhao, van Achterberg & Xu, 2012**
	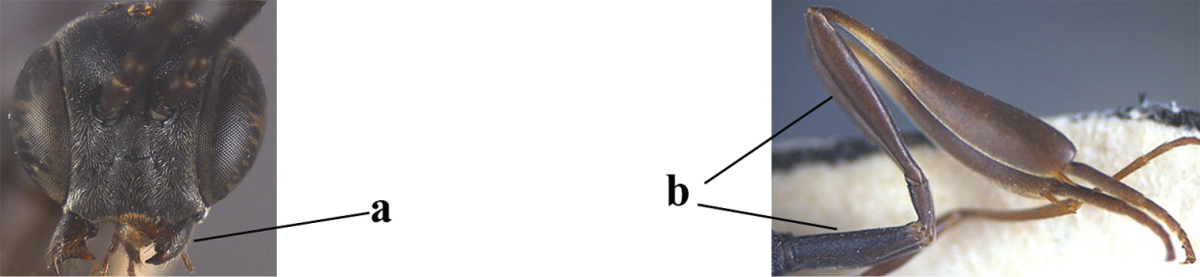	
–	Mandible yellowish brown (aa); hind coxa and femur less elongate (bb); [occipital carina non-lamelliform medio-dorsally]	***Gasteruption pannuceum* sp. n.**
	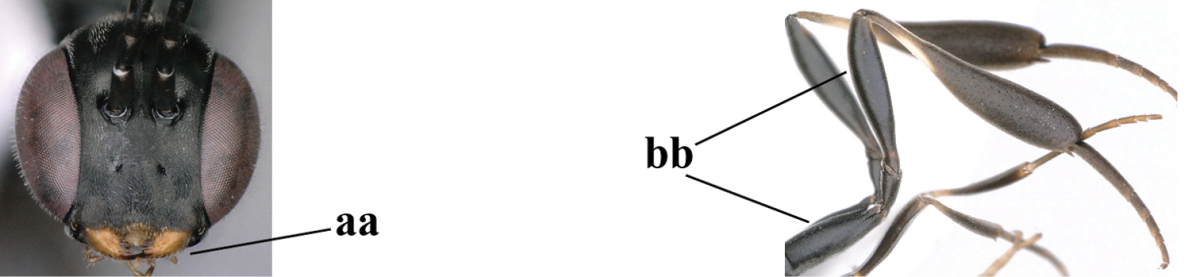	
42	Frons and vertex shiny and smooth (a); mesoscutum rugose medio-posteriorly (b) and near notauli (c)	***Gasteruption latitibia* Zhao, van Achterberg & Xu, 2012**
	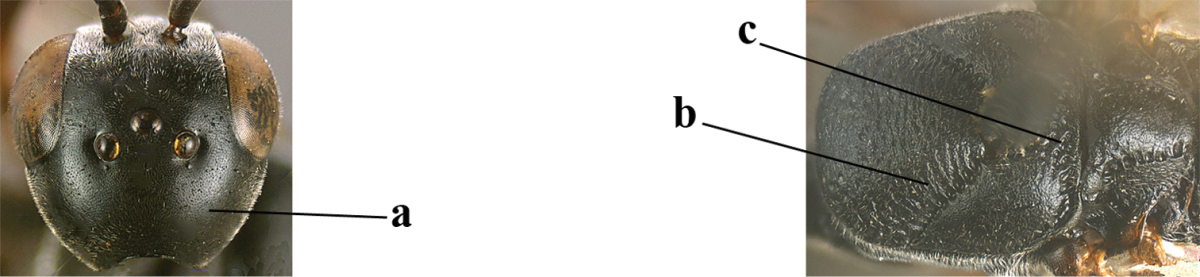	
–	Frons and vertex matt or with satin sheen, micro-sculptured (aa); mesoscutum coriaceous or rugulose near notauli (bb) and medio-posteriorly rugulose (cc) or mainly punctate	**43**
	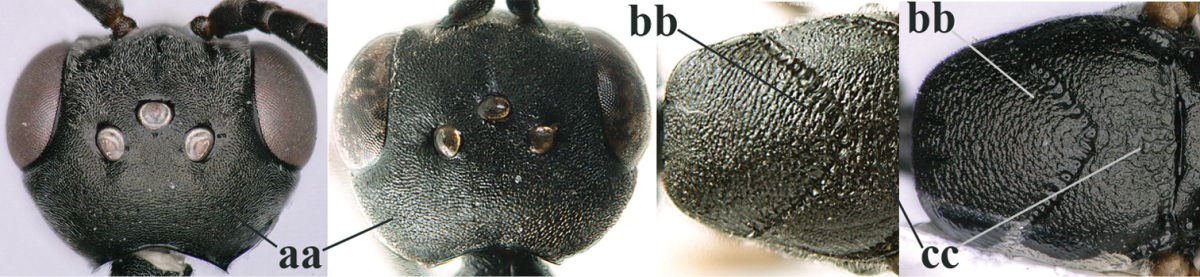	
43	Mandible pale yellowish basally (a); apex of paramere yellowish brown (b); apical metasomal sternites yellowish posteriorly (c)	***Gasteruption flavimarginatum* van Achterberg, 2014**
	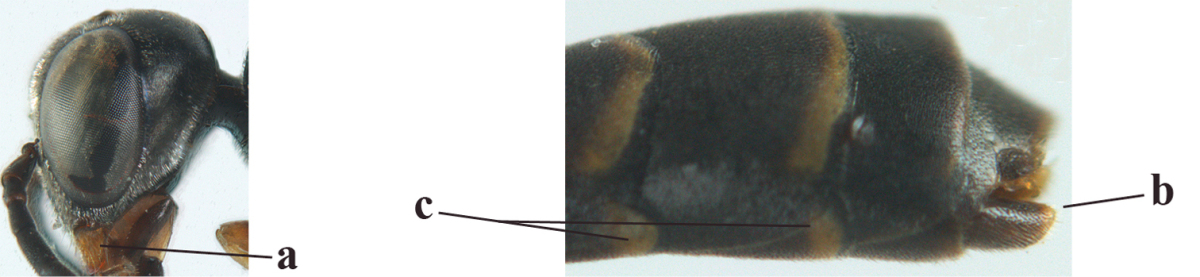	
–	Mandible brown, dark brown or black basally (aa); apex of paramere dark brown or blackish (bb); apical metasomal sternites dark brown or black posteriorly (cc)	**44**
	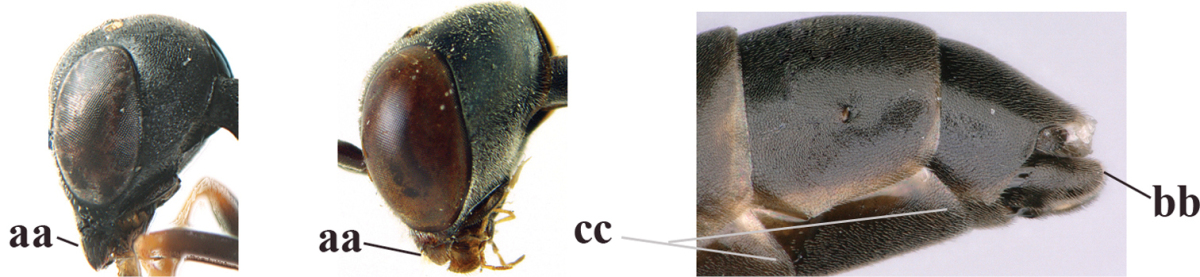	
44	Head longer and rather trapezoid in dorsal view (a); hind tibia broadly darkened basally (b)	***Gasteruption terebrelligerum* Enderlein, 1913**
	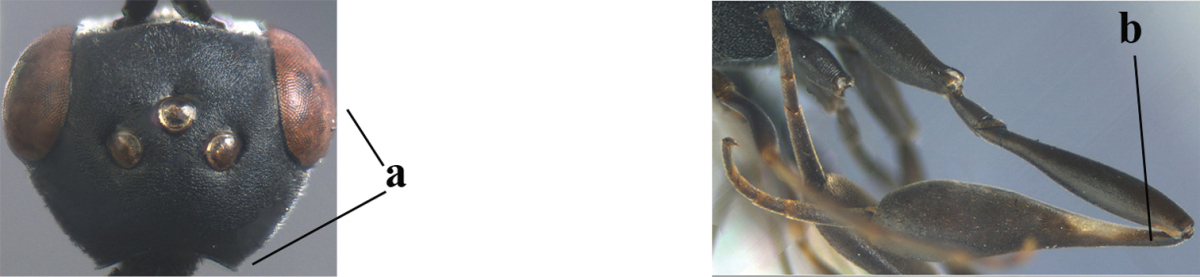	
–	Head shorter and transverse in dorsal view (aa); hind tibia yellowish or narrowly darkened basally (bb)	**45**
	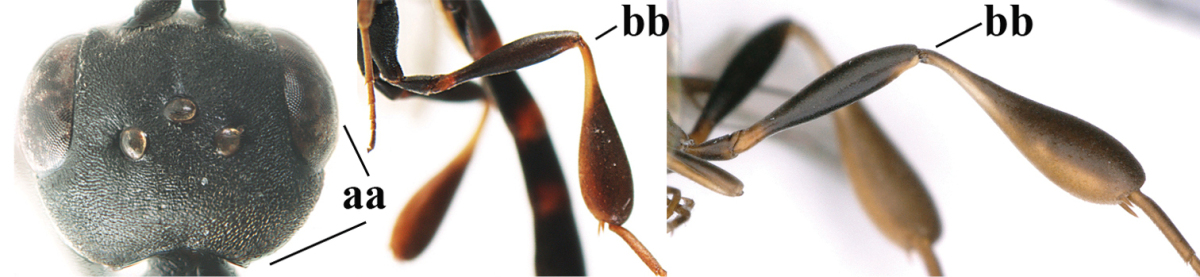	
45	Hind tibia strongly inflated (a); head less narrowed posteriorly in dorsal view (b); basal antennal segments slightly more robust (c)	**46**
	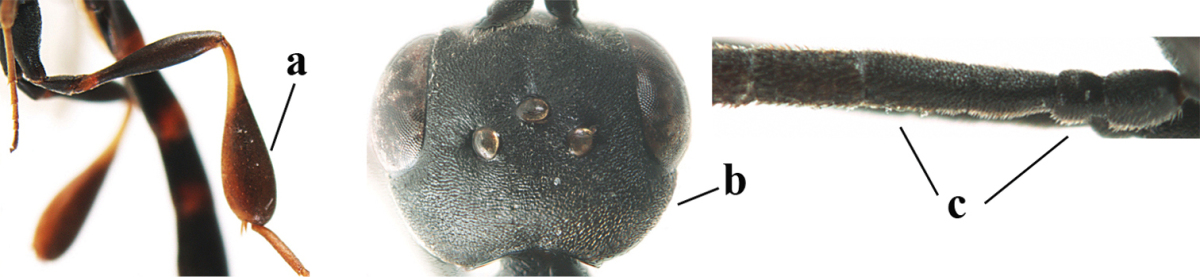	
–	Hind tibia less inflated (aa); head slightly more narrowed posteriorly in dorsal view (bb); basal antennal segments slightly slimmer (c)	***Gasteruption bicoloratum* sp. n.**
	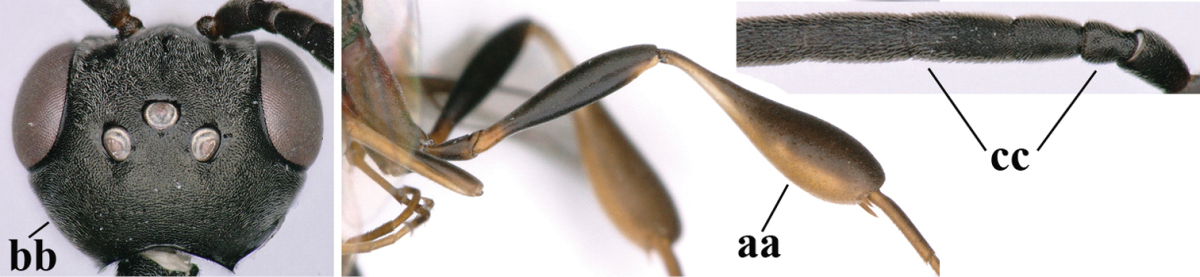	
46	Mesoscutum distinctly more coarsely sculptured than vertex (a); head directly narrowed behind eyes (b); malar space short (c)	***Gasteruption assectator* (Linnaeus, 1758)**
	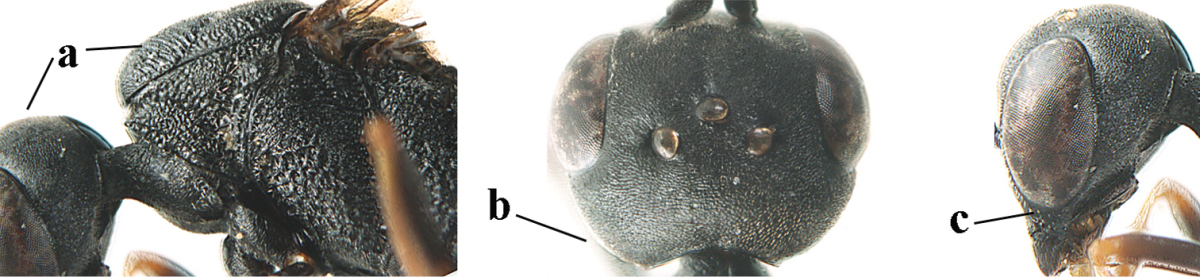	
–	Mesoscutum slightly more coarsely sculptured than vertex (aa); head less narrowed behind eyes (bb); malar space more or less enlarged (cc)	***Gasteruption boreale* (Thomson, 1883)**
	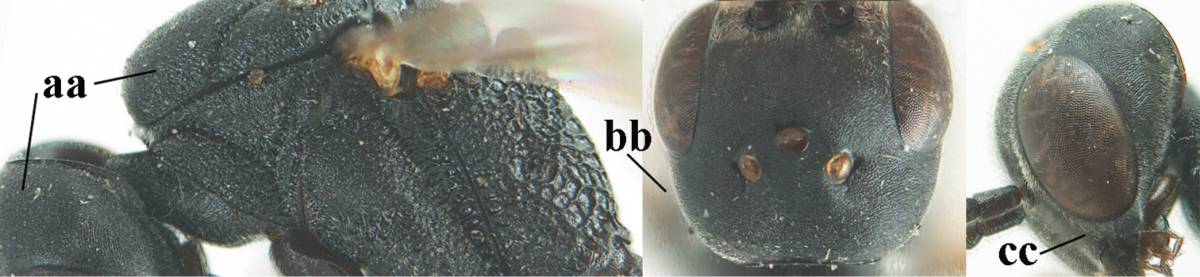	
47	Posteriorly vertex flat in lateral view and long (a); head smooth and shiny dorsally (b); head moderately narrowed posteriorly (c); mesosoma laterally often paler than dorsally (d)	***Gasteruption bimaculatum* Pasteels, 1958**
	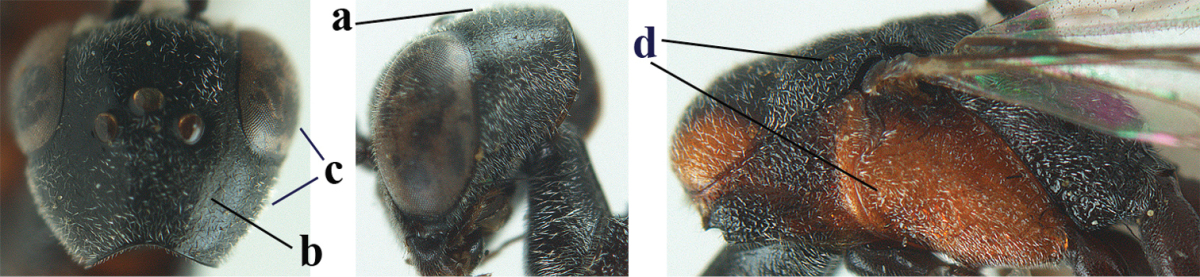	
–	Posteriorly vertex convex in lateral view and usually shorter (aa); sculpture of head dorsally variable (bb), if smooth (bbb) them more narrowed posteriorly (cc); mesosoma usually unicoloured (dd) or dorsally paler than laterally	**48**
	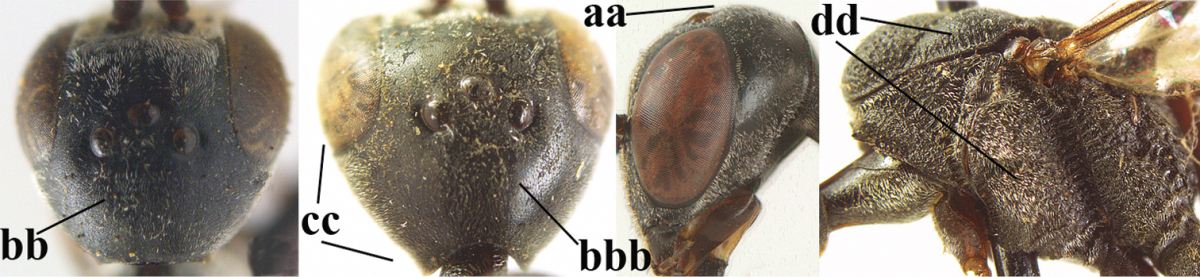	
48	Hind coxa orange brown or dark brown (a); mesoscutum rather densely setose (b); metasoma largely reddish brown (c); [mandible orange yellow or yellowish brown; mesoscutum finely or coarsely punctate and often with narrow interspaces]	**49**
	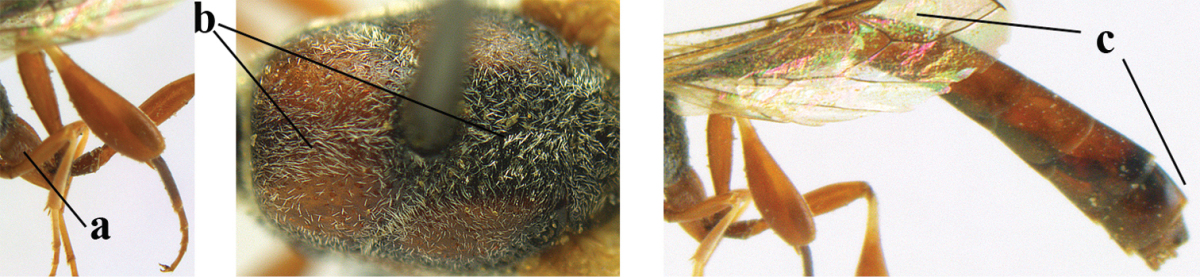	
–	Hind femur black (aa); mesoscutum less densely setose (bb); metasoma often largely dark brown or black (cc)	**51**
	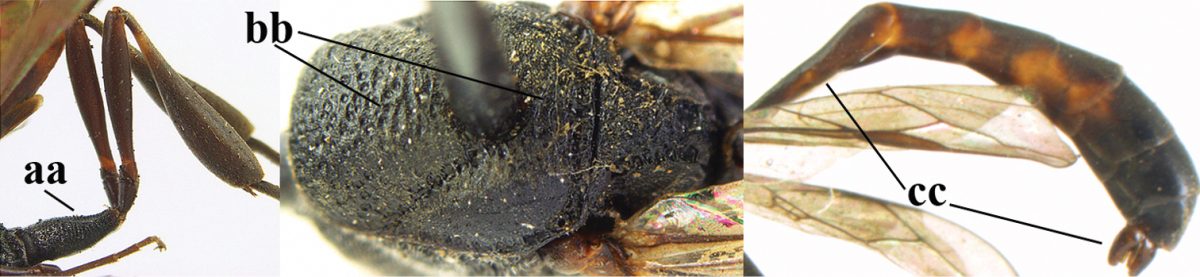	
49	Mesoscutum finely punctate, with rather wide interspaces (a); hind tibia yellowish brown or indistinctly infuscate basally (b); apical half of hind basitarsus partly dark brown and only apically ivory (c)	***Gasteruption dilutum* Semenov, 1892**
	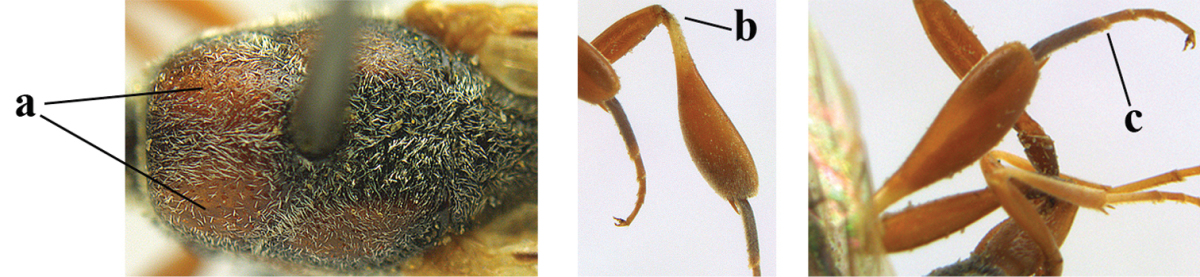	
–	Mesoscutum coarsely punctate and interspaces narrower (aa); hind tibia distinctly dark brown basally (bb); apical half of hind basitarsus mainly ivory (cc)	**50**
	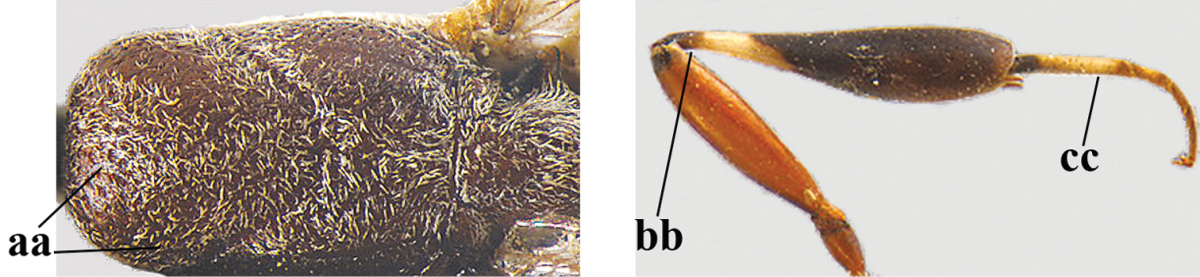	
50	Basal half of hind coxa rugose dorsally (a); outer side of hind tibia (except basally) dark brown or blackish (b); head subtruncate posteriorly in dorsal view (c)	***Gasteruption coloratum* Zhao, van Achterberg & Xu, 2012**
	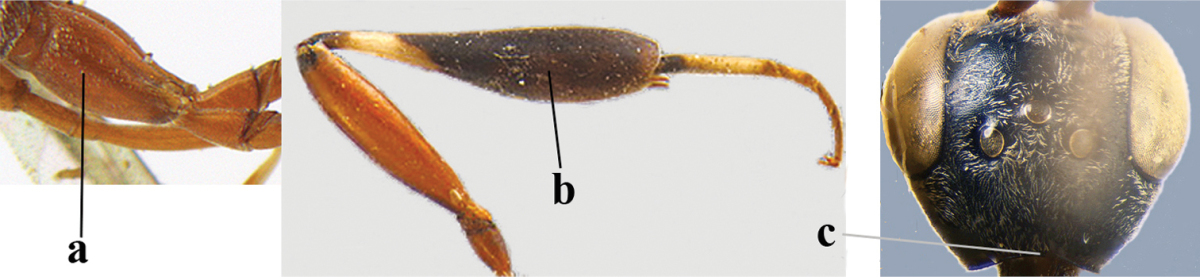	
–	Basal half of hind coxa superficially coriaceous dorsally (aa); ventral half of outer side of hind tibia orange-brown (bb); head emarginate posteriorly in dorsal view (cc)	***Gasteruption argentifrons* Semenov-T.-S. & Kostylev, 1928**
	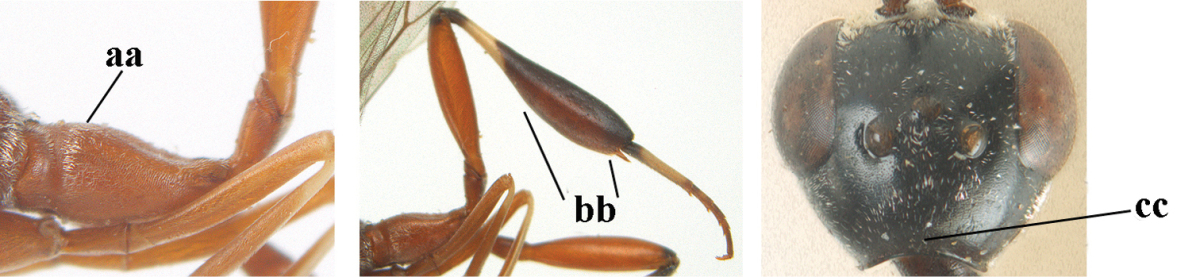	
51	Third antennal segment robust (a); ventral half of pronotal side largely superficially coriaceous to nearly smooth, only grooves crenulated (b); pronotum partly densely setose (c)	**52**
	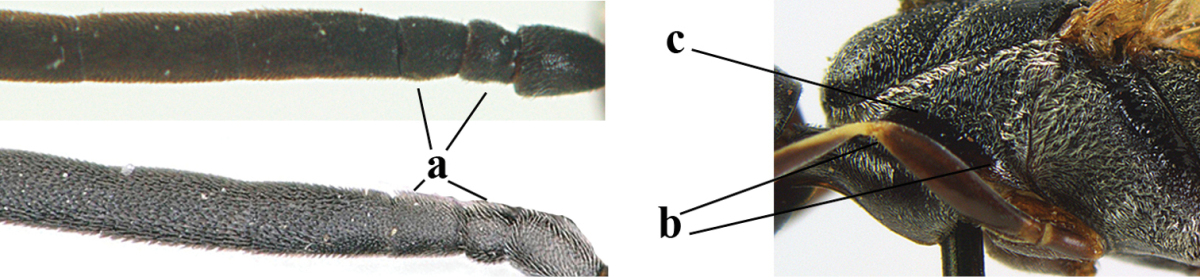	
–	Third antennal segment slender (aa); ventral half of pronotal side largely moderately reticulate-rugose, at most ventrally coriaceous (bb); pronotum sparsely setose (cc)	**53**
	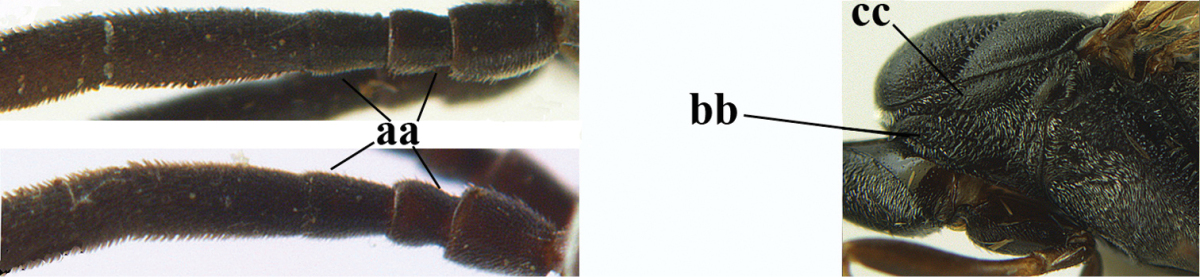	
52	Malar space narrow (a); head less emarginate medio-posteriorly (b); mesoscutum usually with less coarse sculpture (c); scutellum mainly micro-sculptured (d), at most with few large punctures	***Gasteruption shengi* sp. n.**
	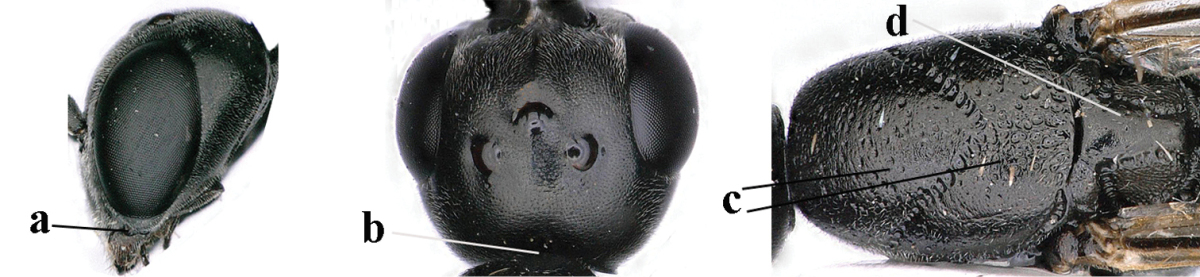	
–	Malar space slightly wider (aa); head distinctly emarginate medio-posteriorly (bb); mesoscutum with coarser sculpture (cc); scutellum coarsely punctate (dd)	***Gasteruption dimidiatum* Semenov, 1892**
	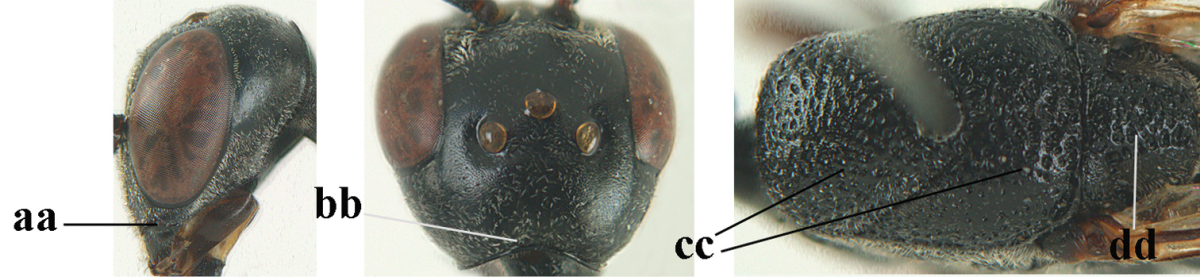	
53	Hind tibia distinctly inflated (a); head directly narrowed posteriorly in dorsal view (b); third antennal segment slender, 1.6–1.9 times as long as second segment (c); [mesoscutum distinctly “crater-like” punctate; head concave medio-posteriorly]	**54**
	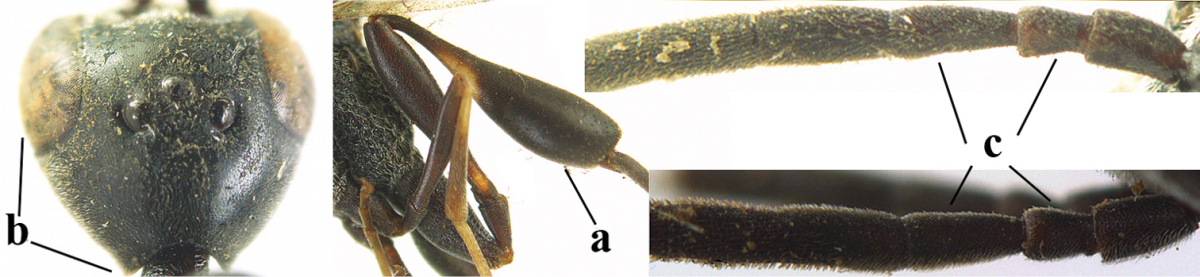	
–	Hind tibia slender (aa); head usually gradually narrowed posteriorly in dorsal view (bb); third antennal segment robust, 1.2–1.7 times as long as second segment (cc)	**55**
	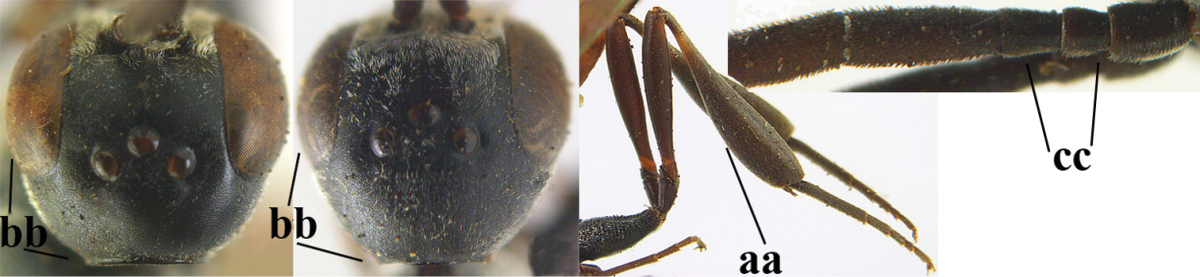	
54	Hind tibia strongly inflated (a); third antennal segment less slender (b)	***Gasteruption sinicola* (Kieffer, 1924)**
	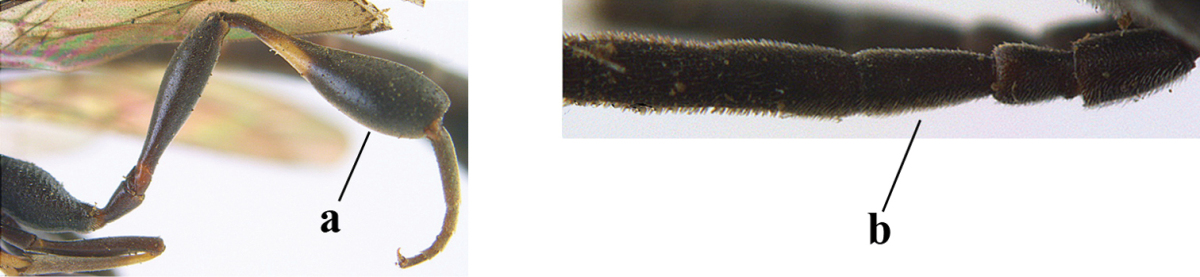	
–	Hind tibia moderately inflated (aa); third antennal segment slender (bb)	***Gasteruption formosanum* Enderlein, 1913**
	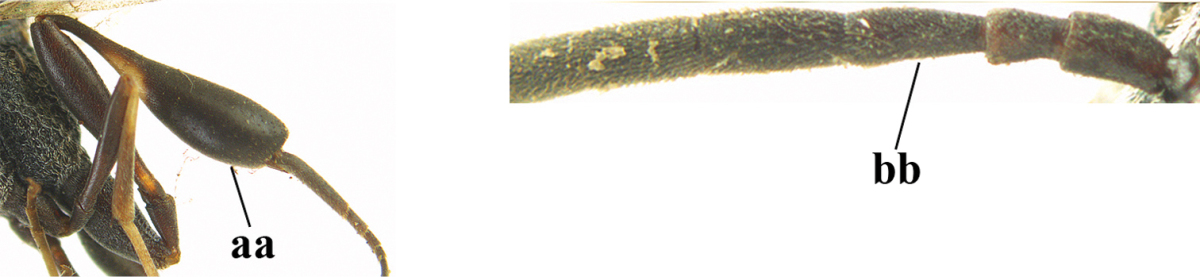	
55	Occipital carina moderately wide (a); vertex distinctly convex (b); [middle lobe of mesoscutum coarsely punctate laterally]	***Gasteruption tonkinense* Pasteels, 1958**
	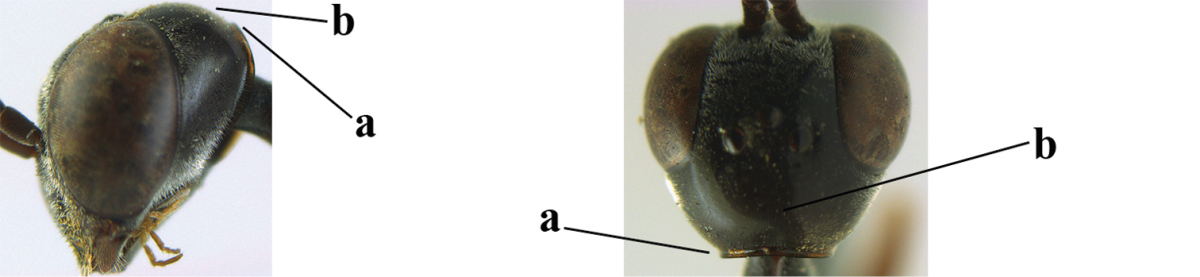	
–	Occipital carina narrow lamelliform or non-lamelliform (aa); vertex comparatively flat (bb)	**56**
	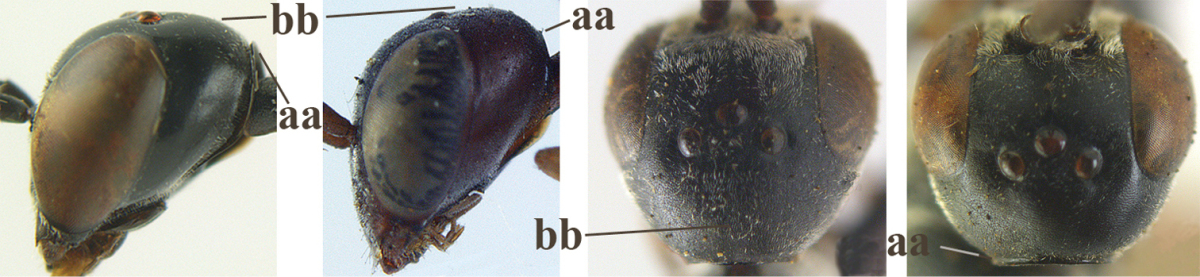	
56	Occipital carina narrow lamelliform (a) and slightly depressed in front of it medio-dorsally (b); mesoscutum less coarsely punctate (c)	***Gasteruption japonicum* Cameron, 1888**
	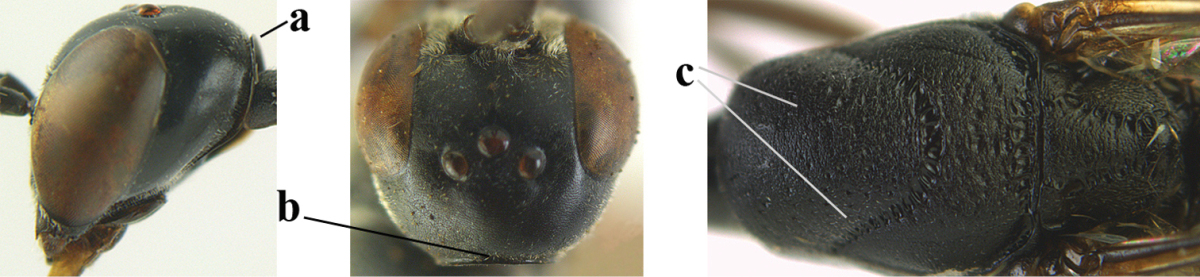	
–	Occipital carina less or non-lamelliform (aa) and flat medio-dorsally (bb); mesoscutum more or less coarsely punctate (cc)	**57**
	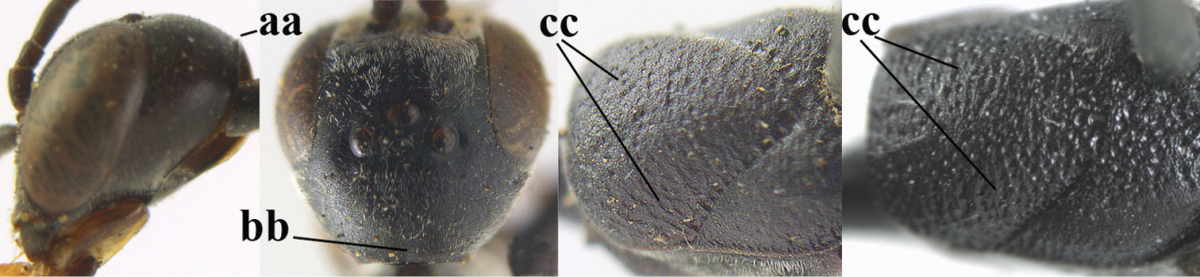	
57	Head slightly enlarged below eyes in anterior view (a); medium-sized punctures of mesoscutum not connected to form transverse elements (b); lateral lobe of mesoscutum largely finely coriaceous and rather matt, more or less punctate (c)	***Gasteruption poecilothecum* Kieffer, 1911**
	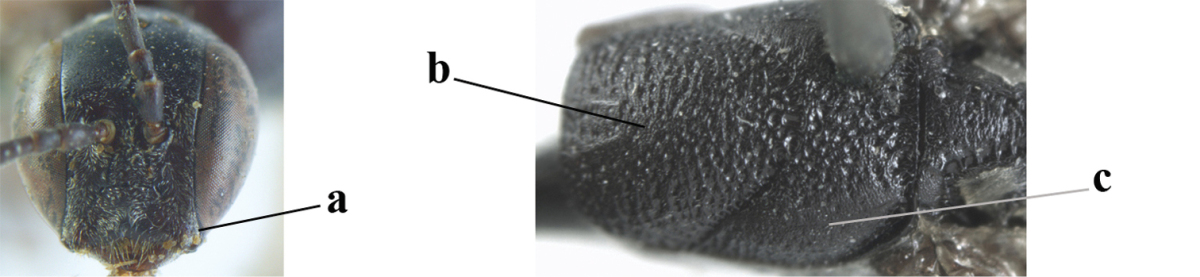	
–	Head not enlarged below eyes in anterior view (aa); large punctures of mesoscutum connected to transverse rugae or part of reticulation (bb); lateral lobe of mesoscutum more coarsely coriaceous or rugose and with satin sheen (cc)	***Gasteruption sinarum* Kieffer, 1911**
	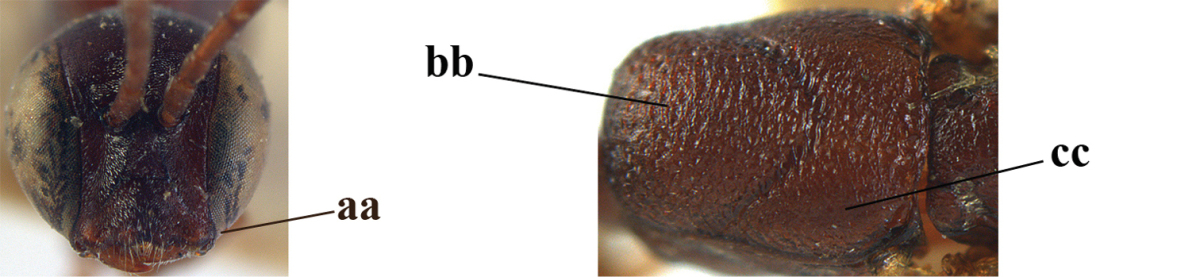	

## Systematics

### Gasteruption angulatum

Taxon classificationAnimaliaHymenopteraGasteruptiidae

Zhao, van Achterberg & Xu, 2012

Figs 1–6

Gasteruption angulatum Zhao et al., 2012: 19–22 (description).

#### Material.

1♂ (NWUX), China: Shaanxi, Mt. Qin, Baolongyu, N34°03' E108°09', 10.vi.2015, 24.v.2015, Jiangli Tan.

**Figures 1–6.**
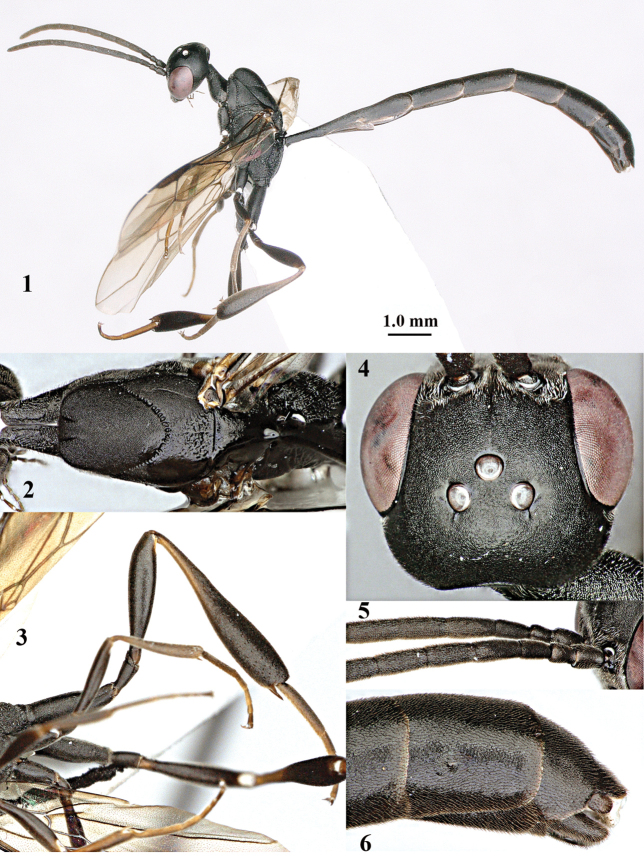
*Gasteruption angulatum* Zhao, van Achterberg & Xu, 2012, male, Shaanxi. **1** habitus lateral **2** mesosoma dorsal **3** hind leg lateral **4** head dorsal **5** basal antennal segments **6** apex of metasoma lateral.

### Gasteruption bicoloratum

Taxon classificationAnimaliaHymenopteraGasteruptiidae

Tan & van Achterberg sp. n.

http://zoobank.org/1DBA37E4-2D61-4E7E-B5B3-19D3B311D73C

Figs 7 , 8–16 , 17–22

#### Type material.

Holotype, ♀ (NWUX), “China: Shaanxi, Foping, Yueba, Qinling Mts, N33°32' E107°49', 27.vi1.vii.2015, 1095 m, Qingqing Tan”. Paratypes: 4♀3♂ (NWUX, RMNH), same data as holotype.

#### Comparative diagnosis.

Runs in Zhao et al. (2012) to *Gasteruption oriplanum* Kieffer, 1911 (but the malar space is shorter in the new species and oblique in anterior view, not subparallel-sided below eyes in anterior view as in *Gasteruption oriplanum* and the mandibles are paler) or to *Gasteruption assectator* (Linnaeus, 1758). The pale fifth sternite of the female, the strongly narrowed head in dorsal view, the shorter ovipositor sheath (about 0.6 times hind tibia vs 0.9–1.3 times in *Gasteruption assectator*) and the yellowish mandible separate it from *Gasteruption assectator*. The new species is close to *Gasteruption flavimarginatum* van Achterberg, 2014, but it has a slightly longer malar space (short in *Gasteruption flavimarginatum*), the hind basitarsus slender and dorsally dark brown (rather robust and at least partly ivory dorsally) and the mesoscutum finely sculptured (coarser sculptured). The male differs by having the apex of the paramere dark brown, which is yellowish brown in *Gasteruption flavimarginatum*.

#### Description.

Holotype, female, length of body 9.9 mm, of fore wing 4.9 mm.

*Head*. Vertex and frons with satin sheen, finely coriaceous, moderately convex and without a depression medio-posteriorly; head directly contracted behind eyes in dorsal view and temples nearly straight (Fig. 14); temple 0.9 times as long as eye in dorsal view; fourth antennal segment 1.2 times as long as third segment and 0.7 times as long as second and third segments combined, fifth antennal segment as long as third segment, third antennal segment 1.6 times as long as second segment (Fig. 8); occipital carina narrow and non-lamelliform medio-dorsally (Fig. 8); OOL 1.3 times as long as diameter of posterior ocellus; face wide, 2.8 times as broad as high, 2.4 times as wide as eye in frontal view (Fig. 13); malar space somewhat protruding below lower level of eyes (Fig. 13), its minimum width 0.4 times as long as second antennal segment and 0.35 times basal width of mandible and area behind incision nearly square (Fig. 8); clypeus only medio-ventrally shallowly depressed (Fig. 13); eye with numerous short setae.

**Figure 7.**
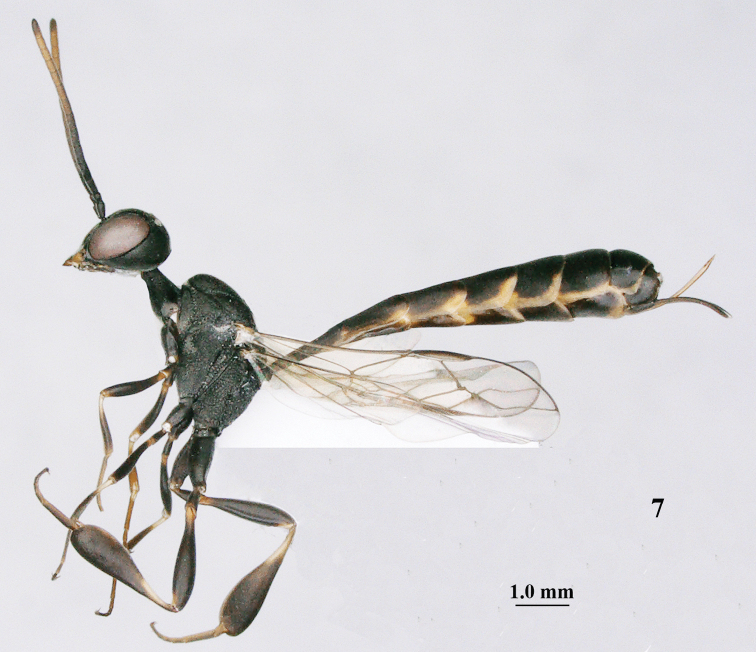
*Gasteruption bicoloratum* Tan & van Achterberg, sp. n., female, holotype, habitus lateral.

**Figures 8–16.**
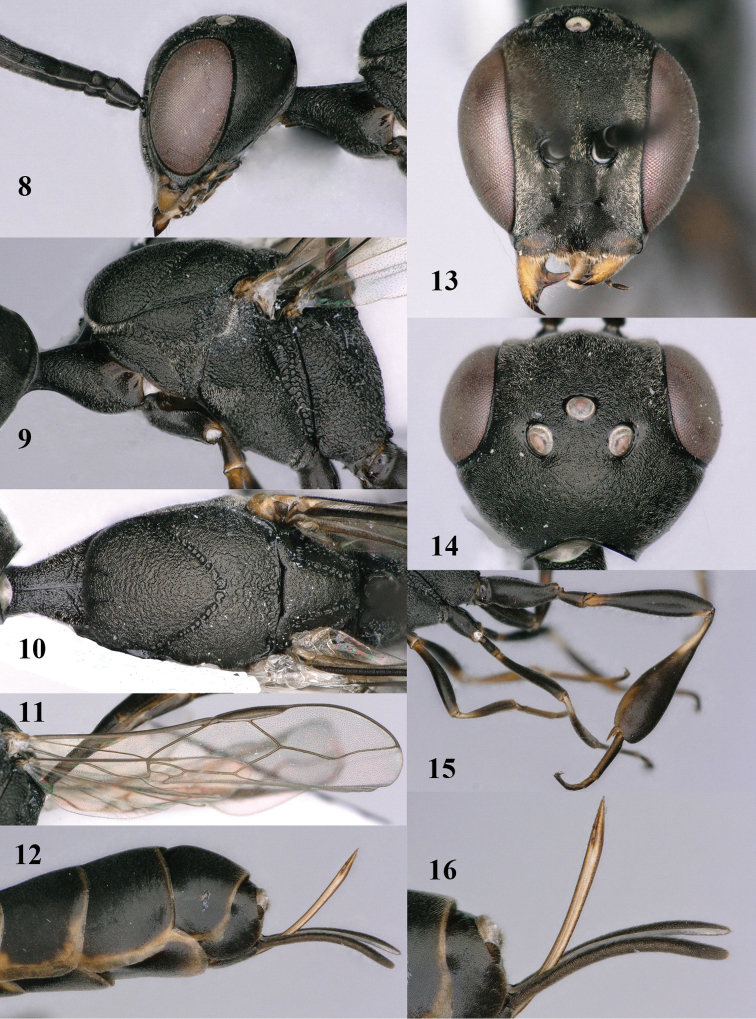
*Gasteruption bicoloratum* Tan & van Achterberg, sp. n., female, holotype. **8** head lateral **9** mesosoma lateral **10** mesosoma dorsal **11** fore wing **12** apex of metasoma lateral **13** head anterior **14** head dorsal **15** hind leg **16** ovipositor and ovipositor sheath lateral.

*Mesosoma*. Length of mesosoma 1.6 times its height; propleuron robust and 0.8 times as long as mesoscutum in front of tegulae; pronotal side entirely coriaceous except for crenulated grooves and sparsely setose, without acute tooth antero-ventrally (Figs 8–10); antesternal carina narrow and hardly lamelliform; mesosternal sulcus wide and deep; mesoscutum and scutellum rather matt and superficially rugulose-coriaceous (Fig. 10); propodeum reticulate-rugose and without median carina.

*Wings*. First discal cell parallel-sided and with outer posterior corner rounded, and with vein 3-CU1 near its apical third (Fig. 11).

*Legs*. Hind coxa finely granulate-coriaceous; length of hind femur, tibia and basitarsus 3.9, 3.6 and 4.6 times their width, respectively; hind tibia strongly inflated (Fig. 15); middle tarsus 1.1 times as long as middle tibia; middle femur subparallel-sided and slimmer than fore femur.

*Metasoma*. Ovipositor sheath 1.2 mm, 0.1 times as long as body, 0.2 times as long as metasoma and 0.6 times as long as hind tibia; ovipositor sheath with dense cover of fine brownish and adpressed setae, its apical half slender; hypopygium shallowly emarginate medio-posteriorly.

*Colour*. Black; apical half of antenna largely brown ventrally; mandible pale brownish yellow (except narrow dark borders); clypeus latero-ventrally and humeral plate dark brown; tegulum, second-seventh metasomal tergites narrowly apically and widely laterally, sixth sternite widely apically and other sternites narrowly, trochantelli, hind femur apico-ventrally and hind tibial spurs yellowish brown; fore and middle tibiae basally and hind tibia baso-ventrally widely ivory; remainder of legs, veins and pterostigma dark brown; wing membrane subhyaline.

*Male*. Similar to female (including fine sculpture of mesoscutum: Fig. 18); third antennal segment 1.3–1.6 times as long as second segment; fourth antennal segment 1.3–1.5 times as long as third segment and 0.8 times as long as second and third segments combined, fifth antennal segment 1.3–1.4 times as long as third segment (Fig. 21); apical sternite entirely dark brown; paramere densely whitish setose and its apex dark brown (Fig. 22).

**Figures 17–22.**
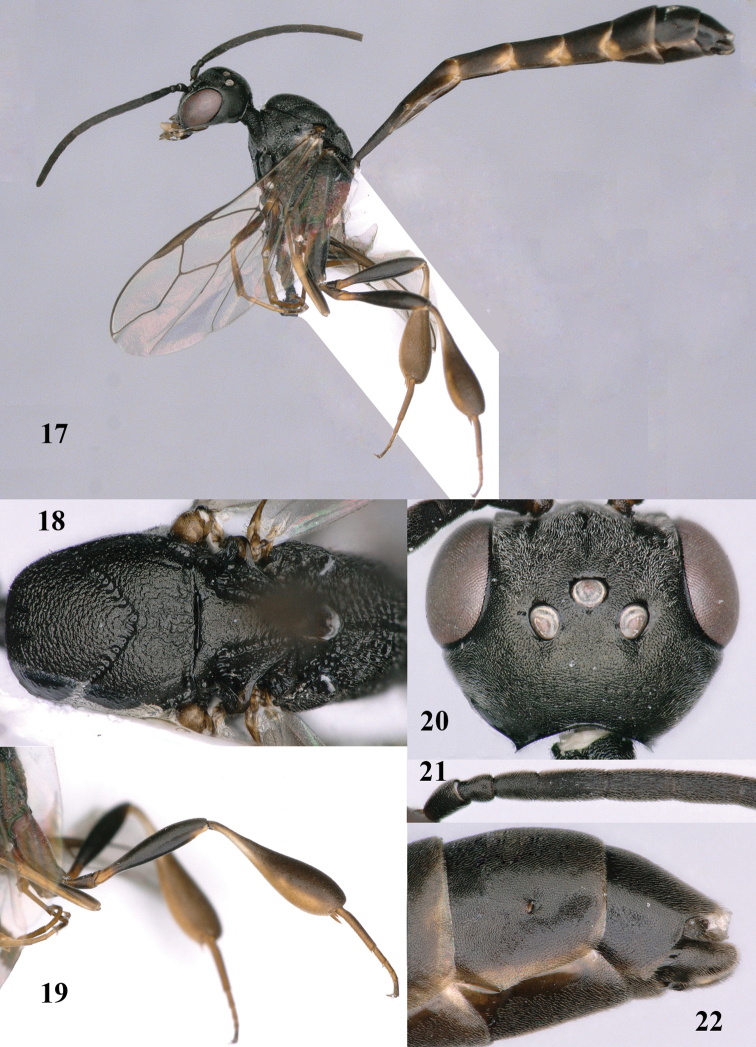
*Gasteruption bicoloratum* Tan & van Achterberg, sp. n., male, paratype. **17** habitus lateral **18** mesosoma dorsal **19** mesonotum dorsal **19** hind leg **20** head dorsal **21** basal antennal segments lateral **22** apex of metasoma lateral.

*Variation*. Body length of ♀ 8.7–10.3 mm, of ♂ 8.2–9.9 mm; length of ovipositor sheath 0.6–0.7 times hind tibia; minimum width of malar space 0.3–0.4 times as long as second antennal segment; tibiae and tarsi more or less yellowish brown ventrally; apical antennal segment more or less obliquely depressed.

#### Distribution.

China (Shaanxi). Montane: 1095 m.

#### Biology.

Unknown. Collected JuneAugust.

#### Etymology.

Named after the bicoloured hind tibia in both sexes (“bi” is Latin for “two”).

### Gasteruption boreale

Taxon classificationAnimaliaHymenopteraGasteruptiidae

(Thomson, 1883)

Figs 23 , 24–32

Foenus borealis Thomson, 1883: 849; Hedicke 1939: 7; Hedqvist 1973: 181, 182 (invalid lectotype designation); Wall 1994: 148. Synonymized with Gasteruption assectator (Linnaeus) by Schletterer (1889) and with Gasteruption minutum (Tournier) by van Achterberg and Talebi (2014).
Gasteruption boreale ; Schletterer, 1885: 303; Johansson and van Achterberg (submitted; references and synonymy).
Trichofoenus breviterebrae Watanabe, 1934: 285; Hedicke 1939: 45. Synonymized with Gasteruption assectator (Linnaeus) by Pagliano and Scaramozzino (2000) and with Gasteruption boreale (Thomson) by Johansson and van Achterberg (submitted).

#### Type material.

Holotype of *Gasteruption breviterebrae*, ♀ (ECHU), “[Russia,] Saghalien [= Sakhalin Oblast], K. Tamanuki/ Konuma, 23.v.1931”, “Holotype *Trichofoenus breviterebrae* Watanabe, 1934, det. Konishi”. Paratypes: 1 ♂ (ECHU), “[Russia,] Saghalien, K. Tamanuki/ Nagahama, 28.vii.1927”, “Paratype (Allotype) *Trichofoenus breviterebrae* Watanabe, 1934”.

#### Additional material.

**China** (Heilongjiang, ZJUH); **Russia** (Sakhalin).

#### Diagnosis.

**Figure 23.**
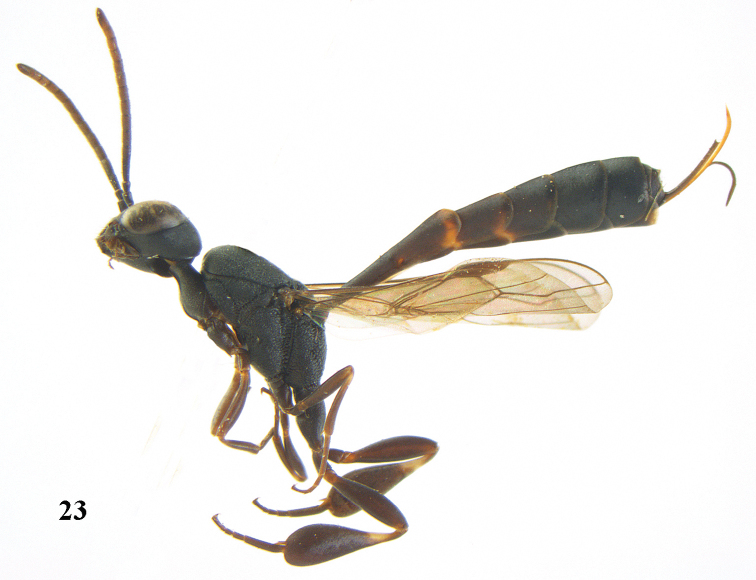
*Gasteruption boreale* (Thomson), female, holotype of *Gasteruption breviterebrae* (Watanabe), habitus lateral.

**Figures 24–32.**
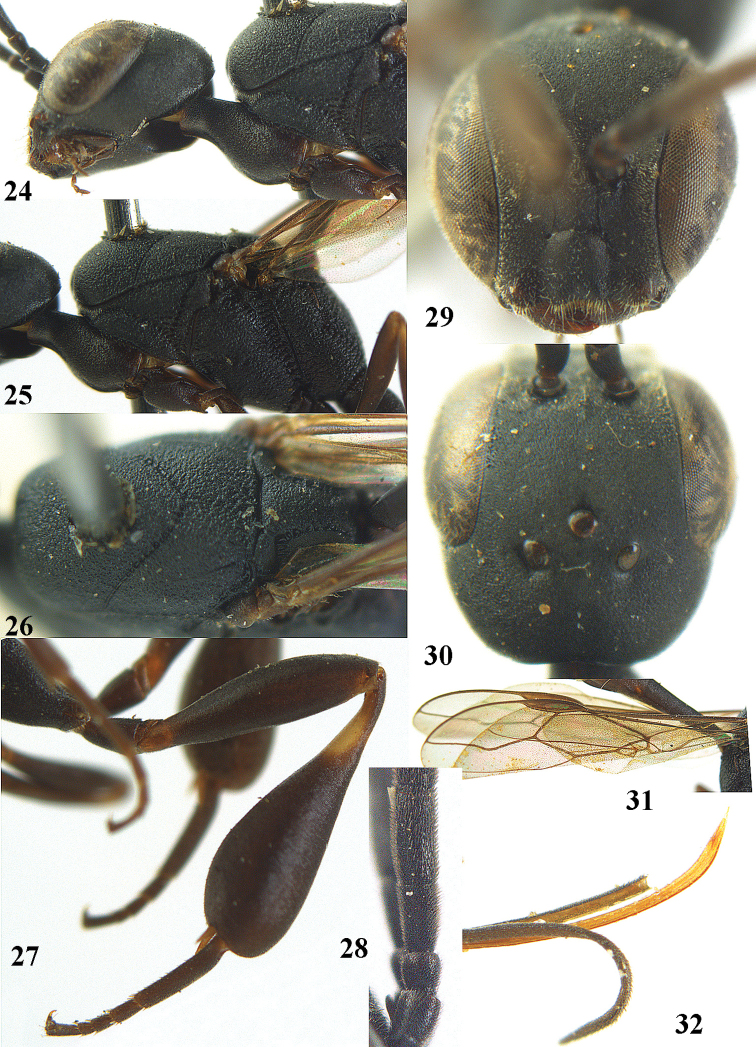
*Gasteruption boreale* (Thomson), female, holotype of *Gasteruption breviterebrae* (Watanabe). **24** head lateral **25** mesosoma lateral **26** mesonotum dorsal **27** hind leg **28** base of antenna **29** head anterior **30** head dorsal **31** fore wing **32** ovipositor and ovipositor sheath lateral.

(after Johansson and van Achterberg submitted) Head in dorsal view subparallel-sided behind eyes (Fig. 30), elongate, about as wide as long; occipital carina indistinct medio-dorsally; frons with satin sheen; mesoscutum rather weakly rugulose-coriaceous or chagreened, similar as vertex (Fig. 24) and with satin sheen, in front of scutellum rather rugose (Fig. 26); mesosoma and head silvery pilose; mesosoma with a satin sheen, quite distinct from the rather fatty gloss present in *Gasteruption assectator* s.s.; whitish pubescence of eye of female (Fig. 29) mostly distinctly longer and denser than of *Gasteruption assectator* s.s.; antenna slightly shorter than in *Gasteruption assectator* s.s. with sixth segment about 1.5 times longer than wide and subapical segment about 1.2 times longer than wide; only apical half of hind coxa weakly striate dorsally; hind tibia and basitarsus with white or ivory ring (Fig. 27); metasoma mainly black with orange lateral patches on tergites 2–4, which might be partially reduced (Fig. 23); inner sides of tibiae often red brown to orange with white or yellow basal patch indistinct on fore and middle tibiae; ovipositor sheath black or brown, 0.7–1.0 times as long as hind tibia, its apical half entirely with stout, rather scarce black bristles angled backwards at about 45° (Fig. 32). The male is difficult to separate from males of *Gasteruption assectator* s.s. and identification is not always possible with certainty. In most cases males of *Gasteruption boreale* have a slightly more elongate and subparallel-sided head in dorsal view, a less sculptured mesoscutum and a more or less enlarged malar space.

#### Distribution.

**China** (*Heilongjiang, ZJUH); **Russia** (Sakhalin). New for China.

#### Biology.

Unknown. Collected May-July.

### Gasteruption huangshii

Taxon classificationAnimaliaHymenopteraGasteruptiidae

Tan & van Achterberg sp. n.

http://zoobank.org/6C7EC527-A23F-4AA1-B9A9-76E85FD24445

Figs 33 , 34–42 , 43–48

#### Type material.

Holotype, ♀ (NWUX), “China: Shaanxi, Hanzhong, Liuba, Zhang Liang Temple, N33.68° E106.83°, 28.vii.2015, 1348 m, Jiangli Tan & Qingqing Tan”. Paratypes (NWUX, RMNH): 5♂, same data as holotype.

#### Comparative diagnosis.

The new species runs in the key by Zhao et al. (2012) to *Gasteruption japonicum* Cameron and *Gasteruption sinepunctatum* Zhao, van Achterberg & Xu, because of the very finely sculptured mesoscutum. It differs from both species by the trapezoid head in dorsal view (Fig. 40 vs Figs 55, 102), the distinctly widened hind tibia, fore and hind femora (Fig. 41 vs Figs 53, 103) and the slender head in anterior view (Fig. 39 vs Figs 54, 101). It shares with *Gasteruption praestans* Semenov-Tian-Shanskij & Kostylev, 1928, from Kazakhstan the widened hind femur and the sparsely punctate vertex. It differs by the ivory apical part of the ovipositor sheath (absent in *Gasteruption praestans*), the hind femur and basitarsus robust (slimmer), the mesoscutum finely coriaceous with fine punctures (coarsely and rather densely punctate) and the mesopleuron mainly coriaceous dorsally and posteriorly (reticulate).

**Figure 33.**
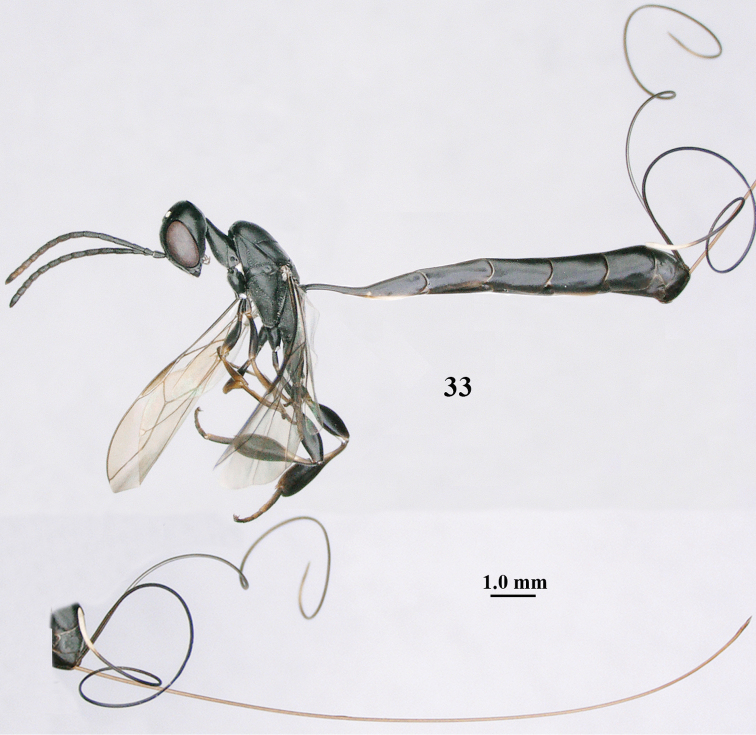
*Gasteruption huangshii* Tan & van Achterberg, sp. n., female, holotype, habitus lateral.

**Figures 34–42.**
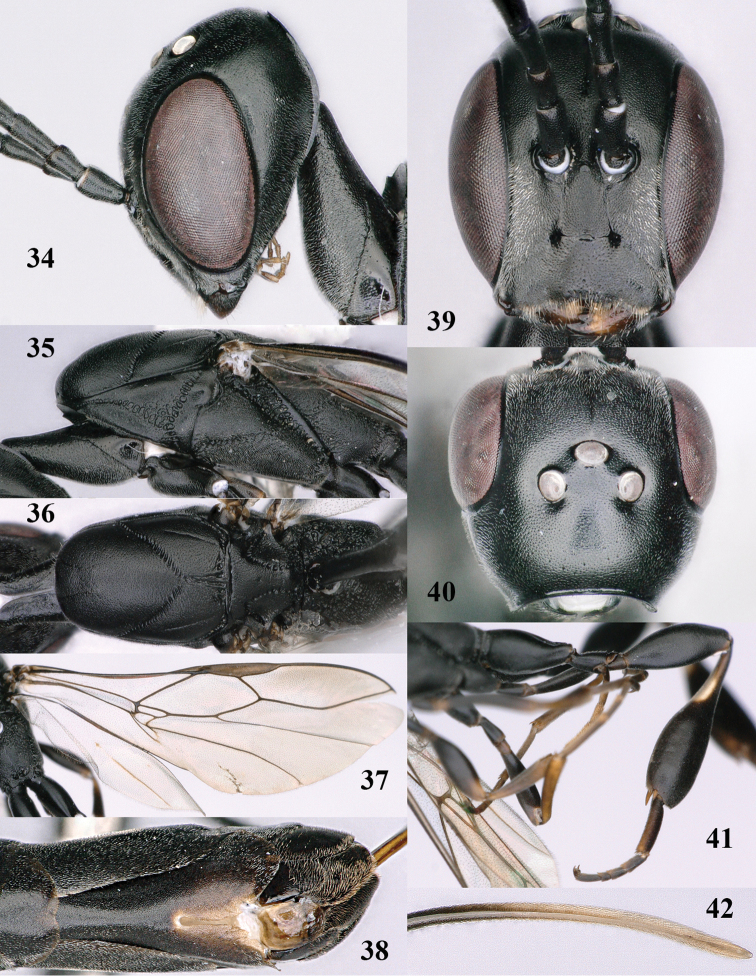
*Gasteruption huangshii* Tan & van Achterberg, sp. n., female, holotype. **34** head lateral **35** mesosoma lateral **36** mesonotum dorsal **37** fore wing **38** hypopygium ventral **39** head anterior **40** head dorsal **41** hind leg **42** apex of ovipositor sheath.

#### Description.

Holotype, female, length of body 12.5 mm, of fore wing 6.0 mm.

*Head*. Vertex and frons with satin sheen, finely and densely punctulate (but vertex with some fine additional punctures: Fig. 40), moderately convex and without a depression medio-posteriorly; head trapezoid and gradually narrowed behind eyes in dorsal view and temples convex (Fig. 40); temple 0.7 times as long as eye in dorsal view; fourth antennal segment 1.5 times as long as third segment and 0.9 times as long as second and third segments combined, fifth antennal segment 1.2 times as long as third segment (Fig. 33), third antennal segment 1.4 times as long as second segment; occipital carina narrow and narrowly lamelliform medio-dorsally (Figs 34, 40); OOL 1.3 times as long as diameter of posterior ocellus; face 2.7 times wider than high, 2.4 times wider than eye in anterior view (Fig. 39); minimum width of malar space 0.3 times as long as second antennal segment (Fig. 34); clypeus rather flat and with small round emargination medio-ventrally; eye glabrous.

*Mesosoma*. Length of mesosoma 2.1 times its height; propleuron rather robust and 0.9 times as long as mesoscutum in front of tegulae; pronotal side entirely punctulate (except for crenulated grooves and some fine punctures ventrally) and sparsely setose, with minute lobe-shaped tooth antero-ventrally (Fig. 35); antesternal carina narrow and hardly lamelliform; mesosternal sulcus narrow anteriorly and moderately wide posteriorly; mesopleuron mainly superficially coriaceous dorsally and posteriorly; mesoscutum and scutellum matt, very finely and superficially coriaceous with fine punctures and medio-posteriorly with some short grooves (Fig. 36); propodeum rugose anteriorly and coriaceous posteriorly.

*Wings*. First discal cell parallel-sided and with outer posterior corner rounded, and with vein 3-CU1 near apical third (Fig. 37).

*Legs*. Hind coxa very finely coriaceous-punctulate; length of hind femur, tibia and basitarsus 2.7, 3.5 and 4.1 times their width, respectively; middle tarsus 1.1 times as long as middle tibia; middle femur subparallel-sided and slimmer than distinctly widened fore femur.

*Metasoma*. Ovipositor sheath 15.3 mm, 1.2 times longer than body, 1.7 times as long as metasoma and 7.5 times as long as hind tibia, ivory apical part of sheath 1.8 times as long as hind basitarsus; apical half of hypopygium incised (Fig. 38).

*Colour*. Black; mandible dark brown with middle part brown; fore femur apically, fore and middle tibiae basally and apically, and hind tibial spurs yellowish brown; hind tibia ventro-basally ivory; tegulae and remainder of legs mainly dark brown; pterostigma dark brown; wing membrane subhyaline.

*Male*. Similar to female, but head behind eye slightly more contracted in dorsal view and somewhat shorter (Fig. 47); third antennal segment as long as second segment, fourth antennal segment 2.5–2.9 times as long as third segment and 1.3–1.5 times as long as second and third segments combined, fifth antennal segment 2.6–2.9 times as long as third segment (Fig. 46); mouthparts ivory; paramere densely whitish setose and its apex brownish yellow (Fig. 48).

**Figures 43–48.**
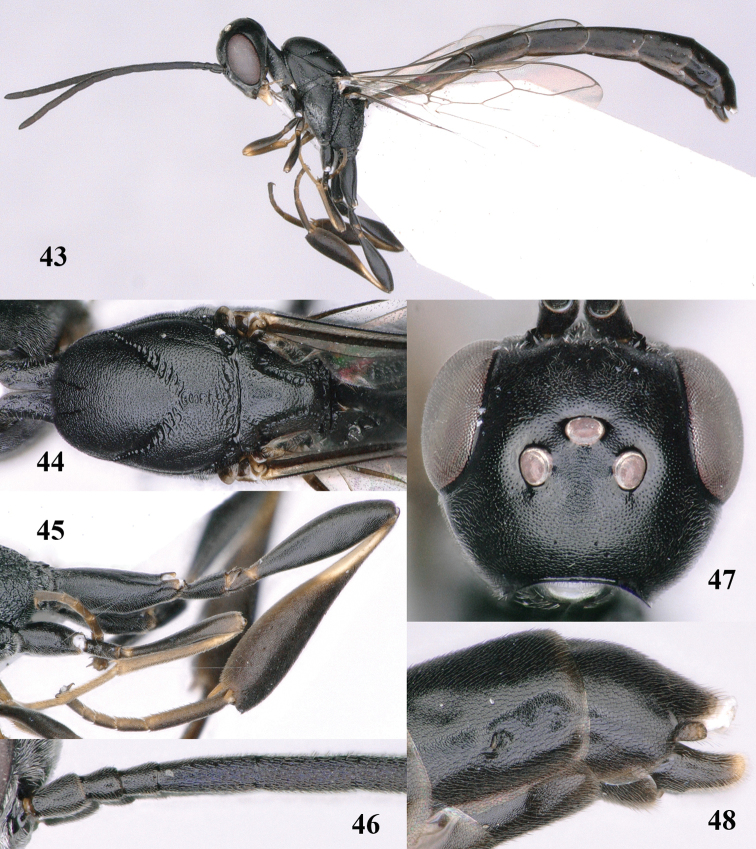
*Gasteruption huangshii* Tan & van Achterberg, sp. n., male, paratype. **43** habitus lateral **44** mesosoma dorsal **45** hind leg **46** basal antennal segments **47** head dorsal **48** apex of metasoma lateral.

*Variation*. Body length of male 8.7–9.9 mm; sculpture of mesosoma of male very fine and only slightly coarser coriaceous than of female.

#### Distribution.

China (Shaanxi).

#### Biology.

Unknown, but the new species was collected together with a *Hylaeus* sp.

#### Etymology.

Named after Huang Shi Gong (supposed teacher of the early Han general Zhang Liang), because the specimens were collected outside the hall with Huang Shi Gong’s statue at the Zhang Liang Temple.

### Gasteruption japonicum

Taxon classificationAnimaliaHymenopteraGasteruptiidae

Cameron, 1888

Figs 49 , 50–57

Gasteruption japonicum Cameron, 1888: 134; Zhao et al. 2012: 58–61.
Gasteruption sinense var. minus Kieffer, 1924: 78; Zhao et al. 2012: 58 (synonymized with Gasteruption japonicum).

#### Material.

2♀ (NWUX, RMNH), China: Shaanxi, Hanzhong, Nanzheng, Liping National Forest Park, N32°44'04” E106°36'34”, 22.vi.2015, c 1620 m, Jiangli Tan & C. van Achterberg; 1♀ (NWUX), China: Shaanxi, along the road from Hanzhong to Liping, N32.87° E106.71°, 4.ix.2015, c 1377 m, Jiangli Tan; 1♂ (NWUX), China: Hubei, Yichang, Yiling, Chentangping, Mal. trap, 17.v.10.vii.2015, c 465 m, Haoliang Ni; 1♀ (NWUX), China: Shaanxi, Foping, behind Biological Station, Malaise trap, N33°39'29” E107°48'25”, 29.v.19.vi.2016, c 1710 m, JL. Tan & C. v. Achterberg.

#### Notes.

The lectotype female of *Gasteruption rufescenticorne* Enderlein, 1913, is obviously different from the paralectotype male (e.g. head not emarginate medio-posteriorly and more narrowed, narrower occipital carina and different sculpture of mesoscutum) and is very similar to *Gasteruption japonicum*. The differences as indicated in the key could be part of gradual variation and after examination of more Japanese material its status may need reconsideration.

**Figure 49.**
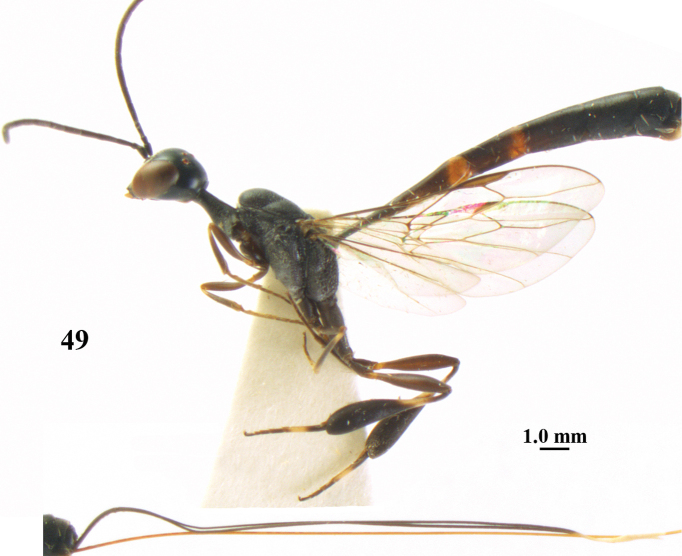
*Gasteruption japonicum* Cameron, female, Japan, habitus lateral.

**Figures 50–57.**
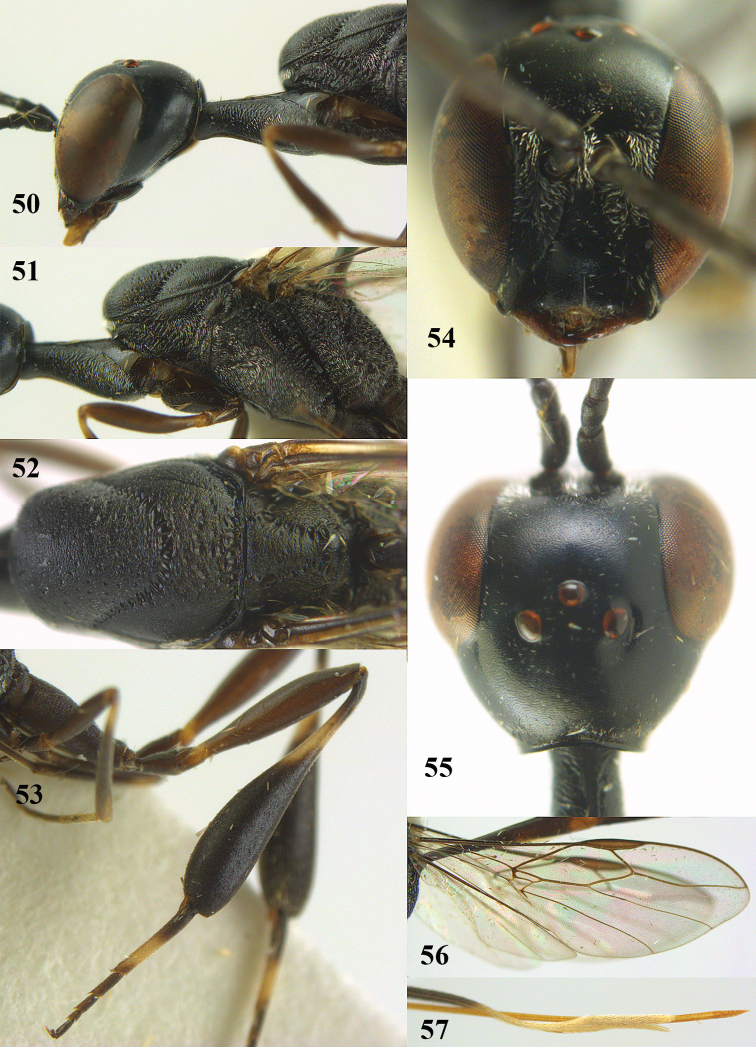
*Gasteruption japonicum* Cameron, female, Japan. **50** head lateral **51** mesosoma lateral **52** mesonotum dorsal **53** hind leg **54** head anterior **55** head dorsal **56** fore wing **57** apex of ovipositor sheath.

### Gasteruption oshimense

Taxon classificationAnimaliaHymenopteraGasteruptiidae

Watanabe, 1934

Figs 58 , 59–67

Gasteruption oshimensis Watanabe, 1934: 283–284.
Gasteruption tournieri ; Zhao et al. 2012: 103–108.

#### Material.

2♀ (NWUX), China: Shaanxi, Zhashui, Huanghualing., N33.76° E108.85°, 23.vii.2015, c 1577 m, Jiangli Tan; 2♂ (NWUX), China: Shaanxi, Hanzhong, Liuba, Zibai Mt. Nat. Res., N33.66° E106.78°, 5.ix.2015, c 1627 m, Jiangli Tan; 3♀ 6♂ (NWUX, RMNH), China: SE Shaanxi, Langoa near Ankang, N32°17'01” E109°03'46”, c 1100 m, Jiangli Tan, Qingqing Tan & C. van Achterberg; 1♂ (NWUX), China: Shaanxi, Foping, behind Biological Station, Malaise trap, N33°39'29” E107°48'25”, 29.v.19.vi.2016, c 1710 m, JL. Tan & C. v. Achterberg.

#### Notes.

The East Palaearctic specimens provisionally identified as *Gasteruption tournieri* Schletterer, 1885, by Zhao et al. (2012) are included here under *Gasteruption oshimense* Watanabe. The different shape of the head was noticed before, but also the hind tibia and hind basitarsus are slimmer and the sculpture of the mesoscutum is less developed. Most likely it concerns a separate species and because a valid name is available, this name (correctly spelled as *Gasteruption oshimense*) is used here. Especially the size of the males is very variable, e.g. length of body is 5.3–8.7 mm in the series from Langoa collected at the same spot and within one hour.

**Figure 58.**
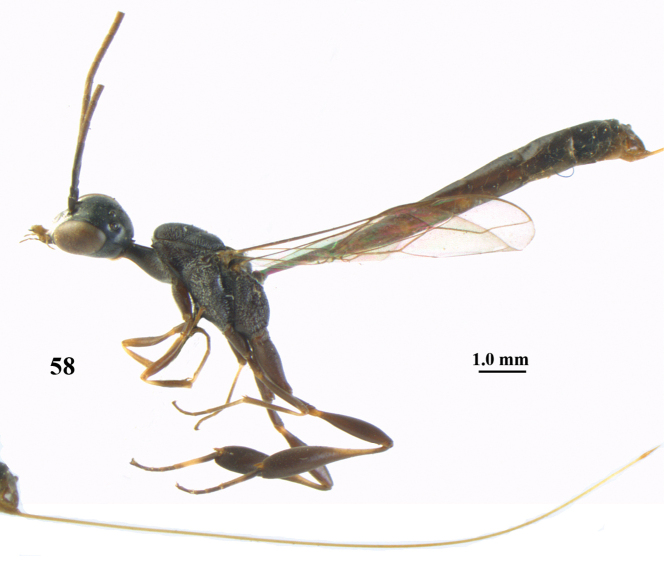
*Gasteruption oshimense* Watanabe, female, lectotype, habitus lateral (ovipositor sheath missing).

**Figures 59–67.**
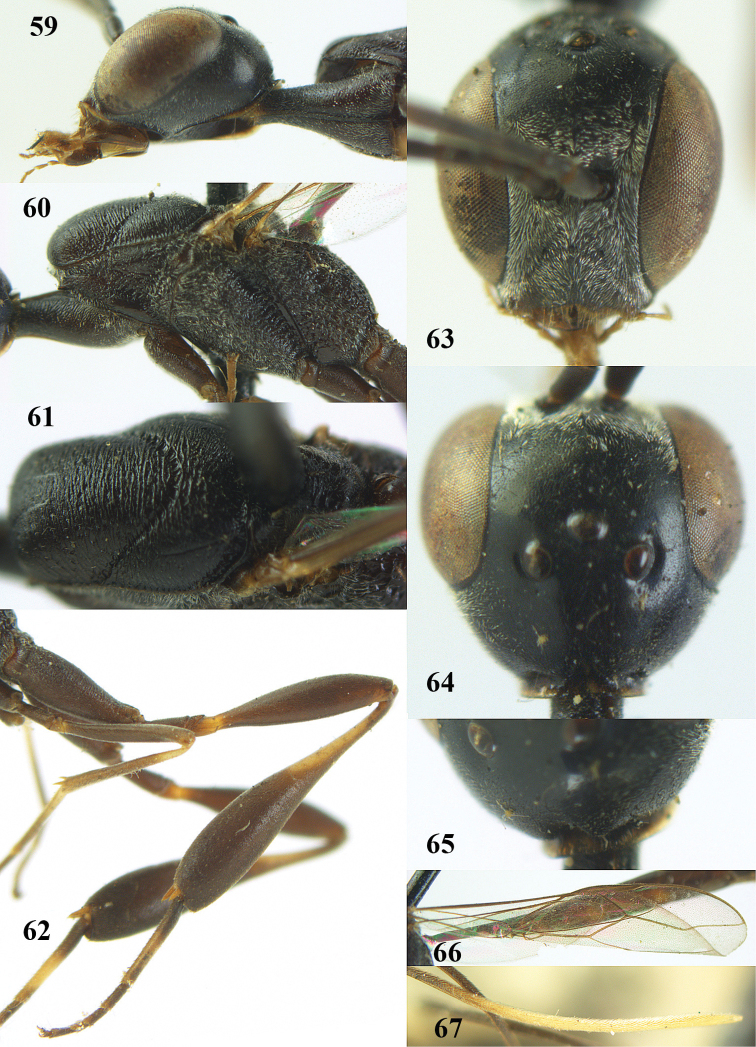
*Gasteruption oshimense* Watanabe, female, lectotype. **59** head lateral **60** mesosoma lateral **61** mesonotum dorsal **62** hind leg **63** head anterior **64** head dorsal **65** occipital carina dorsal **66** fore wing **67** apex of ovipositor sheath.

### Gasteruption pannuceum

Taxon classificationAnimaliaHymenopteraGasteruptiidae

Tan & van Achterberg sp. n.

http://zoobank.org/2E182575-5709-470C-81D6-435C2AE0C469

Figs 68 , 69–77

#### Type material.

Holotype ♀ (NWUX), China: Shaanxi, Qinling Mts., Baolongyu, N34°03' E108°09', c 700 m, 10.vi.2015, 24.v.2015, Jiangli Tan.

#### Comparative diagnosis.

Runs in Zhao et al. (2012) either to *Gasteruption varipes* (Westwood, 1851) (if the pale apical part of the ovipositor sheath is considered to be minor) or to *Gasteruption sinarum* Kieffer, 1911 (if the pale part is considered to be intermediate; Fig. 77). The new species differs from *Gasteruption varipes* by having the mesopleuron black and finely sculptured (orange brown and coarsely vermiculate-reticulate (rarely only weakly so)), the mesoscutum slender and finely rugulose (robust and coarsely rugose), the propodeum mainly coriaceous (coarsely vermiculate-rugose), a shorter ovipositor sheath (3 times hind tibia vs 5 times) and the mandible brownish yellow (blackish). The new species differs from *Gasteruption sinarum* by having a shorter ovipositor sheath (3.2 times hind tibia vs 4.8–6.0 times in *Gasteruption sinarum*), the mesoscutum without coarse punctures (present), the vertex shiny and largely smooth (with satin sheen and punctulate in *Gasteruption sinarum*) and the vertex distinctly convex (less so in *Gasteruption sinarum*). It shares with *Gasteruption parvicollarium* Enderlein, 1913, the convex vertex, but the new species has a longer ovipositor sheath (3.1 times hind tibia vs 1.2–1.7 times in *Gasteruption parvicollarium*), the mesoscutum transversely wrinkled (mainly coriaceous) and eyes more conspicuously setose.

#### Description.

Holotype, female, length of body 10.0 mm, of fore wing 6.2 mm.

*Head*. Vertex and frons shiny and very finely punctulate, nearly smooth (Fig. 75), distinctly convex (Fig. 69) and without a depression medio-posteriorly; head trapezoid and directly narrowed behind eyes in dorsal view (Fig. 75); temple 0.7 times as long as eye in dorsal view; fourth antennal segment 1.1 times as long as third segment and 0.6 times as long as second and third segments combined, fifth antennal segment 1.2 times as long as third segment, third antennal segment 1.4 times as long as second segment; occipital carina narrow and non-lamelliform medio-dorsally (Figs 69, 75); OOL 1.5 times as long as diameter of posterior ocellus; face 3.5 times wider than high, twice wider than eye in anterior view (Fig. 74); minimum width of malar space 0.2 times as long as second antennal segment (Fig. 69); clypeus rather flat, slightly depressed ventrally and shallowly emarginate medio-ventrally; eye densely setose (Fig. 74).

**Figure 68.**
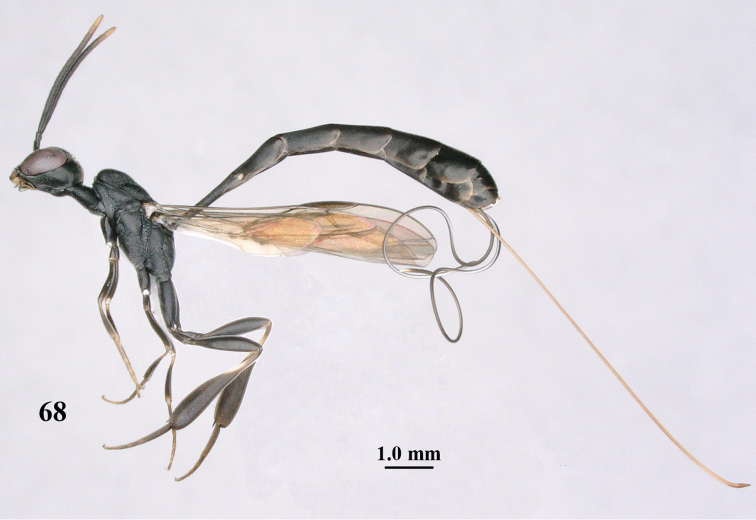
*Gasteruption pannuceum* Tan & van Achterberg, sp. n., female, holotype, habitus lateral.

**Figures 69–77.**
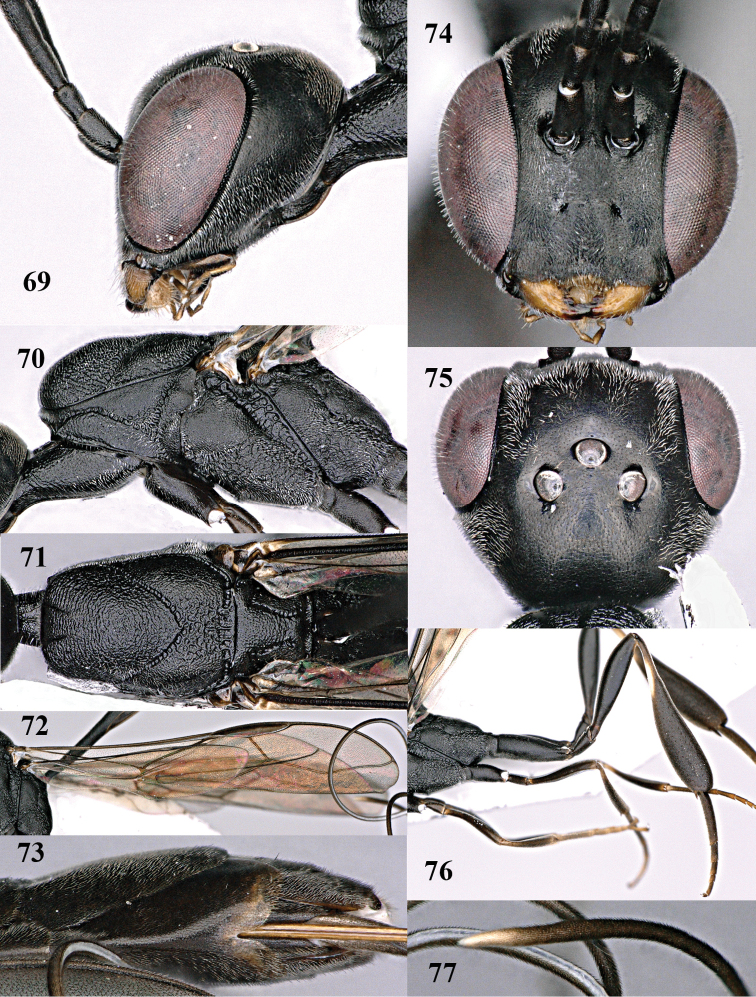
*Gasteruption pannuceum* Tan & van Achterberg, sp. n., female, holotype. **69** head lateral **70** mesosoma lateral **71** mesonotum dorsal **72** fore wing **73** hypopygium ventral **74** head anterior **75** head dorsal **76** hind leg **77** apex of ovipositor sheath.

*Mesosoma*. Length of mesosoma 2.1 times its height; propleuron rather robust and 0.9 times as long as mesoscutum in front of tegulae; pronotal side mainly coriaceous, but ventral half (except anteriorly) largely rugose and grooves crenulate and sparsely setose, with small blunt tooth antero-ventrally (Figs 70, 71); antesternal carina narrow and non-lamelliform; mesopleuron coriaceous and medially moderately rugose; mesosternal sulcus wide and crenulate; mesoscutum and scutellum with satin sheen, finely punctate-coriaceous, but middle lobe mainly transversely rugulose and medio-posteriorly broadly rugose (Fig. 71); propodeum mainly coriaceous but rugose anteriorly.

*Wings*. First discal cell parallel-sided and with outer posterior corner rounded, and with vein 3-CU1 near its apical third.

*Legs*. Hind coxa very finely coriaceous; length of hind femur, tibia and basitarsus 4.8, 5.1 and 5.1 times their width, respectively; middle tarsus 1.2 times as long as middle tibia; middle femur subparallel-sided and slightly slimmer than fore femur; hind tibia weakly inflated (Fig. 76).

*Metasoma*. Ovipositor sheath 8.5 mm, 0.8 times longer than body, 1.1 times as long as metasoma and 3.2 times as long as hind tibia, ivory apical part of sheath 0.3 times as long as hind basitarsus; apical 0.3 of hypopygium incised (Fig. 73).

*Colour*. Black; mandible brownish yellow and basally slightly darkened; apical antennal segment, apex of ovipositor sheath, fore and middle tibiae basally and hind tibia ventro-basally ivory or pale brown; penultimate antennal segment brown; tegulae, pterostigma, remainder of legs and antenna, hind tibial spurs and remainder of legs mainly dark brown or blackish; wing membrane subhyaline.

*Male*. Unknown.

#### Distribution.

China (Shaanxi).

#### Biology.

Unknown.

#### Etymology.

Named after the rugulose (“wrinkled”) sculpture of the middle lobe of the mesoscutum: “pannuceus” is Latin for “wrinkled”.

### Gasteruption shengi

Taxon classificationAnimaliaHymenopteraGasteruptiidae

Tan & van Achterberg sp. n.

http://zoobank.org/F8DE70E2-B352-411C-994F-FAC3E62B80BF

Figs 78–79 , 80–88 , 89–94

#### Type material.

Holotype, ♀ (NWUX), “China: Ningxia, Pingluo, Mt. Shizui, 25.v.2015, Mao-Ling Sheng”, “on *Hedysarum scoparium* Fisch ex Mey”. Paratypes: 1♀ 1♂ (RMNH), “N. China: Ningxia, Mt. Shizui, 6.v.2009, M.-L. Sheng, RMNH’11”; 1♂ (NWUX), “China: Inner Mongolia, Otog Banner, Yikebulage, 31.iii.2015, Mao-Ling Sheng”, “on *Tetraena mongolica* Maxim.” [translation of Chinese labels].

#### Comparative diagnosis.

Runs in Zhao et al. (2012) to *Gasteruption dimidiatum* Semenov, 1892, because of the emarginate head, the long and black ovipositor sheath, punctate mesoscutum and the finely sculptured propodeum. The new species differs from *Gasteruption dimidiatum* by having the head not prolonged below eyes in anterior view and malar space 0.2–0.3 times as long as second antennal segment (head shortly prolonged below eyes in *Gasteruption dimidiatum*; fig. 107 in Zhao et al. 2012, malar space 0.4 times as long as second antennal segment), first metasomal tergite black (orange or yellowish brown), basal half of hind coxa only coriaceous (transversely rugulose), apex of ovipositor sheath black (narrowly ivory), mesoscutum rather finely punctate (somewhat coarser punctate) and slightly wider hind tibia (slightly narrower). Males may be confused with *Gasteruption sinarum* Kieffer, 1911, the latter species has the hind coxa distinctly transversely rugose, the hind tibia is slim and the mesoscutum is more or less rugulose.

#### Description.

Holotype, female, length of body 13.1 mm, of fore wing 5.9 mm.

*Head*. Vertex and frons with satin sheen and very finely punctulate, but vertex posteriorly superficially coriaceous (Fig. 86), distinctly convex (Fig. 80) and without a depression medio-posteriorly; frons densely silvery setose anteriorly; head trapezoid and gradually narrowed behind eyes in dorsal view (Fig. 86); temple 0.7 times as long as eye in dorsal view; fourth antennal segment 1.4 times as long as third segment and 0.9 times as long as second and third segments combined, fifth antennal segment 1.1 times as long as third segment, third antennal segment 1.8 times as long as second segment; occipital carina narrow and hardly lamelliform medio-dorsally (Figs 80, 86); OOL 1.4 times as long as diameter of posterior ocellus; face 3 times wider than high, 2.2 times wider than eye in anterior view (Fig. 85); minimum width of malar space 0.2 times as long as second antennal segment (Fig. 80); clypeus rather flat, slightly depressed ventrally and distinctly emarginate medio-ventrally (Fig. 85); eye largely glabrous; head shallowly U-shaped emarginate posteriorly (Fig. 86).

**Figures 78–79.**
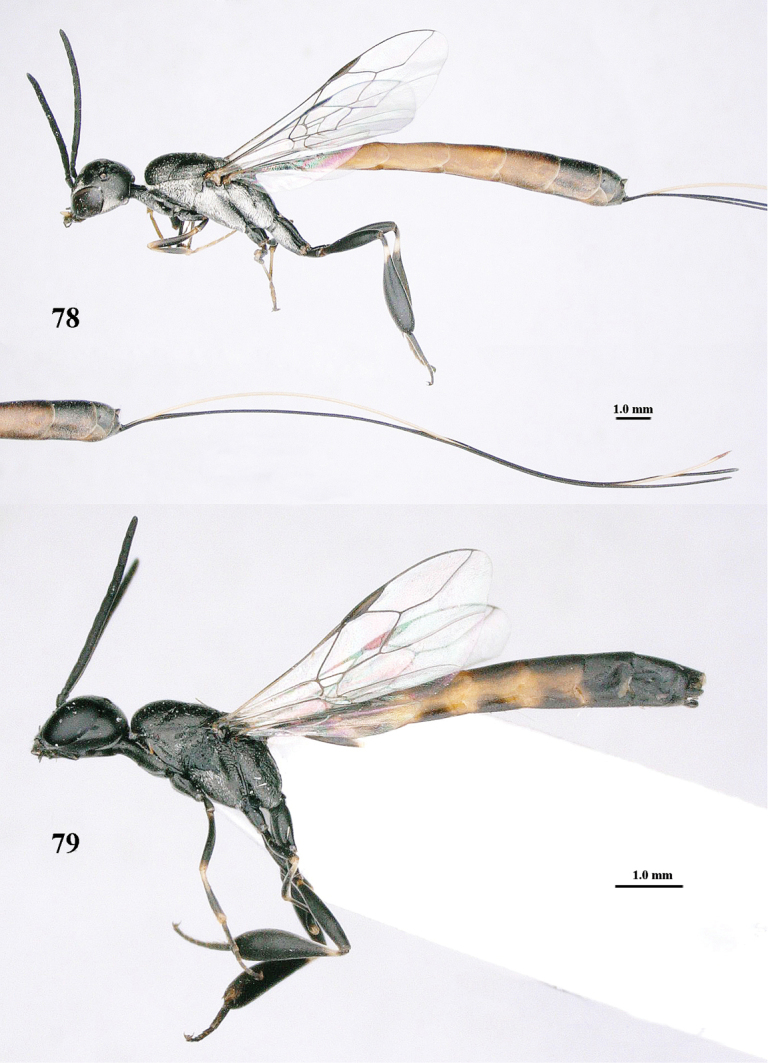
*Gasteruption shengi* Tan & van Achterberg, sp. n., female holotype (**78**) and male paratype (**79)**, habitus lateral.

**Figures 80–88.**
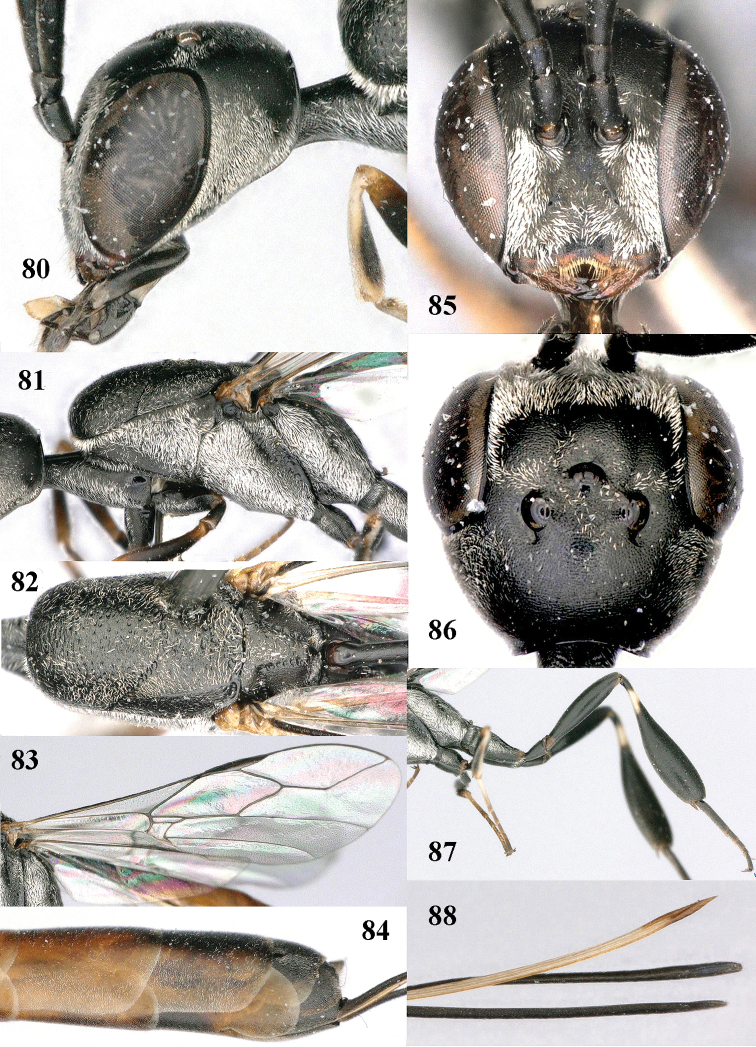
*Gasteruption shengi* Tan & van Achterberg, sp. n., female, holotype. **80** head lateral **81** mesosoma lateral **82** mesonotum dorsal **83** fore wing **84** hypopygium lateral **85** head anterior **86** head dorsal **87** hind leg **88** apex of ovipositor sheath.

*Mesosoma*. Length of mesosoma twice its height; propleuron rather robust and 0.8 times as long as mesoscutum in front of tegulae; pronotal side mainly superficially coriaceous, with grooves crenulate and largely densely silvery setose, with small acute tooth antero-ventrally (Fig. 80); antesternal carina narrow and non-lamelliform; mesopleuron coriaceous and largely densely silvery setose; mesosternal sulcus rather wide and crenulate; mesoscutum and scutellum with satin sheen, mesoscutum rather coarsely punctate but interspace mostly wider than diameter of punctures, interspaces superficially coriaceous, but middle lobe medio-posteriorly with few rugae (Fig. 82); scutellum mainly superficially coriaceous and with few small punctures; propodeum mainly coriaceous but medially with transverse crenulation connected to smooth median area.

*Wings*. First discal cell parallel-sided and with outer posterior corner rounded and with vein 3-CU1 near its apical third (Fig. 83).

*Legs*. Hind coxa very finely coriaceous and with satin sheen; length of hind femur, tibia and basitarsus 4.6, 4.7 and 5.3 times their width, respectively; middle tarsus 1.2 times as long as middle tibia; middle femur subparallel-sided and slightly slimmer than fore femur; hind tibia moderately inflated (Fig. 87).

*Metasoma*. Ovipositor sheath 14.4 mm, 1.1 times longer than body, 1.6 times as long as metasoma and 5.9 times as long as hind tibia, apex of sheath black; apical 0.5 of hypopygium incised (Fig. 84).

*Colour*. Black; mandible brown and basally slightly darkened; base and apex of fore and middle tibiae, most of fore and middle basitarsi and subbasal ring of hind tibia ivory or pale brown; tegulae, base and apex of fore and middle femora, remainder of fore and middle tarsi (but middle telotarsus dark brown), hind tibial spurs, secondfifth metasomal segments, apical half of hypopygium and lateral spots on sixth tergite brown; pterostigma, veins and clypeus ventrally dark brown; wing membrane subhyaline.

*Male*. Similar to female, but sculpture of mesoscutum coarser (Fig. 90), head less emarginate posteriorly and propodeum more or less reticulate; third antennal segment 1.3 times as long as second segment, fourth antennal segment 1.9–2.1 times as long as third segment and 1.1–1.2 times as long as second and third segments combined, fifth antennal segment 1.9–2.3 times as long as third segment (Fig. 93); mouthparts partly ivory; paramere greyish setose and its apex black (Fig. 94); hind tarsus mainly dark brown or blackish.

**Figures 89–94.**
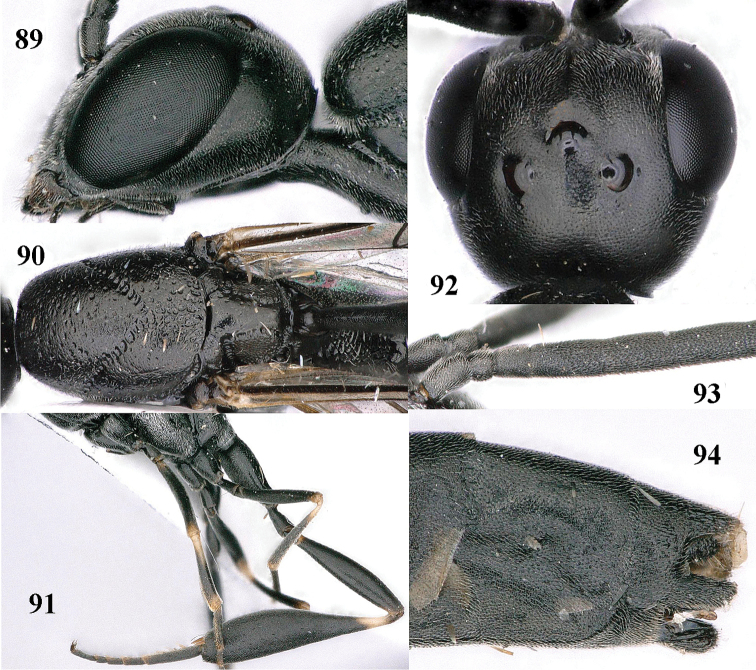
*Gasteruption shengi* Tan & van Achterberg, sp. n., male, paratype from Inner Mongolia. **89** head lateral **90** mesosoma dorsal **91** hind leg **92** head dorsal **93** basal antennal segments **94** apex of metasoma lateral.

*Variation*. Body length of female 12.1–13.1 mm, of male 10.1–11.5 mm; length of malar space 0.2–0.3 times as long as second antennal segment; propleuron 0.8–0.9 times as long as mesoscutum in front of tegulae; ovipositor sheath 10.5–14.4 mm, 0.9–1.1 times longer than body, 1.4–1.6 times as long as metasoma and 4.1–5.9 times as long as hind tibia; occipital carina of female paratype narrow lamelliform medio-dorsally, mandible rather yellowish brown, hypopygium and sixth tergite entirely brown and seventh tergite laterally so, fore and middle legs (except coxae and trochanters) mainly brown, tegulae dark brown, hind femur brownish black and subbasal ring of hind tibia brownish.

#### Distribution.

China (Ningxia, Inner Mongolia).

#### Biology.

Unknown.

#### Etymology.

Named after the collector, Prof. Dr Mao-Ling Sheng, for his contribution of our knowledge of Chinese parasitoid Hymenoptera.

### Gasteruption sinepunctatum

Taxon classificationAnimaliaHymenopteraGasteruptiidae

Zhao, van Achterberg & Xu, 2012

Figs 95 , 96–104

Gasteruption sinepunctatum Zhao, van Achterberg & Xu, 2012: 85.

#### Material.

1♀ (NWUX), China: Shaanxi, Hanzhong, Liuba, Zibai Mt. Nature Reserve, N33.66° E106.78°, 5.ix.2015, c 1627 m, Jiangli Tan.

#### Note.

Known in China from Jilin, Zhejiang, Taiwan and Tibet; new for Shaanxi.

**Figure 95.**
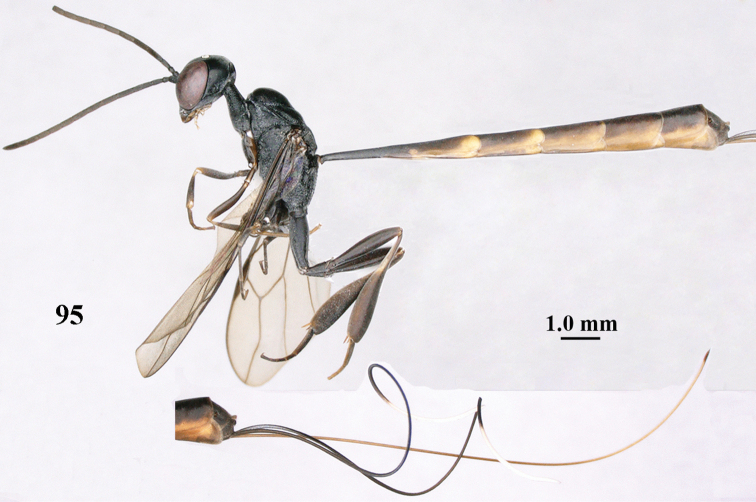
*Gasteruption sinepunctatum* Zhao, van Achterberg & Xu, female, Shaanxi, habitus lateral.

**Figures 96–104.**
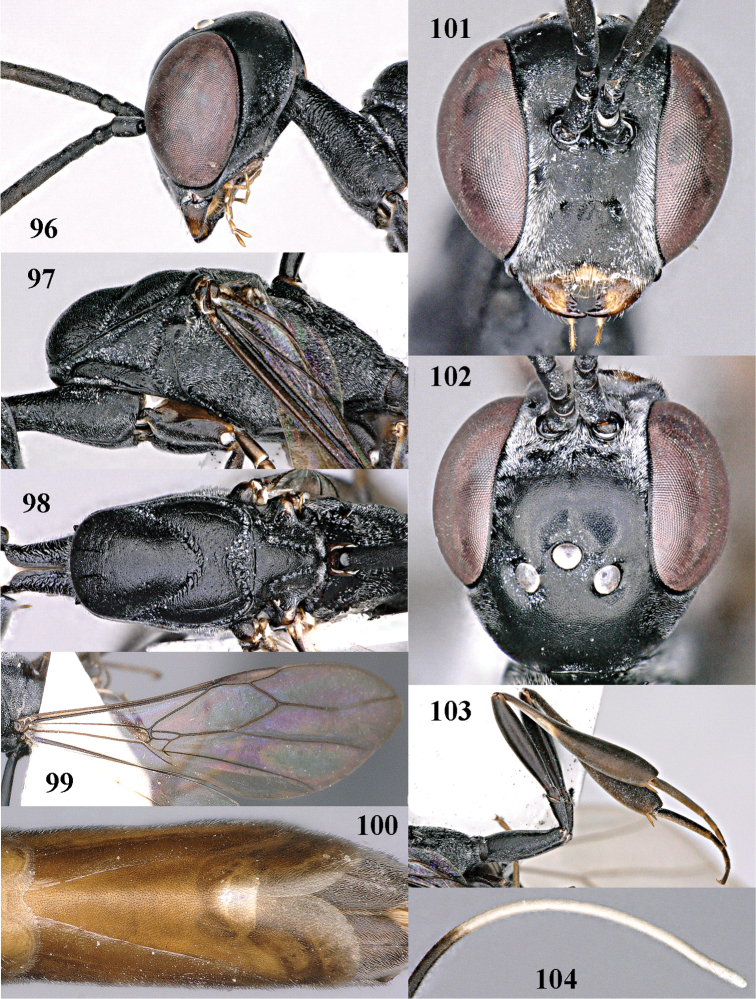
*Gasteruption sinepunctatum* Zhao, van Achterberg & Xu, female, Shaanxi. **96** head lateral **97** mesosoma lateral **98** mesonotum dorsal **99** fore wing **100** hypopygium ventral **101** head anterior **102** head dorsal **103** hind leg **104** apex of ovipositor sheath.

## Supplementary Material

XML Treatment for Gasteruption

XML Treatment for Gasteruption angulatum

XML Treatment for Gasteruption bicoloratum

XML Treatment for Gasteruption boreale

XML Treatment for Gasteruption huangshii

XML Treatment for Gasteruption japonicum

XML Treatment for Gasteruption oshimense

XML Treatment for Gasteruption pannuceum

XML Treatment for Gasteruption shengi

XML Treatment for Gasteruption sinepunctatum
